# Safety and Efficacy of Vaccination During Lactation: A Comprehensive Review of Vaccines for Maternal and Infant Health Utilizing a Large Language Model Citation Screening System

**DOI:** 10.3390/vaccines13040350

**Published:** 2025-03-25

**Authors:** Sien J. Mulleners, Hannah G. Juncker, Jan Zuiderveld, Kirsten A. Ziesemer, Johannes B. van Goudoever, Britt J. van Keulen

**Affiliations:** 1 Department of Pediatrics, Emma Children’s Hospital, Amsterdam UMC, University of Amsterdam, Meibergdreef 9, 1000 DE Amsterdam, The Netherlands; s.j.mulleners@amsterdamumc.nl (S.J.M.); h.juncker@amsterdamumc.nl (H.G.J.); b.j.vankeulen@amsterdamumc.nl (B.J.v.K.); 2 Department of Pediatrics, Amsterdam UMC, Vrije Universiteit van Amsterdam, de Boelelaan 1117, 1081 HV Amsterdam, The Netherlands; 3 Faculty of Natural Sciences, Mathematics and Informatics, Universiteit van Amsterdam, Amsterdam Science Park 904, 1098 XH Amsterdam, The Netherlands; jan@warana.xyz; 4 Medical Library, Vrije Universiteit, 1081 HV Amsterdam, The Netherlands; k.a.ziesemer@gmail.com

**Keywords:** vaccines, breastfeeding, side effects, immunity, antibodies, human milk

## Abstract

Newborns are born with an immature immune system, making them susceptible to infections early in life. Human milk provides essential nutrients and immunological factors that support infant immunity. Maternal vaccination during lactation has the potential to enhance these benefits by triggering an immune response in the mother, potentially extending protection to her child. However, lactating individuals are often excluded from vaccine trials, leading to uncertainties about vaccine safety and efficacy during the postpartum period. This study critically evaluates the effectiveness of vaccines in enhancing the immune-supporting properties of human milk and assesses their safety and efficacy for lactating mothers and their infants. By examining potential benefits alongside safety concerns, we aim to provide a comprehensive understanding of postpartum vaccination’s impact on maternal and infant health. We utilized large-language models (LLMs) to enhance the review process and performed a structured literature search across Ovid/Medline, Embase, and Clarivate Analytics using terms like “breastfeeding”, “postpartum”, and “vaccination”. A three-stage screening process involving human and LLM-assisted evaluation focused on postpartum vaccines and their implications for maternal and infant health. We identified 73 studies covering vaccines against COVID-19, cholera, influenza, pertussis, pneumococcal, rabies, polio, rotavirus, rubella, varicella, typhoid, smallpox, and yellow fever. Most vaccines, such as those for COVID-19 and influenza, appear safe and effective for postpartum use without requiring precautionary measures. However, caution is advised with vaccines such as the yellow fever vaccine, where temporary breastfeeding cessation is recommended. Overall, this review underscores the compatibility of most vaccines with lactation and suggests its benefits for both mother and infant.

## 1. Introduction

The benefits of human milk extend beyond simply delivering nutrition to infants. It also contains immunoglobulins, cytokines, immune cells, oligosaccharides, and other factors that can enhance the infant’s immune system and protect against infectious diseases. As the immune system of newborns is still developing, young infants are particularly susceptible to infectious diseases and serious complications arising from these infections [1].

Vaccinating lactating mothers is a promising strategy for enhancing infant immunity, in addition to offering protection to the mother. Maternal immunization not only reduces the likelihood of the mother contracting and transmitting infectious diseases to her infant, but it may also stimulate the secretion of specific antibodies into human milk. This process potentially provides passive immunity to the breastfed infant, offering an additional layer of protection [2,3].

However, vaccine hesitancy remains a significant issue among lactating women worldwide. Studies have accentuated the low vaccination rates for Influenza and Tdap (Tetanus, Diphtheria, Pertussis) vaccines during pregnancy and lactation [4], with an even more noticeable hesitancy toward the COVID-19 vaccine [5]. Factors contributing to this hesitancy include a lack of explicit information, misconceptions about vaccine safety, and fears concerning potential harm to both the mother and infant [6].

The initial exclusion of lactating women from vaccine trials has resulted in limited product-specific clinical data for these populations, which include mothers and their offspring. As a result, overly cautious guidelines from vaccine manufacturers, along with an absence of recommendations from healthcare professionals, have further exacerbated vaccine hesitancy.

Additionally, the spread of misinformation, particularly through social media and anti-vaccination movements, has undermined efforts to promote vaccination and combat infectious diseases [7]. Therefore, it is imperative to address these issues by providing accurate information and debunking misinformation or disinformation.

While vaccination recommendations for lactating women exist across various guidelines, our analysis revealed that guidance is (1) fragmented across multiple sources and (2) often relies on circular referencing between guidelines rather than primary evidence. Our review addresses these limitations by providing a single comprehensive resource that directly evaluates primary evidence, clarifying what is known, uncertain, or unstudied regarding vaccination during lactation. Healthcare providers play a pivotal role in promoting vaccination by offering evidence-based information regarding vaccine safety and efficacy. Professionals in the healthcare field wield significant influence in bolstering vaccine confidence; their recommendations have been identified as the foremost factor in this endeavor [8]. Despite the scarcity of clinical data, a great deal of the guidance available emerges from observational studies and post-licensure surveillance, highlighting the need for a comprehensive compilation and examination of the existing literature.

This comprehensive review aims to provide an in-depth analysis of the current evidence regarding postpartum vaccination for lactating women. The focus will be on the efficacy and safety of various vaccines for both mothers and infants. Additionally, it will address vaccine hesitancy, presenting strategies to manage those who decline vaccines. The psychological aspects pertinent to these discussions will also be considered [7].

While differences in study design and reporting may pose some limitations, this review aims to provide a consolidated resource for healthcare providers and policymakers. This comprehensive analysis could facilitate informed discussions about the advantages and disadvantages of receiving vaccinations during lactation.

### Objectives

The primary goal of this comprehensive review is to assess the impact of postpartum vaccination on maternal and infant health. Specifically, we aim to evaluate (1) the safety of vaccines in lactating individuals, (2) the safety of maternal vaccination for breastfed infants, (3) the immunogenicity of vaccines in lactating individuals, (4) the transfer of vaccine-induced immunity through human milk to infants, and (5) the clinical effectiveness of maternal vaccination in preventing disease in both mothers and infants. This evidence-based assessment aims to aid healthcare professionals in providing informed guidance, ultimately seeking to improve vaccination uptake and public health outcomes.

## 2. Methods

This comprehensive review was conducted following the Preferred Reporting Items for Systematic Reviews and Meta-Analysis Extension for Scoping Reviews (PRISMA-ScR) guidelines [9]. The review cannot be classified as an official systematic review since a large-language model (LLM) acted as the second reviewer. Traditional systematic review methodology requires two independent human reviewers for screening. The use of an LLM instead of a second human reviewer, while methodologically rigorous, deviates from this established standard, hence our classification as a comprehensive review rather than a systematic review.

### 2.1. Eligibility Criteria

Studies were chosen based on inclusion and exclusion criteria (Box 1). Studies were categorized for synthesis according to the vaccine (specific for a pathogen or disease) under investigation.

Box 1 **Inclusion and exclusion criteria.**

Inclusion criteria

▪ Lactating women receiving a vaccine during the postpartum period;
▪ Studies reporting on vaccine safety, efficacy, immune response, or transfer of vaccine components to human milk;
▪ Randomized controlled trials, prospective and retrospective cohort studies, cross-sectional studies, case reports, and case series;
▪ Peer-reviewed original research articles.

Exclusion criteria

▪ Studies investigating vaccines targeting non-infectious agents;
▪ Studies not reporting on vaccines administered during the postpartum period;
▪ Studies with insufficient data on outcomes of interest;
▪ Editorials, letters, legal cases, interviews;
▪ Non-English language studies (or no English translation version available).

### 2.2. Search Strategy

A comprehensive search strategy was created to identify pertinent studies on the administration of vaccines postpartum in lactating individuals. We explored three bibliographic databases (Ovid/Medline, Embase.com, and Clarivate Analytics/Web of Science Core Collection) from their inception until 7 July 2022. Additionally, we searched the Cochrane Library and Google Scholar and manually screened the reference lists of selected studies and pertinent reviews. These searches were strategized collaboratively with a medical information specialist (K.A.Z.). Search terms were devised using synonyms, closely related words, and keywords, either as index terms or free-text words, including “breast feeding”, “post partum”, and “vaccination”. No methodological filters were applied to the search strategy, and no date restrictions were set. The language was restricted to English publications or English translations of other-language publications as specified in our eligibility criteria (Box 1). We employed a three-stage de-duplication process: first using the R-package “ASYSD” for automated initial deduplication, followed by manual refinement in Endnote (X20.0.3) by a medical information specialist (K.A.Z.) to identify duplicates missed by the automated process, and finally a verification step in Rayyan (https://www.rayyan.ai/) by one of the reviewers (S.J.M.) during the screening phase to catch any remaining duplicates. Full search strategies can be found in Appendix A.

### 2.3. Selection Process

The study selection process occurred in three stages: initial screening, Large Language Model (LLM)-assisted screening, and full-text assessment.

1. Initial screening: In this phase, titles and abstracts were screened by a reviewer (S.J.M.) using Rayyan, a web-based automation tool [10]. This step aimed to eliminate clearly irrelevant studies based on pre-defined eligibility criteria.
2. LLM-assisted screening: For the secondary screening phase, we employed GPT-4 (version 40314), a LLM publicly accessible since June 2023. A custom-built system, created in collaboration with an artificial intelligence graduate (J.Z.), integrated the LLM into the screening process. This system was implemented in Python (version 3.8.17) and accessed via OpenAI’s application programming interface (API).
3. Full-text assessment: Finally, a full-text assessment was completed by a reviewer (S.J.M.) for studies categorized as ‘inclusion’ or ‘maybe’ by the LLM to ensure they met all inclusion criteria.

#### 2.3.1. LLM-Assisted Screening

The LLM-assisted screening system operated as follows:

1. Creating a python script with a command prompt in Visual Studio Code (version 1.80) to enable the LLM to independently access and screen all eligible studies (Appendix B).
2. For each study, the LLM evaluated the title and abstract using a detailed prompt (see Section 2.3.2).
3. The LLM provided a score (1–10 scale) for each eligibility criterion, along with a rationale for each score.
4. Based on these individual scores, the LLM gave an overall decision (inclusion, exclusion, or maybe) and a final score (1–10 scale). Appendix C contains an example of the LLM-citation screening output file, which includes the graded assessments and final decisions.
5. All LLM decisions were manually reviewed by a human reviewer to ensure accuracy.

Studies categorized as ‘inclusion’ or ‘maybe’ by the LLM were then subjected to full-text assessment by a human reviewer (S.J.M.). The scoring system and rationale from the LLM screening stage were used to facilitate this assessment.

#### 2.3.2. Prompt Engineering

The prompt for the LLM was formulated using the chain-of-thought prompting method [11], which breaks the assessment into several steps before specifying a final decision (Appendix B and Appendix C). This structured approach improved the LLM’s ability to apply complex reasoning [11,12,13,14]. The prompt was constructed by framing the LLM as a medical professor with expertise in lactation research tasked with evaluating article relevance based on predefined criteria. This role-based framing helped align the LLM’s perspective with the review’s objectives. For each criterion (population, intervention, outcome, and publication type), the prompt provided specific definitions, inclusion/exclusion parameters, and examples to guide assessment. For instance, under “Population,” the LLM was instructed to include studies with lactating individuals and/or their offspring while excluding those focusing on specific diseases or targeting non-infectious agents. Section 2.4 contained detailed examples of relevant outcomes in three categories: effects on lactating individuals, effects on breastmilk, and effects on offspring. The prompt also included explicit instructions for handling borderline cases, such as studies with indirect relevance to vaccination effects through breastmilk transfer. This comprehensive structure ensured consistent evaluation across citations while providing clear documentation of the reasoning process. The complete prompt is available in Appendix B, allowing for transparency and reproducibility of our methodology.

The prompt instructed the LLM to:

1. Evaluate each eligibility criterion (population, intervention, outcome, and publication type) separately.
2. Provide a one-sentence explanation and a relevance score (1–10 scale) for each criterion.
3. Give a final decision and overall score (1–10 scale) based on the individual criterion assessments.

The command prompt was developed and optimized through an iterative process using a set of 50 test cases sampled from the comprehensive dataset of eligible reports. A human reviewer and the LLM screening system independently assessed these test cases. Any discrepancies in judgment were meticulously evaluated, leading to prompt modifications to enhance the system’s sensitivity and specificity [11,12,13,14]. Examples of prompt modifications included refining the description of the population to explicitly incorporate studies involving both human and non-human subjects; expanding the outcome categories to encompass indirect effects of vaccines on offspring, such as immunization through breastmilk transfer; and adjusting the scoring system to assign more importance to studies focusing on multiple relevant outcomes. These modifications bolstered the LLM’s ability to identify pertinent studies and reduce false negatives, especially for studies with complex or multifaceted outcomes. The optimized command used for citation screening in the current comprehensive review can be accessed in Appendix B.

#### 2.3.3. Limitations and Challenges to LLM Utilization

Before incorporating an LLM into our study selection process, we scrutinized potential challenges we could encounter. We predicted issues such as the LLM possessing biases from its training data, the risk of it misinterpreting complex abstracts, and its dependency on the prompts we established. To preemptively tackle these challenges, we instituted a rigorous prompt engineering and optimization process, ensured all LLM decisions underwent manual review by a human expert and organized for continuous supervision and fine-tuning of the LLM screening process. Our goal was to utilize the advanced capabilities of an LLM while also effectively managing its limitations, thereby ensuring a thorough, efficient, and effective review process.

### 2.4. Data Collection/Items

During the collection of all pertinent outcome data from the included studies, the extracted information was discussed and verified by a second reviewer, B.J.v.K. The data extracted included study characteristics, participant demographics, vaccine characteristics, and outcome measures associated with recent vaccine administration in lactating individuals. Outcomes were grouped into five primary domains based on the effects of postpartum vaccination in mothers (see Box 2).

Box 2 **Outcome domains and subdomains.**

Effects of maternal postpartum vaccination

1. Safety in Lactating Individuals
2. Safety in Infants
  2.1. Viral shedding/Transmission vaccine components
  2.2. Adverse reactions in infants
3. Immunogenicity in Lactating Individuals
4. Infant Immunity Through Human Milk
  4.1. Human milk immune response
  4.2. Infant immune response
5. Vaccine Effectiveness
  5.1. Effectiveness in lactating individuals
  5.2. Effectiveness in infants

#### Outcome Definitions

Vaccine immunogenicity is defined as the ability of an antigen (a vaccine) to provoke an immune response in an individual. Vaccine efficacy represents the extent to which a vaccine yields beneficial results under ideal conditions. This is measured in phase III clinical trials by administering a vaccine to one group of people and comparing the incidence of disease with another group who did not receive the vaccine. Effectiveness conveys the degree to which a vaccine provides beneficial outcomes under optimal conditions [15]. The efficacy of vaccines gets tested in randomized, placebo-controlled studies, which are seldom conducted in lactating populations. Moreover, the data on the clinical impact of maternal vaccination is relatively scarce. Hence, in this review, we primarily concentrate on immunogenicity [15].

Vaccine safety concerns every adverse event (AE) that could potentially be caused, triggered, or intensified following the receipt of the vaccine. These include AEs like anaphylactic reactions, diseases diagnosed post-vaccination, and autoimmune events. Reactogenicity denotes a subset of reactions occurring shortly after vaccination and represents the vaccine-stimulated inflammatory response, which is often anticipated and actively investigated in clinical trials. Viral shedding following maternal postpartum vaccination refers to the excretion of the vaccine virus or vector in human milk or other maternal secretions, such as nasal secretions. This could pose a potential safety concern for breastfed infants due to possible transmission through breastfeeding [16].

In this review, we specifically investigate and describe the safety of lactating individuals and their infants post-maternal postpartum vaccination. We focus on the AEs that could affect breastfeeding and on the implications of viral shedding. Both exploratory and confirmatory adverse effects are explored, giving particular emphasis to the impact of viral shedding on a breastfed infant. Our pre-defined approach to reviewing adverse effects combines exploratory methodologies (opportunistic capture of any adverse effects) and confirmatory methodologies (focused on specific adverse effects: AEs related to breastfeeding/lactation, AEs in the infant) [17].

### 2.5. Study Risk of Bias Assessment

The risk of bias is evaluated at both the study level and the outcome level, with detailed assessments provided in Supplementary Tables S1 and S2.

At the study level, we employed standardized quality assessment tools tailored to specific study designs. For randomized controlled trials, observational cohort studies, case-control studies, before-after studies, and case series, we used the National Heart, Lung, and Blood Institute (NHLBI) quality assessment tools [18]. Each tool contains 12–14 questions evaluating key methodological components, including selection bias, information bias, confounding, and reporting quality. For case reports, we applied the Joanna Briggs Institute (JBI) Critical Appraisal Tool.

To evaluate the risk of bias at the outcome level, we used the Revised Risk of Bias tool (RoB 2) for randomized studies, assessing five domains: randomization process, deviations from intended interventions, missing outcome data, measurement of the outcome, and selection of reported results. For non-randomized studies, we employed the Risk of Bias in Non-randomized Studies of Interventions tool (ROBINS-I), which evaluates seven bias domains: bias due to confounding, selection of participants, and classification of interventions, as well as deviations from intended interventions, missing outcome data, measurement of the outcome, and selection of reported results [19,20]. Studies were categorized as having low, moderate, serious, or critical risk of bias based on domain-specific assessments. A single independent reviewer (S.J.M.) conducted the initial quality evaluation, with all assessments subsequently verified through discussion with a second reviewer (B.J.v.K.) to ensure reliability. Supplementary Tables S1 and S2 provide a comprehensive risk of bias assessments for each included study at both the study and outcome levels.

### 2.6. Effect Measures

The number of AEs is documented for the entire group of participants. Immunogenicity is characterized by the number of samples exhibiting detectable antibody levels or other immunological markers or by the number of samples demonstrating changes in levels of these markers. Vaccine effectiveness is reported contingent on the type of measures utilized in the study, which may include Odds Ratio, 95% confidence intervals, or *p*-values.

### 2.7. Data Synthesis

Given the anticipated heterogeneity of the included studies, a narrative synthesis approach will be implemented. This synthesis process involves organizing the data by vaccine type, summarizing findings for each vaccine, identifying trends and patterns across studies, comparing findings across different vaccine types, and emphasizing key findings and research gaps. The risk of bias assessments was systematically integrated into the evidence synthesis for each vaccine, which informed the level of evidence rating. Studies with a lower risk of bias were given greater weight in developing conclusions. Consideration of quality can be found within the vaccine chapters, which include a narrative description and a summarizing table on the evidence quality rating according to the GRADE approach, where we explicitly state how methodological limitations influenced our interpretation of findings and confidence in conclusions. Heterogeneity between the studies was addressed descriptively by illustrating their methodological differences (Supplementary Table S3).

### 2.8. Reporting Bias Assessment

The risk of bias due to missing results is explicitly mentioned in certain chapters on vaccines. Conclusions will be drawn about specific outcomes for explicitly named vaccines, provided the data is available.

### 2.9. Software and Data Management

During the review process, several software packages were used to ensure efficient and accurate data management, seamless integration of human-assisted and LLM-screening, and robust analysis of the included studies:

- Rayyan: Used for initial screening of titles and abstracts;
- OpenAI: GPT-4 (version 40314) was used as LLM;
- Python (version 3.8.17): Script enabling a LLM to access data and execute the command using an API;
- Visual Studio Code (version 1.80): Used to develop and run the LLM-screening command;
- CSV files: Used for data storage and transfer between software packages
- Zotero (versions 6.0.20–6.0.37): Used for reference management throughout the review process;
- Microsoft Excel (version 16.88): Used for data extraction and creation of summary tables.

## 3. Results

### 3.1. Results of the Search

**Figure 1:**
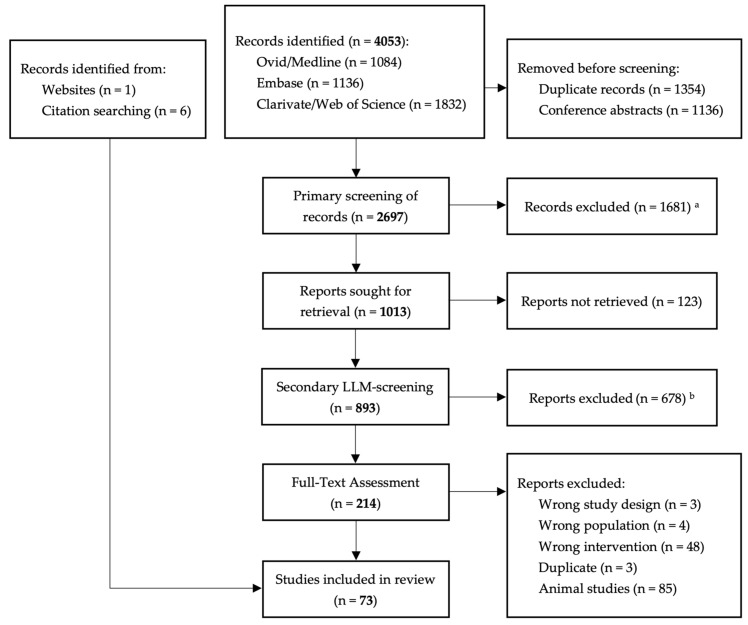
Flowchart of the search and selection procedure of studies. ^a^ Exclusion by human reviewer using Rayyan, an automation tool (see methodology for more information on screening process). ^b^ Exclusion by human reviewers utilizing a large language model.

The flow chart of the search and selection process is presented in Figure 1.

### 3.2. Included Studies

Seventy-three studies were incorporated into the final review. The literature retrieved spanned a variety of vaccines: COVID-19 vaccine (comprised of 41 articles), cholera (six studies), influenza (two studies), pertussis (Tdap) (three studies), typhoid (two studies), pneumococcal (one cohort study), rabies (one case report), rotavirus (one randomized-controlled trial (RCT)), rubella (seven studies), varicella (three studies), smallpox (one case report), yellow fever (six studies), and poliovirus (one cohort study). The literature retrieved on each of these vaccines will be discussed separately. Table 1 provides a summary of the studies included for each vaccine and outcome measure. Detailed characteristics of all included studies are presented in Supplementary Table S3, which includes information on study design, population, interventions, outcome measures, and key findings.

Table 2 provides a comprehensive summary of evidence and recommendations regarding vaccination during breastfeeding. This table synthesizes findings across all vaccines reviewed in this study, enabling direct comparison of safety profiles, immunological responses, and clinical recommendations. For each vaccine, we present a standardized assessment of evidence quality using the GRADE approach, along with clear recommendations and special considerations for clinical practice. This structured overview complements the detailed narrative discussions that will follow in the following Section 3.3, Section 3.4, Section 3.5, Section 3.6, Section 3.7, Section 3.8, Section 3.9, Section 3.10, Section 3.11, Section 3.12, Section 3.13, Section 3.14 and Section 3.15.

### 3.3. Cholera Vaccine

#### 3.3.1. Background

Cholera is an acute diarrheal disease with a high fatality rate. It is an infectious disease caused by the gram-negative *Vibrio cholerae* bacterium, which can lead to symptoms such as profuse, watery diarrhea, vomiting, rapid dehydration, and potentially life-threatening complications within several hours. Transmission occurs through contaminated water and food. Prevention can be achieved through a safe water supply, adequate sanitation and hygiene, treatment, and vaccination [94]. Vaccination is typically recommended for those traveling to or residing in a region that is endemic with cholera, experiencing a cholera outbreak, or in the midst of a humanitarian crisis. Currently, various oral, inactivated cholera vaccines are available worldwide. These vaccines contain killed whole cells of *V. cholerae* (WC), either alone or in combination with a recombinant B-subunit of cholera toxin (WC-BS) [94].

#### 3.3.2. Included Studies

Six studies assessed the immunogenicity of postpartum cholera vaccinations in a total of 709 lactating participants. The reviewed studies investigated various types of inactivated cholera vaccines, either using killed *V. cholerae* whole cell alone (WC) or combined with toxin B-subunit (WC-BS), for oral consumption [43,48] or administered subcutaneously [44,45,46,47]. None of the studies reported any maternal or infant AEs.

#### 3.3.3. Outcomes

I. Safety in Lactating Individuals—Not studied.

II. Safety in Infants—Not studied.

**III. Immunogenicity in Lactating Individuals**: Two randomized-controlled studies, along with three observational cohort studies, collectively demonstrated the potential of both oral and parenteral cholera vaccines to elicit significant immunogenic responses in lactating individuals [43,44]. Notably, observational studies revealed that prior natural exposure to cholera raises baseline antibody levels and post-vaccination responses, suggesting the crucial role of pre-existing immunity in enhancing vaccine immunogenicity [45,46,47]. This accentuates the need for further research into demographic- and exposure-related factors that modify vaccine immunogenicity.

**IV. Infant Immunity Through Human Milk:** The evidence on vaccine biomarker efficacy for breastfed infants primarily pertains to human milk antibodies post-cholera vaccination. Studies have indicated significant increases in secretory immunoglobulin A (SIgA) levels in the human milk of mothers who were naturally exposed previously [44,46,47]. However, substantial variations in design, population, and reporting within the non-randomized studies raise concerns about their robustness [45,46,47].

V.1 Effectiveness in Lactating Individuals—Not studied.

**V.2 Effectiveness in Infants:** The evidence regarding the efficacy of maternal vaccination in reducing severe cholera in infants was drawn from a case-control study nested within a randomized trial, which pointed to a potentially protective effect [48]. There were 270 infants included in the analysis, and an adjusted odds ratio of 0.47 (95% CI 0.22–1.00) was found, implying that maternal vaccination might reduce the risk of severe cholera in infants by nearly half, although the statistical significance of this result was marginal (*p* = 0.05).

#### 3.3.4. Quality of the Evidence

The GRADE evidence summary (Table 3) provides a transparent assessment of the certainty of evidence across outcomes for cholera vaccination during lactation.

There is a noted absence of data regarding vaccine safety for both lactating individuals and their infants. High certainty evidence supports the immunogenicity of oral cholera vaccines in lactating individuals, while moderate certainty evidence supports parenteral vaccine immunogenicity despite methodological limitations (small sample sizes, risk of bias due to confounding factors such as prior antigen exposure and demographic differences). For infant immunity, the evidence offers moderate certainty for benefits from maternal oral vaccination but very low certainty for parenteral vaccines due to inconsistencies and indirectness. The effectiveness data suggesting potential infant protection is of low certainty, limited primarily by statistical imprecision in the results.

#### 3.3.5. Conclusion

Overall, indications of the potential benefits of cholera vaccines administered during lactation exist, yet significant research gaps, especially in safety and direct infant immune responses, underscore the need for more comprehensive studies. Such research would fully establish their efficacy and safety in postpartum contexts.

### 3.4. Typhoid Vaccine (Typhoid Fever)

#### 3.4.1. Background

Typhoid fever is an acute illness caused by the bacterium Salmonella enterica serovar Typhi. The disease is highly prevalent in low- and middle-income areas such as South/Southeast Asia and sub-Saharan Africa, impacting 11 to 21 million people each year. The disease primarily spreads through the fecal-oral route, particularly in regions with inadequate sanitation. In areas where the disease burden and antimicrobial resistance are high, vaccination is prioritized. Currently, there are three vaccines available for typhoid fever, which include a conjugate vaccine for children aged 6 months to 2 years, a polysaccharide vaccine for children aged 2 to 6 years, and a live-attenuated Salmonella Typhi 21a vaccine for individuals aged 6 years and older. It is important to note that live-attenuated vaccines should not be administered during pregnancy, while guidelines regarding their use during lactation remain unclear [95].

#### 3.4.2. Included Studies

Two studies focused on postpartum typhoid vaccination using an oral, live-attenuated Salmonella Typhi 21a vaccine were analyzed [44,45]. Each study assessed the immune response in the serum and human milk of lactating individuals post-vaccination. The results were based on unique comparisons involving the oral typhoid vaccine alone, the parenteral-killed cholera vaccine alone, or the simultaneous administration of both vaccines.

#### 3.4.3. Outcomes

I. Safety in Lactating Individuals—Not studied.

II. Safety in Infants—Not studied.

**III. Immunogenicity in Lactating Individuals**: In a RCT involving ten participants, 90% exhibited increased serum immunoglobulin G (IgG) levels, while 50% demonstrated elevated serum immunoglobulin (IgA) levels after only receiving the oral typhoid vaccine. A lower IgG response was observed when a parenteral cholera vaccination was administered simultaneously [44]. In a separate observational cohort study involving six participants, it was reported that 67% of participants exhibited increased dimeric IgA levels after receiving both parenteral cholera and oral typhoid vaccines concurrently [45].

**IV. Infant Immunity Through Human Milk**: Limited evidence suggests that postpartum maternal typhoid vaccination may stimulate a mucosal antibody response in lactating individuals, potentially leading to increased levels of specific sIgA in human milk [44,45].

V. Effectiveness in Lactating Individuals or Infants—Not studied.

#### 3.4.4. Quality of the Evidence

Table 4 provides a transparent assessment of the evidence quality for typhoid vaccination during lactation.

The immunogenicity evidence in lactating individuals is classified as low certainty, starting with high-quality study designs but downgraded due to small sample sizes and methodological concerns. For infant immunity through human milk, the very low certainty rating reflects compounding limitations in the observational studies. The absence of any safety data for both mothers and infants represents a critical research gap, as does the lack of clinical effectiveness studies. These significant knowledge gaps highlight priority areas for future research before definitive recommendations can be made.

#### 3.4.5. Conclusion

The findings imply that the oral, live-attenuated Salmonella Typhi 21a vaccine might stimulate systemic and mucosal antibody responses in previously exposed lactating individuals. However, to enhance the trustworthiness and applicability of these findings, future research must have robust study designs with larger sample sizes, prioritizing the evaluation of safety for both mothers and their babies.

### 3.5. Influenza Vaccine (Flu)

#### 3.5.1. Background

Influenza, commonly referred to as ‘flu’, is an acute respiratory disease caused by an infection with the influenza virus. Symptoms range from asymptomatic to life-threatening, influenced by variables such as the type of virus and host factors, including age and existing health conditions. Pregnant women and young infants face higher risks of severe complications from influenza, although no vaccines are approved for infants under 6 months [96,97]. Vaccination during the second or third trimester of pregnancy helps to reduce influenza-related infant hospitalizations [98]. There are three primary types of influenza vaccines: live-attenuated (LAIV), inactivated (IIV), and recombinant vaccines. It is recommended that pregnant women receive the IIV during flu season, which typically ranges from October to May in the Northern Hemisphere and from April to September in the Southern Hemisphere [99,100]. Both IIV and LAIV are approved for use in breastfeeding women postpartum, though concerns about the use of live vaccines during breastfeeding persist [101].

#### 3.5.2. Included Studies

Two studies, incorporating a total of 264 lactating individuals, assessed influenza vaccination postpartum. The first, a randomized, double-blinded trial, evaluated the safety and effectiveness of postpartum influenza vaccination [21]. Participants in the first group received a live-attenuated influenza vaccine (LAIV) administered as a nasal spray, together with a subcutaneous injection of a placebo. The second group received an inactivated influenza vaccine (IIV) via subcutaneous injection, paired with a nasal spray placebo. This study assessed the safety of mothers and infants and evaluated their effectiveness using biomarkers, measuring serum and human milk antibodies. The second study, a cross-sectional inquiry, scrutinized the correlation between influenza vaccination and immune cell responses in human milk, encompassing T-cell surface markers and cytokine concentrations [22].

#### 3.5.3. Outcomes

**I. Safety in Lactating Individuals**: The findings from a large RCT (n = 248) indicate that both IIV (n = 124) and LAIV (n = 124) are generally well tolerated by lactating individuals. The majority involved local reactions such as pain at the injection site for IIV recipients (56%) and nasal congestion for LAIV recipients (45%). Importantly, no serious adverse events were recorded within the 6 months following vaccination [21].

**II. Safety in Infants**: Viral testing conducted on nasal swabs and human milk samples both pre-vaccination and 2 and 8 days post-vaccination—derived from 124 LAIV mother-infant pairs—predominantly showed no detectable vaccine-strain or wild-type influenza viruses. Two isolated cases (one infant and one mother) briefly tested positive for the influenza A vaccine strain in nasal swabs without any associated adverse events. Among 249 infants monitored for 10 days post-vaccination, those whose mothers received LAIV had higher incidences of irritability/fussiness compared to those whose mothers received IIV (59.7% versus 44.8%; *p* = 0.02). No significant AEs were reported in any of the 245 infants monitored for 6 months.

**III. Immunogenicity in Lactating Individuals**: The RCT (n = 248) demonstrated stronger systemic immune responses in women vaccinated with IIV compared to LAIV. The IIV group had significantly larger amounts of IgG antibodies in their serum, with the geometric mean titers (GMTs) for H1N1, H3N2, and B strains exceeding those of the LAIV group.

**IV. Infant Immunity Through Human Milk**: Furthermore, the RCT demonstrated that human milk from lactating women receiving IIV had significantly higher IgA and IgG levels, as well as overall higher milk conversion rates, compared to milk from LAIV recipients [21]. A separate cross-sectional analysis of 16 lactating women found elevated gene expression for the T-cell surface markers CD44 (*p* = 0.035), CD8A (*p* = 0.021), CD62L (*p* = 0.049), and CD25 (*p* = 0.007) in human milk from vaccinated participants compared to unvaccinated mothers. These markers suggest a possible association between influenza vaccination and T-cell activity in human milk [22].

V. Effectiveness in Lactating Individuals or Infants—Not studied.

#### 3.5.4. Quality of the Evidence

The evidence on postpartum influenza vaccination primarily comes from a single high-quality RCT employing rigorous methods, including double blinding, proper randomization, and comprehensive follow-up of 249 mother-infant pairs. As shown in Table 5, this robust methodology supports high certainty evidence for maternal safety, infant safety, and maternal immunogenicity outcomes.

The high certainty rating for maternal safety is further supported by the finding that adverse events in lactating individuals closely matched the established safety profile seen in general population vaccine trials [102,103]. This consistency strengthens confidence in the safety findings and suggests that lactation status might not significantly alter the expected safety profile of influenza vaccines.

While elevated antibody levels in human milk suggest potentially enhanced immunity in breastfed infants, this evidence is downgraded to moderate certainty due to indirectness, as it measures immunological markers rather than clinical protection. Additional research on infant clinical outcomes would strengthen these findings. Despite these limitations, the available evidence provides strong support for influenza vaccination in breastfeeding populations.

Although not directly studied in this postpartum context, a separate RCT examining in-pregnancy maternal influenza vaccination found increased anti-influenza IgA levels in human milk after birth and significantly reduced incidence of respiratory illness with fever in infants of vaccinated mothers [104]. These findings suggest similar protective effects may occur with postpartum vaccination, though more targeted research is needed to confirm this hypothesis.

While the current evidence strongly supports influenza vaccine use in breastfeeding populations, further research with direct infant health outcome measures remains essential to substantiate these recommendations and provide a more comprehensive understanding of clinical effectiveness.

#### 3.5.5. Conclusion

Overall, the chapter concludes that administering inactivated influenza vaccines parenterally to postpartum women is both safe and immunologically effective. There’s evidence to suggest that it offers protective benefits for breastfed infants. However, further high-quality studies are necessary to transform these findings into robust, generalized clinical recommendations.

### 3.6. Pertussis Vaccine (Whooping Cough)

#### 3.6.1. Background

Whooping cough is a highly contagious disease caused by the bacterium Bordetella pertussis, with potentially severe consequences for infants [105]. Vaccination against pertussis is recommended for children during routine immunization schedules and pregnant individuals at 22 weeks of gestation. Two types of vaccines are available: Tdap and DTaP, which both protect against tetanus, diphtheria, and pertussis. The ‘a’ in both vaccines signifies ‘acellular’, meaning the vaccine only contains parts of the Bordetella pertussis bacteria instead of the whole cell. The primary difference between these two vaccines lies in the quantity of diphtheria toxoid and pertussis antigen they contain; the Tdap vaccine contains substantially less than the DTaP vaccine. DTaP vaccines are given to infants and young children under 10 years of age, while the Tdap vaccine is administered during pregnancy or shortly after delivery.

#### 3.6.2. Included Studies

Three studies on postpartum pertussis vaccination were discovered. Two studies assessed the impact of the combined Tdap vaccine, given to lactating individuals, upon the levels of specific anti-pertussis antibodies found in human milk [49,50]. The effectiveness of the vaccine in reducing the risk of pertussis infection in infants was the subject of one case-control study [106]. However, none of these studies examined the safety-related outcomes of the pertussis vaccination or reported on the efficacy of vaccine biomarkers in lactating individuals.

#### 3.6.3. Outcomes

I. Safety in Lactating Individuals—Not studied.

II. Safety in Infants—Not studied.

**III. Immunogenicity in Lactating Individuals:** A comprehensive cohort study involving 234 lactating mothers observed higher anti-pertussis toxin IgA and IgG levels at delivery in mothers vaccinated during pregnancy compared to those who were unvaccinated during pregnancy. Over time, the variations in serum anti-pertussis toxoid (PT) IgG levels between mothers vaccinated during pregnancy and those vaccinated postpartum diminished, with no significant differences observed 4 weeks postpartum. Likewise, anti-PT IgA levels showed no significant differences from 8 weeks postpartum onwards. Furthermore, vaccinated mothers who gave birth prematurely displayed higher serum IgA levels between 4 and 8 weeks, while mothers who delivered at term showed higher IgG levels at 4 and 12 weeks postpartum [49].

**IV. Infant Immunity Through Human Milk**: Two observational studies examined antibody levels in human milk following maternal vaccination [49,50]. The first study discovered significantly higher levels of anti-PT SIgA antibodies in human milk from individuals vaccinated during pregnancy or shortly after delivery compared to unvaccinated controls [50]. The second study reported increased levels of anti-PT IgA and IgG in colostrum and throughout lactation in both term and preterm infants whose mothers received Tdap vaccination during pregnancy [49].

V.1 Effectiveness in Lactating Individuals—Not studied.

**V.2 Effectiveness in Infants:** A case-control study examined the effectiveness of postpartum parental pertussis vaccination in reducing pertussis infections in infants. When both parents were vaccinated after delivery, it reduced the risk of infant pertussis by 77%, exhibiting an adjusted vaccine effectiveness of 64% after accounting for confounding factors like maternal education and the presence of a sibling in the household [106]. Sole postpartum vaccination of the mother was associated with lower odds of pertussis infection in the infant, at 60% effectiveness. However, this protective effect was not retained after accounting for the same confounding factors.

#### 3.6.4. Quality of the Evidence

The quality assessment of the evidence on postpartum pertussis vaccination is summarized in Table 6, showing very low certainty across all outcome domains with available evidence.

The complete absence of safety data for both mothers and infants represents a critical research gap that should be addressed in future studies. The evidence supporting immunogenicity in lactating individuals is primarily limited by the absence of randomized studies and moderate risks of bias arising from unaddressed confounders and selection methods. The small sample sizes further restrict the precision and generalizability of findings. Evidence for immune responses in breastfed infants faces similar limitations as the immunogenicity evidence, with the additional concern of indirectness—the studies measure antibody levels rather than direct protection against disease. This creates significant uncertainty about the clinical relevance of the observed immunological changes. For vaccine effectiveness, the evidence quality is particularly low. While the single case-control study showed promising results when both parents were vaccinated (77% reduced risk), this evidence is severely undermined by wide confidence intervals and insufficient adjustment for confounding variables. This limitation is especially apparent when examining maternal-only vaccination, where the protective effect disappeared after controlling for confounders. The absence of additional corroborating studies further weakens confidence in these findings.

#### 3.6.5. Conclusion

These findings imply that postpartum maternal Tdap vaccination might enhance anti-pertussis antibodies in breast milk, thereby offering some protection to newborns before their first vaccinations. However, the evidence’s very low certainty underscores the necessity for more rigorous studies to thoroughly evaluate the efficacy and safety of postpartum vaccinations in lactating individuals.

### 3.7. Pneumococcal Vaccine

#### 3.7.1. Background

*Streptococcus pneumoniae* is a bacterium that causes various illnesses, including serious invasive infections like meningitis, pneumonia, and sepsis, as well as milder infections such as otitis and sinusitis. Young children under 2 years of age are particularly vulnerable and account for a significant majority of severe cases. More than 90 serotypes exist, but only a few are known to cause invasive pneumococcal diseases. The development of pneumococcal vaccines and their inclusion into national immunization programs have significantly reduced the burden of pneumococcal disease [107]. Available vaccines include pneumococcal polysaccharide vaccine (PPSV23, Pneumovax 23) and pneumococcal conjugate vaccines (PCV13, Prevnar 13 and PCV20, Prevnar 20). Childhood immunization typically involves a three-dose schedule of the PCV13 vaccine or a four-dose schedule regimen for the PCV20 vaccine during the first year of life, a single dose of PPSV23 for adults over 65 years of age, and high-risk groups depending on their medical conditions, either vaccine may be recommended [108].

#### 3.7.2. Included Studies

A single, non-randomized study examined the immune response in three lactating participants who had received the 23-valent polysaccharide vaccine. The primary focus was on secretory IgA levels in milk, with secondary emphasis placed on other types of antibodies in milk and serum [51]. We found no studies on vaccine safety for lactating mothers or their infants.

#### 3.7.3. Outcomes

I. Safety in Lactating Individuals—Not studied.

II. Safety in Infants—Not studied.

**III. Immunogenicity in Lactating Individuals:** The study noted inconsistent serum antibody responses post-vaccination, with differing IgG and IgA levels among individuals.

**IV. Infant Immunity Through Human Milk**: Notable increases were observed in pneumococcal-targeting sIgA concentrations in human milk. Additionally, neutrophils demonstrated the dose- and complement-dependent killing of S. pneumoniae due to sIgA antibodies in human milk, indicating these vaccine-induced sIgA antibodies in human milk initiate and mediate bacterial clearance.

V. Effectiveness in Lactating Individuals or Infants—Not studied.

#### 3.7.4. Quality of the Evidence

The quality of evidence for pneumococcal vaccination during lactation is rated as very low certainty across all measured outcomes, as summarized in Table 7.

This assessment stems from fundamental methodological limitations of the single available study. The extremely small sample size (n = 3) introduces severe imprecision, while the observational design without a control group significantly increases the risk of bias. For infant immunity outcomes, though the finding that vaccine-induced pneumococcal-targeting antibodies in human milk facilitate bacterial clearance might indicate a potential mechanism for infant protection, the evidence faces additional downgrading due to indirectness, as the study measured biomarkers rather than clinical protection. Important research gaps remain, with no evidence available regarding safety for either lactating individuals or their infants nor any data on clinical effectiveness.

#### 3.7.5. Conclusion

Overall, the findings from the reviewed study offer preliminary insights into the potential of the PPSV23 vaccine to enhance sIgA concentrations in human milk, which may benefit mothers and their infants. However, there is a lack of evidence regarding the clinical effectiveness of postpartum vaccination. Larger, randomized studies are needed to confirm these biomarker effects, assess their clinical impact, and evaluate the safety of the vaccine while breastfeeding. This will guide vaccination recommendations for lactating individuals.

### 3.8. Polio Vaccine

#### 3.8.1. Background

Poliovirus is an extremely infectious viral disease that predominantly affects children. It can result in paralysis and even, in severe cases, death. There is no cure for paralytic polio, nor can any treatment reverse the paralysis it induces. This fact underscores the essential nature of vaccination against poliovirus. Immunization strategies have markedly reduced the prevalence of paralysis and limb deformity around the world [109]. Two types of vaccine are available: the inactivated (or killed) poliovirus vaccine (IPV), administered via injection, and the live-attenuated poliovirus vaccine, which is ingested orally (OPV) [110].

#### 3.8.2. Included Studies

In this review, a single observational cohort study was included, assessing the effects of various polio vaccination strategies on antibody levels in serum and human milk of 40 lactating women [52]. Participants from different geographical regions with varying previous exposure to poliovirus antigens (either from childhood immunization or natural infection) were studied. The study compared the usage of parenteral polio vaccine (inactivated), oral polio vaccine (live-attenuated), and a mix of oral polio and parenteral cholera vaccines [52]. However, no studies were found addressing vaccine safety in lactating women or the direct safety effects on breastfed infants.

#### 3.8.3. Outcomes

I. Safety in Lactating Individuals—Not studied.

II. Safety in Infants—Not studied.

**III. Immunogenicity in Lactating Individuals**: The impact of polio vaccination on serum antibodies varied based on vaccine type and prior exposure history. In cohorts receiving parenteral inactivated vaccine, significant increases in serum IgG were observed two weeks post-vaccination, regardless of baseline levels (which were high in Swedish participants due to childhood vaccination and detectable but lower in Pakistani participants from endemic exposure). In contrast, participants receiving oral live-attenuated vaccine demonstrated a different pattern: serum IgA levels decreased three-fold post-vaccination, while IgG and IgM levels remained unchanged from their baseline endemic exposure levels.

**IV. Infant Immunity Through Human Milk**: Changes in the IgA antibody levels in human milk were compared across different vaccination strategies. Swedish participants did not have detectable antibodies in their milk pre-vaccination but showed a transient increase in milk IgA levels post-parenteral vaccination with a killed poliovirus vaccine. Participants from Pakistan had detectable IgA levels in their milk prior to vaccination, consistent with their natural exposure history. Pakistani participants receiving the parenteral-killed polio vaccine experienced significant rises in secretory IgA antibodies post-vaccination in nearly half of the cases. Conversely, Pakistani participants receiving the live oral polio vaccine, particularly if paired with the parenteral cholera vaccine, showed a notable decrease in their milk IgA post-vaccination.

V. Effectiveness in Lactating Individuals or Infants—Not studied.

#### 3.8.4. Quality of the Evidence

The rating for the evidence quality of polio vaccines is summarized in Table 8.

The evidence on polio vaccination during lactation comes from a single observational study with significant methodological limitations. The divergent immune responses observed between different population groups highlight the impact of prior exposure and vaccine type on both systemic and mucosal immunity. The contrasting responses pose intriguing questions about the interplay of vaccine types and mucosal immune regulation while underscoring the complexity of predicting passive immunity benefits for infants via maternal vaccination [111]. 

The observed variations in response patterns, coupled with the small sample size and non-randomized design, significantly limit the certainty of evidence. The study does not control for confounding factors such as time since delivery, frequency of breastfeeding, and other potential immunomodulatory conditions. Furthermore, without clinical outcome data, the immunological findings serve only as indirect markers of potential benefit, making it difficult to assess their clinical significance for infant protection.

#### 3.8.5. Conclusion

The study suggests that prior exposure and vaccine type influence both systemic and mucosal antibody responses, potentially affecting passive immunity in breastfed infants. Parenteral inactivated vaccines boosted while oral live vaccines temporarily reduced antibody levels. However, the clinical significance of these findings needs further clarification. Future research should prioritize RCTs with diverse populations that measure clinical outcomes, allowing for the development of evidence-based vaccination recommendations for lactating women from different geographical and immunological backgrounds.

### 3.9. Rabies Vaccine

#### 3.9.1. Background

Rabies is a deadly infectious disease caused by the rabies virus, which is typically transmitted to humans through animal bites or scratches. After infection, the virus targets nerve tissues and travels to the central nervous system, causing neurological symptoms such as hyperactivity, convulsions, paralysis, and, ultimately, death. Prevention through vaccination is crucial, with pre-exposure prophylaxis (PrEP) recommended for high-risk individuals like travelers or animal workers. PrEP involves two doses of an inactivated rabies vaccine. In the event of potential exposure, post-exposure prophylaxis (PEP) is vital and includes wound care, active immunization, and possibly human anti-rabies immunoglobulin (HRIG), depending on vaccination status. The implications of these protocols for lactating women warrant consideration [112].

#### 3.9.2. Included Studies

For this review, only one case report was identified concerning rabies vaccination in a lactating woman [53].

#### 3.9.3. Outcomes

I. Safety in Lactating Individuals—Not studied.

II. Safety in Infants—Not studied.

III. Immunogenicity in Lactating Individuals—Not studied.

IV. Infant Immunity Through Human Milk—Not studied.

**V. Effectiveness in Lactating Individuals or Infants:** This single case involved a breastfeeding 17-year-old woman who was bitten by a rabid fox. She experienced a delayed administration of HRIG, which could have caused the development of rabies. Despite her continuing to breastfeed, her infant, who was treated with the human diploid cell rabies vaccine (HDCV) and HRIG, remained free of symptoms. The report highlighted the severe consequences of inadequate rabies PEP [53].

#### 3.9.4. Quality of the Evidence

The evidence regarding rabies vaccination during lactation is extremely limited, consisting of only a single case report (Table 9).

#### 3.9.5. Conclusion

The limited data preclude substantive conclusions regarding the safety or efficacy of rabies vaccination during lactation. This gap necessitates additional studies, particularly larger, controlled, or observational studies, to robustly inform clinical guidelines. In the meantime, adhering to vaccination protocols in cases of potential rabies exposure is crucial to prevent infection and potential fatalities. It is also vital to enhance rabies awareness among healthcare providers and the public to ensure timely and effective prophylaxis.

### 3.10. Rotavirus Vaccine

#### 3.10.1. Background

Rotavirus predominantly infects children under 5, manifesting symptoms such as diarrhea, vomiting, fever, and dehydration. If treatment is not prompt, it can result in severe consequences. Before the introduction of vaccines in 2006, rotavirus was a primary cause of child mortality and hospitalizations worldwide. Several oral, live-attenuated vaccines are now available, designed to prevent severe rotavirus gastroenteritis. These vaccines have substantially reduced rotavirus-related hospitalizations and deaths. The WHO recommends incorporating rotavirus vaccines into national immunization programs, especially in high-fatality regions. Vaccination against rotavirus is advised for children, barring conditions such as severe immunodeficiency or a history of intussusception [113].

#### 3.10.2. Included Studies

The current comprehensive review incorporates data from a solitary study focused on postpartum rotavirus vaccination. This was an open RCT involving 32 participants who received either a rotavirus vaccine (n = 21) or a placebo (n = 11). Among those in the vaccination groups, 11 were administered the monovalent rotavirus vaccine, and ten received the tetravalent rotavirus vaccine [23].

#### 3.10.3. Outcomes

**I. Safety in Lactating Individuals**: Among the 32 lactating individuals in the RCT, six out of 21 (22%) participants receiving the vaccine reported mild gastrointestinal side effects, such as loose stools, without systemic reactions. One (9%) placebo participant reported similar gastrointestinal side effects.

Despite the small sample size, the findings suggest a safety profile in lactating individuals that aligns with reports from non-lactating populations. For instance, findings from a study examining a different rotavirus vaccine (pentavalent) in elderly non-lactating adults found similar rates of gastrointestinal AEs between the vaccine and placebo groups [114]. Although the sample size was small, the consistent findings across different populations suggest that common AEs are manageable and comparable.

**II. Safety in Infants**: A total of 60 stool samples were collected from 30 infants and tested for rotavirus antigen in the first week following maternal vaccination; all tests were negative. Concurrently, only one out of 85 stool samples (1%) collected from the 30 lactating mothers tested positive for the rotavirus antigen. Meanwhile, all 89 samples of human milk (from 32 lactating mothers) collected in the first 2 weeks post-vaccination tested negative for the rotavirus antigen. It is important to point out that AEs in infants were not evaluated, thus limiting the comprehensiveness of the safety assessment.

**III. Immunogenicity in Lactating Individuals**: Vaccine immunogenicity was demonstrated by an increase in serum IgG levels following vaccination, while serum IgG levels among placebo recipients remained unchanged over time. However, IgA levels in the serum remained consistent over time for both vaccine and placebo recipients.

**IV. Infant Immunity Through Human Milk**: Significant increases in SIgA titers were observed in the human milk of vaccinated participants during 4 months of follow-up.

V. Effectiveness in Lactating Individuals or Infants—Not studied.

#### 3.10.4. Quality of the Evidence

The quality assessment of evidence for the rotavirus vaccine is summarized in Table 10.

Although the study was well-conducted, with an overall low risk of bias, the sample size and loss of follow-up can impact interpretation. Furthermore, issues such as the lack of blinding and incomplete randomization reporting introduce some uncertainty regarding the results. The evidence for safety in infants was limited by the absence of an assessment for AEs in infants. Moreover, the study did not directly evaluate disease reduction, revealing a significant gap in understanding the vaccine’s impact on infant disease outcomes. Further research is necessary to validate these outcomes over a prolonged period and in larger populations, evaluate the clinical impact of these biomarkers and monitor safety for both maternal and infant health.

#### 3.10.5. Conclusion

In summary, findings from the review of the study indicate a generally positive safety profile, along with enhanced immunogenicity of human milk following maternal vaccination with an oral, live-attenuated rotavirus vaccine. However, the evidence is limited by the small size of the study and its restricted scope. Future research should concentrate on larger studies to evaluate effectiveness and public health impacts, particularly in providing passive immunity to infants who are contraindicated to receive the rotavirus vaccination.

### 3.11. Rubella Vaccine

#### 3.11.1. Background

Rubella, also known as German measles, is often an asymptomatic viral infection. When clinical disease does manifest, it is usually mild, with symptoms such as fever, facial rash, and painful swollen lymph nodes that generally resolve themselves within a month. However, the Rubella virus is notorious for its possible effects on unborn children when infection occurs during the first half of pregnancy [115]. The virus can impact multiple fetal organ systems, leading to congenital defects, a condition known as congenital rubella syndrome (CRS), which is one of the most common causes of vaccine-preventable birth defects. Therefore, although rubella vaccination is typically part of childhood immunization programs, immediate postpartum immunization is recommended for individuals with seronegative rubella status during pregnancy and no history of rubella vaccination [116]. Rubella vaccination is provided as a component of the combined measles, mumps, rubella (MMR) vaccine, which contains a live-attenuated rubella virus strain. Currently, most licensed vaccines are based on the RA 27/3 strain, TO-336 strain, or BRD-II strain [116].

#### 3.11.2. Included Studies

We reviewed seven studies, comprising one RCT, three observational cohort studies, and one cross-sectional study. These studies evaluated both the safety and efficacy (via biomarkers) of postpartum rubella vaccinations across a total of 1788 participants. In addition, two case reports highlighted potential rubella cases occurring shortly after vaccination. A variety of live-attenuated rubella vaccines were used in these studies, including the RA 27/3, HPV-77 DE-5, Cendehill, and TO-336 strains, and they were administered via different routes—both parenteral and intranasal.

#### 3.11.3. Outcomes

**I. Safety in Lactating Individuals**: An analysis of 1750 lactating women showed variability in AE profiles after rubella vaccination. In a cohort of 949 lactating participants, the HPV-77 DE-5 strain had the highest AE rate compared to the RA27/3 and Cendehill strains. Joint pain, considered the most burdensome reaction, was reported by 32% [25]. A RCT involving 546 participants reported that 30% of participants experienced joint complaints with the RA27/3 strain compared to 20% in the placebo group [24]. Another study with 254 participants documented minor AEs, such as temporary arthralgia and fever, occurring in 6% of cases after TO-336 strain vaccination [26].

In comparison, a retrospective study assessed the safety of the MMR vaccine among 9.9 million non-lactating adults and adolescents. This study revealed lower rates of non-serious side effects post-vaccination. Arthropathy was reported in approximately 0.23% of doses (230 per 100,000 doses), while fever occurred in around 0.07% (73 per 100,000 doses). Gender differences were observed, with males reporting higher rates of arthropathy and females experiencing more frequent fevers and rashes [117]. However, unlike the lactation studies, this investigation did not include lactating individuals, and the lactation studies excluded non-lactating counterparts, complicating direct comparisons. These variations in study populations, as well as the inherent physiological and hormonal differences in postpartum women, may contribute to the different incidence rates.

**II. Safety in Infants**: This synthesis evaluates the safety of infants, considering 152 infants ranging from 2 to 20 weeks post-maternal rubella vaccination. A study involving 63 infants found no clinical or serological evidence of rubella from 2 to 8 months post-vaccination, which indicates no adverse effects due to maternal immunization [25]. In another study, 26 infants were monitored over 20 weeks, and no clinical symptoms were found even though the virus was detected in 69% of maternal milk samples and 56% of nasopharyngeal secretions from breastfeeding mothers [28]. A smaller cohort identified viral shedding in 70% of milk and 77% of nasopharyngeal samples, affected by vaccine strain and route [29]. A prospective investigation observed viral shedding in milk and nasopharynx without any accompanying symptoms [27]. Although viral shedding is common, infants display no clinical symptoms, indicating a low adverse risk.

**III. Immunogenicity in Lactating Individuals:** Immunogenicity was assessed in three studies involving 1216 participants. In a Swedish cohort study with 949 participants, individuals vaccinated within 4 days postpartum exhibited a 96% seroconversion rate with the RA 27/3 strain [25]. In a study conducted in the United States involving 13 lactating women, the RA 27/3 strain was administered via subcutaneous and intranasal routes, or the HPV-77 DE5 strain, with all participants showing strong systemic and local immune responses [29]. In Japan, a study with 254 individuals receiving the TO-336 strain noted that 89% achieved a fourfold increase in HI titers, indicating effective immunization despite initially low titers [26].

**IV. Infant Immunity Through Human Milk**: The analysis of infant immunogenicity involves two studies with a total of 89 infants. A cross-sectional study of 63 infants found no serological evidence of rubella at 2 to 8 months post-vaccination [25]. A cohort study of 26 mother-infant pairs reported a 25% infant seroconversion rate, with a transient nasopharyngeal IgA presence [28]. Rubella-specific IgA presence in milk was confirmed in both studies, but the direct immune transfer to infants was not substantiated, indicating complexity in the passive immunity transfer [29]. Despite mixed findings, postpartum maternal vaccination appears to indirectly benefit infant immunogenicity.

V. Effectiveness in Lactating Individuals or Infants—Not studied.

#### 3.11.4. Quality of the Evidence

Current evidence moderately supports the safety and immunogenic potential of postpartum rubella vaccination despite the observation of viral shedding in up to 70% of cases. Importantly, no clinical symptoms were reported in infants, suggesting that the presence of viral components in human milk may not adversely affect infant health. The GRADE evidence summary (Table 11) provides a transparent assessment of these findings, with moderate certainty for both maternal safety and immunogenicity and low certainty for infant safety. While the immunogenic potential is promising in lactating individuals, the indirect benefits for infant immunity via breastfeeding remain unclear and are rated as very low certainty. The absence of direct studies on vaccine effectiveness underscores an important gap in future research.

#### 3.11.5. Conclusion

In summary, postpartum rubella vaccination seems to be safe for those who are lactating, presenting potential immunogenic advantages and showing no evident adverse effects on infants despite instances of maternal viral shedding being observed. More research is crucial to comprehensively understand the benefits of postpartum vaccination in mothers and to firm up clinical recommendations. Future investigations ought to assess clinical outcomes for both those who are lactating and their infants, as well as examine shedding dynamics and any potential immunity passed on through breastfeeding.

### 3.12. Varicella Vaccine (Chickenpox)

#### 3.12.1. Background

The Varicella zoster virus (VZV) causes chickenpox, a highly contagious infection that is transmitted through direct contact or respiratory routes. While typically mild in children, it presents with a fever and a characteristic progressive rash that evolves from macules to vesicles, then crusts. However, varicella can cause severe complications in pregnant women, neonates (especially if the mother contracts the infection within 5 days before or 2 days after delivery), immunocompromised individuals, and adults. Some complications include bacterial infections, pneumonia, and neurological manifestations. A primary infection usually provides lifelong immunity, but VZV remains dormant. Live-attenuated vaccines (Oka strain) are available as monovalent or MMR-combination formulations for individuals aged 12 months and above (and some from 9 months). This vaccine is administered either subcutaneously or intramuscularly [118]. Vaccination post-exposure in children and adults within 3 days after exposure has proven to be highly effective against moderate or severe disease [119].

#### 3.12.2. Included Studies

The available evidence on postpartum varicella vaccination stems from one observational follow-up study and two case reports [32,33]. The observational study involved a follow-up of 12 lactating individuals and their breastfed infants for 6 weeks after receiving the first and second vaccine doses. Participants were given two doses of a live-attenuated, parenterally-administered (Oka strain) vaccine. The first dose was administered at least 6 weeks postpartum, and the second dose was administered 4 weeks after the first dose [31].

#### 3.12.3. Outcomes

**I. Safety in Lactating Individuals**: An observational study, which included 12 lactating participants, monitored the occurrence of a rash following varicella vaccination. The results showed that only one participant reported a rash, a development determined via polymerase chain reaction (PCR) testing to be unrelated to the vaccination. There were two case reports of secondary varicella vaccine transmission to newborn infants following maternal postpartum vaccination. In the first, no side effects were noted by the vaccinated mother [33]. In contrast, the second case report presented an erythematous papular rash that was accompanied by crusted lesions. This rash, which tested positive for the Oka vaccine strain via PCR testing, developed 3 weeks after varicella vaccination [32].

**II. Safety in Infants**: Within a 6-week follow-up of 12 breastfed infants after their mothers received varicella vaccine doses, no vesicular rashes were reported in infants through observation. The PCR analysis of 217 human milk and infant serum samples found no varicella DNA, demonstrating a null risk of viral transmission from vaccinated mothers to infants. This lack of detectable DNA suggests that the risk of shedding—hence, transmission—is exceptionally low or non-existent under these circumstances [31]. Data from broader vaccine trials shows that a vaccine-induced rash has an incidence of just 1% after administering two doses to healthy adults [120]. Furthermore, viral shedding is uncommon in immune-competent individuals and typically only occurs when a rash is present [121].

However, isolated case reports necessitate attention. One report describes a breastfed infant who developed an extensive, disseminated vesicular rash along with MRI abnormalities 4 weeks after the mother received a postpartum varicella vaccination; both the infant and the mother tested positive for the vaccine-strain virus [32]. The second case discusses a newborn who developed a rash 3 weeks after maternal postpartum vaccination, and the mother did not develop a rash; the authors speculate on the potential transmission via respiratory secretions or saliva [33].

**III. Immunogenicity in Lactating Individuals**: All 12 lactating participants achieved seroconversion, as measured by varicella-specific IgG antibodies using an enzyme-linked immunosorbent assay (ELISA) after vaccination. This robust immunogenic response underscores the vaccine’s ability to induce an effective immune response in postpartum individuals.

**IV. Infant Immunity Through Human Milk**: Despite rigorous ELISA testing of infant serum samples, we detected no signs of immunity in any of the infants following maternal vaccination. This finding was potentially affected by sample availability; only half of the intended samples were available for analysis [31]. A case of secondary varicella vaccine transmission in a breastfed infant led to detectable levels of varicella-specific IgG and immunoglobulin M (IgM) antibodies in the infant’s serum [32].

V. Effectiveness in Lactating Individuals or Infants—Not studied.

#### 3.12.4. Quality of the Evidence

The GRADE evidence summary, presented in Table 12, highlights the limitations in the current evidence base for varicella vaccination during lactation.

The observational data suggests that varicella vaccination is generally safe for use in healthy breastfeeding women postpartum, while the case reports indicate the potential for rare transmission events. Although no viral shedding was detected in the observational study, the small sample size and a non-randomized study design limit the detection of rare AEs and the generalizability of these findings. While the evidence leans towards a minimal transmission risk in the absence of a rash, caution remains warranted. We recommend recently vaccinated caretakers of infants under 6 months who have a vesicular rash to seek advice from a healthcare professional.

#### 3.12.5. Conclusion

In summary, while the evidence substantiates the safety of postpartum varicella vaccinations for mothers and indicates a minimal transmission risk to infants, there are still gaps in our knowledge. There’s a need for extensive, randomized trials to comprehensively assess the vaccine’s effectiveness, the infant’s immune response, and any rare AEs stemming from vaccination. These efforts are crucial in shaping trustworthy guidelines and grasping the vaccine’s impact on maternal and child health.

### 3.13. Variola Virus Vaccine (Smallpox)

#### 3.13.1. Background

Smallpox, caused by the Variola virus, was historically one of humanity’s most devastating diseases, characterized by fever and a distinctive progressive skin rash. The disease was particularly severe in pregnant women and infants, with mortality rates reaching 30% in unvaccinated populations. The successful global eradication of smallpox, declared by the WHO in 1980, stands as one of the greatest achievements in public health history. This was achieved through systematic vaccination campaigns using Vaccinia virus-based vaccines. Although routine vaccination ceased after eradication, orthopoxvirus vaccines remain relevant due to bioterrorism concerns and the emergence of related viruses, such as Monkeypox. Current vaccines are available in three generations: first-generation (animal-derived), second-generation (cell-culture-derived), and third-generation (attenuated or non-replicating). Guidelines recommend non-replicating vaccines (such as MVA-BN) for breastfeeding persons and contraindicate replicating vaccines (such as ACAM2000). However, these recommendations are largely based on theoretical considerations rather than empirical evidence [122,123,124].

#### 3.13.2. Included Studies

The empirical evidence regarding the risks of vaccination in breastfeeding contexts is limited to a single-case report published in the Journal of the American Medical Association (JAMA) in 2004. This report describes a chain of vaccinia transmission following a military smallpox vaccination with ACAM2000, a second-generation replicating vaccinia virus vaccine.

#### 3.13.3. Outcomes

I. Safety in Lactating Individuals—Not studied.

II. Safety in Infants: The single case report documents tertiary vaccinia transmission despite stringent adherence to standard precautions. Transmission occurred from a vaccinated soldier to his breastfeeding wife, who developed vesicular lesions on both areolas and consequently to their infant daughter, who developed facial and oral lesions. Laboratory confirmation via PCR and culture testing validated vaccinia infection in both the mother and the infant. While both recuperated without specific treatment, the healing was prolonged due to complications related to moisture [34].

The strength of the case report lies in its detailed documentation, laboratory confirmation, and thorough follow-up. However, being a single-case report, it provides limited evidence for establishing causation or determining transmission rates. The exact mechanism of initial transmission from the vaccine to the mother remains uncertain despite reported adherence to standard precautions, underlining the challenges in preventing household spread.

III. Immunogenicity in Lactating Individuals—Not studied.

IV. Infant Immunity Through Human Milk—Not studied.

V. Effectiveness in Lactating Individuals or Infants—Not studied.

#### 3.13.4. Quality of the Evidence

The GRADE evidence summary (Table 13) highlights the extremely limited nature of the available evidence, with infant safety receiving a very low certainty rating based on a single case report. The lack of evidence across all other outcome domains represents a significant gap in knowledge regarding vaccination safety and efficacy in lactating populations.

#### 3.13.5. Conclusion

This case report provides evidence of the risk for tertiary contact vaccinia with second-generation replicating vaccines (such as ACAM2000), consistent with current guidelines that caution against their use in breastfeeding contexts. Current guidelines recommend non-replicating, third-generation vaccines (such as MVA-BN) in lactating individuals. However, it is important to note that no direct evidence was retrieved to support the safety of these vaccines in lactating individuals, though their safety has been established in other vulnerable study populations [124]. The lack of evidence across all outcome domains underscores the necessity for more systematic research on the use of pox vaccines in breastfeeding environments. This is particularly crucial given the renewed significance of orthopoxvirus vaccines in response to bioterrorism threats and emerging diseases such as monkeypox.

### 3.14. Yellow Fever Vaccine

#### 3.14.1. Background

Yellow fever (YF) is a mosquito-borne viral disease caused by the yellow fever virus, a Flavivirus, endemic in 44 countries across sub-Saharan Africa and South America [125]. YF disease presents with fever, muscle pain, and headache, potentially progressing to severe symptoms including jaundice, bleeding, and organ failure in 15% of cases, with a 20–50% mortality rate in severe cases. As there are no specific treatments, prevention is paramount. Prevention can be achieved through immunization with live-attenuated vaccines derived from the 17D lineage, which are administered as a single 0.5 mL dose either subcutaneously or intramuscularly. The vaccine confers lifelong immunity in most recipients. While generally safe, contraindications to the vaccine include age < 6 months, severe immunosuppression, and severe egg allergies. For pregnant and lactating women, vaccination is recommended when the benefits outweigh the risks, especially in endemic areas or during outbreaks [126].

#### 3.14.2. Included Studies

In this review, we evaluated the existing evidence on YF vaccination in lactating women and their breastfed infants. Six studies were included in total, comprising two cohort studies, one case series, and three case reports. The studies primarily focused on the safety of postpartum vaccination for breastfed infants.

#### 3.14.3. Outcomes

**I. Safety in Lactating Individuals:** The safety data on YF vaccination in lactating women is primarily sourced from one cohort study and several case reports. Within the cohort study, 11,279 participants were included, out of which a single lactating woman was identified. She did not report any adverse effects after breastfeeding once, 2 days after vaccination with the 17D-204 live-attenuated strain [40]. In case reports concerning the 17DD strain, mild maternal side effects like transient headaches and malaise were observed [35,36].

**II. Safety in Infants:** Safety data for infants breastfed by vaccinated mothers come from diverse study designs. A single cohort study reported no AEs or the detection of viral ribonucleic acid (RNA) in the maternal serum or milk among 11 vaccinated mothers [38]. Conversely, a case series involving eight symptomatic infants found viral shedding in 6 (75%) of the mothers. It was detected in the serum and milk, with clearance observed between 11 and 24 days post-vaccination. This series specifically included symptomatic cases, thereby introducing potential selection bias [39].

Notably, individual case reports associated postpartum vaccination with severe infantile outcomes like meningoencephalitis. Still, the viral transmission routes remain speculative due to the absence of viral testing in milk [35,36,37]. The heterogeneity in the definitions of AEs, the timing, and methodologies across studies complicate drawing consistent conclusions about safety.

**III. Immunogenicity in Lactating Individuals:** One cohort study involving 11 participants found that only 9% showed Yellow Fever-specific IgM antibodies up to 2 weeks post-vaccination [38]. Meanwhile, an earlier-described case series that focused on symptomatic infants recorded that 63% of mothers had specific IgM antibodies [39]. Both studies, however, did not have baseline IgM levels, making it difficult to differentiate between prior exposure and recent vaccination effects.

**IV. Infant Immunity Through Human Milk:** Case reports provide limited evidence on immune responses in infants, noting Yellow Fever-specific IgM antibodies in hospitalized infants with meningoencephalitis [35,36,37]. However, the strength of this evidence is diminished due to biases inherent in such reports.

V. Effectiveness in Lactating Individuals or Infants—Not studied.

#### 3.14.4. Quality of the Evidence

The methodological quality of available studies is critically low, as illustrated in Table 14.

The evidence is consistently rated as very low certainty across all measured outcomes, primarily due to serious methodological limitations, including high risk of bias, inconsistent study designs, selection bias in case reports, and inadequate baseline measurements. These limitations underscore the need for cautious clinical application of the findings. When vaccination against yellow fever is deemed necessary, clinicians must carefully balance potential benefits against the concerning, though limited, evidence of serious adverse effects in breastfed infants.

#### 3.14.5. Conclusion

The narrative synthesis reveals significant evidence gaps in relation to the postpartum administration of the YF vaccine. In clinical practice where vaccination is indispensable, breastfeeding should be temporarily interrupted for a span of 2 to 3 weeks post-vaccination. This should be accompanied by the introduction of alternative feeding methods and infant monitoring. Going forward, future research ought to concentrate on solid cohort studies with standardized protocols to better understand the safety and efficacy of the vaccination in lactating women and their breastfed infants.

### 3.15. COVID-19 Vaccine (SARS-CoV-2)

#### 3.15.1. Background

The severe acute respiratory syndrome coronavirus 2 (SARS-CoV-2) emerged in late 2019, leading to a global pandemic known as the coronavirus disease 2019 (COVID-19). As SARS-CoV-2 spread rapidly, it presented unprecedented public health challenges worldwide, necessitating effective preventive measures and the urgent development of vaccines to curb its spread. Various types of vaccines have since been developed and introduced to the market, including messenger RNA (mRNA) and viral vector vaccines. However, due to the exclusion of pregnant and lactating women from initial vaccine trials, there has been an even greater interest in understanding the safety and efficacy of these vaccines in this demographic. Since the onset of the pandemic, COVID-19 vaccines have been extensively researched and discussed in lactating subjects [127,128], and this review will therefore present only a summary of these findings.

#### 3.15.2. Included Studies

In this comprehensive review, we analyzed a total of 41 articles to summarize the available data on COVID-19 vaccines administered to lactating individuals in the postpartum period. We included 36 prospective cohort studies, with a total of 19,376 participants, one cross-sectional study, two case reports, and two case series. Study characteristics are displayed in Table 1 and Supplementary Table S3 and summarized in the subsequent paragraphs. Several types of COVID-19 vaccines are described in the literature; these include mRNA-based (39 studies, n = 19,420), adenovirus-vectored (10 studies, n = 17,883), and inactivated whole-virus (1 study, n = 3) vaccines. Four of the five outcomes of interest for this review were described across studies on COVID-19 vaccines: maternal vaccine safety (22 studies, n = 18,584), safety for the breastfed infant (14 studies, n = 18,220), immunogenicity in lactating individuals (24 studies, n = 1273), and human milk or infant immunogenicity (34 studies, n = 1619).

#### 3.15.3. Outcomes

**I. Safety in Lactating Individuals:** COVID-19 vaccines in lactating individuals were generally well tolerated, with common side effects mirroring those seen in the general population. Local reactions such as pain at the injection site (91%) and systemic symptoms like fatigue (31%) were most frequently reported, with more pronounced effects following the second dose. A large cohort study involving 17,525 women, including 6815 lactating participants, found no significant differences in side effect profiles among pregnant, lactating, and non-pregnant/non-lactating individuals [63]. The finding that there are no significant differences in side effect profiles among pregnant, lactating, and non-pregnant individuals was corroborated in several other studies [56,58,66].

Serious adverse events were notably absent across multiple studies [54,56,65,66,69].

Some studies observed higher frequencies of symptoms with the mRNA-1273 (Moderna) vaccine compared to the BNT162b2 (BNT162b2-BioNTech) vaccine after the second dose [54]. Vector-based vaccines tended to cause more systemic symptoms like fever and headache compared to mRNA vaccines [69]. Isolated reports of more serious conditions, such as subacute thyroiditis, were retrieved, suggesting the need for further investigation into rare AEs [41,60].

Lactation-specific adverse events were uncommon, with mastitis reported in 3.4%, axillary lymphadenopathy in 5.7%, and breast engorgement in 1.1% of participants in one study (n = 88) [66].

Regarding milk supply, seven studies assessed the impact of vaccination on breast milk supply [54,57,63,65,66,70,71]. While most participants observed no alterations in the quantity of human milk produced post-vaccination, a small percentage (5–15%) reported temporary decreases that typically resolved within 72 h without intervention. For instance, a large-scale cohort study reported a 5% observation of reduced milk supply after the first dose and a 7.2% observation after the second dose. However, this reduction was temporary and typically lasted less than a day [63]. One study found that mRNA-1273 recipients more frequently experienced reduced milk supply compared to BNT162b2 recipients after the second dose (23.4% vs. 8.0%). All participants returned to normal milk production within 72 h without any interventions [54]. Similarly, in a third study, 6% of participants reported a decrease in milk production post-vaccination [70], while this percentage was 15% in a different study [71]. In contrast, other studies noted an absence of noticeable changes in milk supply among their participants after vaccination [65,66].

**II. Safety in Infants:** Investigations into maternal COVID-19 vaccination’s impact on breastfed infants focused on both the potential transfer of vaccine components into human milk (5 studies, 201 participants) and adverse reactions in infants (12 studies, 18,136 participants). Across multiple studies, detection of vaccine-related components in human milk was minimal or non-existent, with one study finding low levels of vaccine mRNA (<10%) in some samples, but most studies reporting no detectable levels of vaccine mRNA in human milk [57,65,72,74]. Importantly, in the few cases where vaccine mRNA was present in milk, it was not detected in the infant’s serum, possibly due to gastrointestinal digestion before absorption into the infant’s bloodstream [66,73].

Adverse effects in infants were generally mild and transient. A large cohort study involving 6815 lactating individuals reported that 3 to 4.4% of mothers expressed concerns about their infants post-vaccination, though the specific nature of these concerns was not specified. Importantly, no serious AEs were recorded [63]. In another study (n = 180), the most common symptoms were irritability (~10%) and poor sleep (~8%), with no serious AEs reported [54]. Several smaller studies reported similar findings, primarily noting concerns related to minor behavioral changes or symptoms such as increased tearfulness or sleeplessness [57,61,67]. One case report documented a cutaneous adverse reaction in an infant, characterized by erythematous tender plaques that presented 2 days post-vaccination of the mother [42].

**III. Immunogenicity in Lactating Individuals**: Research consistently shows substantial increases in SARS-CoV-2-specific IgG and IgA antibodies in serum post-vaccination [57,58,61,69,77,78,79,80,83]. Several studies found significantly greater neutralizing titers, higher natural killer (NK) cell activity, and robust T-cell response in vaccinated lactating individuals compared to their non-vaccinated (non-pregnant) controls [57,58,76,77,81]. Post-vaccination serum IgG levels were significantly higher than those in previously infected lactating individuals, with more consistent responses and higher neutralization titers [56,58,62,79,86].

Multiple studies reported that the second dose of mRNA vaccines significantly enhanced IgG and IgA levels, peaking 7–14 days post-second dose [56,58,83,86]. A third dose or booster vaccination further increased specific IgG levels and neutralizing activity [59,78]. Both mRNA vaccines yielded robust antibody levels, with mRNA-1273 inducing slightly higher IgA levels compared to BNT162b2 [56,58]. Studies also found that mRNA vaccines generated higher and more consistent IgG levels than vector-based vaccines like Oxford/AstraZeneca and Johnson & Johnson [56,58,82].

**IV. Infant Immunity Through Human Milk**: Thirty-three studies investigated COVID-19 vaccination during lactation and its potential protective effects for breastfed infants, a measure divided into subcategories: antibodies in human milk (32 studies); human milk neutralization (12 studies); immune cells in human milk (2 studies); and infant immune responses (5 studies).

COVID-19 vaccination during lactation consistently induced SARS-CoV-2-specific antibodies in human milk, demonstrated by 32 studies [55,56,57,58,59,61,64,65,67,69,70,71,72,74,75,76,77,78,79,80,81,82,83,84,85,86,87,89,91,92,129,130]. Both IgA and IgG antibodies are typically detected around one week following vaccine administration [57,71,72,81,82,83,87]. A biphasic pattern is often observed, with IgA levels peaking after each dose and IgG levels progressively increasing after the first and second doses [65,78,80,83]. mRNA vaccines generally induce higher IgA and IgG levels compared to adenovirus-based vaccines [69,82,92,129]. Antibody levels remain elevated up to 6 months post-vaccination, though they gradually decrease over that period [64,70,130]. Factors influencing antibody levels include the infant’s age, parity number, and duration of breastfeeding [55,89,91]. Vaccination typically results in an IgG-dominant response, while natural infection results in an IgA-dominant response [74,79,86].

Studies have also confirmed cross-reactivity with SARS-CoV-2 variants and the presence of neutralizing antibodies in human milk [72,84,85,87,89,130]. Twelve studies assessed the presence and dynamics of neutralizing antibodies in human milk after COVID-19 vaccination [56,64,67,70,72,74,78,81,84,85,88,130]. The majority of these studies demonstrated an enhanced neutralizing capacity of human milk against SARS-CoV-2 after an mRNA-based vaccine, with further improvement following a third dose (booster vaccination) [68,78,84]. In contrast, an observational cross-sectional study found that despite the presence of spike-specific IgG and IgA antibodies in human milk from vaccinated participants 10 days after the second dose, the neutralizing activity was similar to pre-pandemic samples [81]. Another observational study found variations for different vaccine types, for example, among mRNA-based BNT162b2 and vector-based Ad26.COV2.S vaccine recipients, 73% and 65% of milk samples showed neutralizing activity. In contrast, among recombinant Ad5-nCoV-S (CanSino) vaccine recipients, a significantly lower neutralization rate of 14% was observed, similar to the rates among non-vaccinated individuals [88].

Two studies highlighted SARS-CoV-2-specific immune cell responses in breastmilk following COVID-19 vaccination [76,81]; both reported spike-reactive T cells in human milk post-vaccination. The first study identified a unique T-cell population in human milk, with increased frequencies of effector and central memory T cells and noted expanded SARS-CoV-2-specific T-cell receptors after a third mRNA vaccine dose [76]. A subsequent study found early production of anti-spike antibodies, particularly SIgA, in most samples by day 10 post-first dose, increasing SIgA frequency from 70% to 87% after the second dose [81]. These findings suggest that vaccination during lactation can modulate immune cell populations in breast milk, which can be transferred to the infant through breastfeeding.

In addition to human milk antibodies, five papers explored the transfer of maternal immune response to the breastfeeding infant following maternal COVID-19 vaccination [57,67,70,75,85]. Three studies examined infant stool samples, consistently detecting SARS-CoV-2-specific antibodies [67,70,75]. One study, which included stool from 24 infants aged between 55 days and 11 months, found RBD-targeting IgG and IgA in 33% and 30% of infant stool samples, respectively, 3 weeks after the mothers completed a two-dose vaccination schedule with an mRNA vaccine [67]. A second study observed detectable levels of specific IgG and IgA in the stool from 24 infants (average age of 10 months) 6 months after maternal vaccination, with a significant increase in IgG levels compared to negative controls and enhanced neutralization activity in 50% of samples [70]. A third study demonstrated that human milk-derived anti-SARS-CoV-2 IgG and IgA significantly decreased after in-vitro infant gastrointestinal digestion but remained at detectable levels, unlike IgG antibodies originating from infected mothers’ milk, which deteriorated to undetectable levels [75].

Two studies examining antibodies in infant blood found no detectable antibodies in the infants’ blood following maternal vaccination despite high IgG levels in maternal serum and milk [57,85]. One study noted that saliva from three out of five (60%) breastfed infants contained SARS-CoV-2 specific IgG despite the absence of detectable antibodies in dried blood spots collected from 21 infants [85].

**V. Effectiveness in Lactating Individuals or Infants**—Not studied.

#### 3.15.4. Quality of the Evidence

This review employs a narrative synthesis to suggest that the COVID-19 vaccination in lactating women is generally safe and induces an immune response similar to that in the general population. The GRADE evidence summary (Table 15) provides a transparent assessment of the certainty of evidence across outcomes, with most findings based on observational studies.

Safety data for both mothers and infants is rated as very low certainty due to reliance on self-reported adverse events and potential biases from unvalidated measures. Evidence for maternal immunogenicity maintains a low certainty rating due to consistent findings across multiple studies with different populations and vaccine types. While promising antibody transfer in human milk suggests potential benefits for breastfed infants, this evidence is rated as very low certainty due to indirectness, as the clinical significance of these antibodies remains undetermined. Notably, no studies currently assess vaccine effectiveness through direct clinical outcomes, such as reduced infection or hospitalization rates in lactating individuals and their nursing infants.

#### 3.15.5. Conclusion

In summary, COVID-19 vaccination in lactating individuals is safe and elicits a strong immune response similar to those seen in the general population. There is low certainty of evidence supporting maternal safety and immunogenicity, but the certainty of evidence for infant safety and immunity is very low due to biases and indirectness issues. Overall, these findings support the continued use of COVID-19 mRNA-based vaccines in lactating women and underscore the need for further research to evaluate long-term vaccine effectiveness and its implications for infant health.

#### 3.15.6. Broader Implications for Vaccine Research

The methodologies and findings from this review of postpartum COVID-19 vaccinations can inform future research on various vaccines in lactating populations. By standardizing AE reporting, evaluating immunological protection through human milk, and analyzing vaccine-induced antibody profiles, similar frameworks can be applied to vaccines for other infectious diseases. This approach will enhance safety and efficacy assessments, support improved health recommendations, and could potentially boost vaccine acceptance among lactating individuals. Such research can contribute to the development of comprehensive guidelines that protect the health of both mothers and their breastfeeding infants.

## 4. Discussion

The goal of this review was to examine the impact of postpartum vaccination on maternal and infant health. To achieve this, we structured our literature analysis into four sub-questions, each addressing a specific area of the research question.

### 4.1. Effects on Maternal Health

#### 4.1.1. Safety

Our study identified that the availability of vaccine safety data in postpartum individuals is limited to COVID-19, rubella, influenza, rotavirus, and varicella vaccines. The reviewed literature indicates that severe adverse reactions are not common, and the side effects experienced by lactating individuals are generally consistent with the vaccines’ safety profiles observed in trials of the general population. These effects can be predicted based on general principles of vaccine reactogenicity, with key influencing factors such as vaccine type and administration route [17]. This suggests broadly that postpartum vaccination does not uniquely alter the safety profile of these vaccines for lactating individuals.

Among the vaccines reviewed, the rubella vaccine stands out due to some evidence suggesting a slightly increased incidence of musculoskeletal symptoms such as arthritis and arthralgia in postpartum women [24]. A meta-analysis supports this observation by highlighting that rubella vaccines commonly cause mild, acute, transient arthralgia or arthritis in adult females (10–25%) [131]. A natural infection with the wild-type rubella virus is also known to cause arthritis and arthralgia more frequently in adult women than in children and men. Importantly, vaccine-associated joint symptoms are less frequent, less severe, and of shorter duration than those following a natural rubella infection, where up to 70% of women experience persistent joint issues [116]. Therefore, while the rubella vaccine can induce joint symptoms, it remains a favorable choice compared to the potentially severe and long-lasting effects of the natural infection.

Overall, this review reinforces the safety of vaccinating lactating women. It highlights the need for further research, particularly on vaccines with limited existing data. This is to strengthen the prevailing evidence base and support well-informed vaccine recommendations for lactating individuals.

#### 4.1.2. Immunogenicity

The vaccines reviewed against COVID-19, Rubella virus, *Vibrio cholerae* [44,47], Influenza virus [21,22], *Bordetella pertussis* [49,50], *Streptococcus pneumoniae* [51], and Rotavirus [23], have demonstrated significant immunogenic responses, similar to those observed in vaccine trials on broader populations [132,133]. Vaccines for rabies, smallpox, and varicella zoster, however, remain understudied in lactating women. Safety and efficacy outcomes of vaccines are traditionally determined through controlled clinical trials that assess both safety and immune responses. However, these trials often exclude lactating individuals, leading to a knowledge gap. Studies indicate an altered immune status in postpartum individuals due to hormonal and metabolic changes during pregnancy that do not immediately return to their non-pregnant baseline [134]. Interactions between hormonal, metabolic, and immune responses might potentially influence vaccine immunogenicity [135]. Overall, the immunogenicity profiles of the reviewed vaccines resembled those achieved in the general population, and despite potential physiological alterations in the postpartum period, there are no clear indications of significant differences from the expected vaccine-induced responses.

### 4.2. Effects on Infant Health

#### 4.2.1. Safety

An examination of infant safety data post-maternal postpartum vaccination reveals vital findings on various vaccines. Well-researched vaccines like mRNA-based COVID-19 and inactivated influenza vaccines appear to be safe for infants postpartum. Conversely, vaccines like smallpox, YF, varicella, and rubella are associated with potential risks, suggesting a need for more cautious administration and well-informed decision-making. Table 16 provides an overview of studies assessing vaccine shedding post-postpartum vaccination with a live-attenuated vaccine. Additionally, there exist vaccines with limited or no research, such as cholera, pertussis, pneumococcal, and polio vaccines, making it challenging to make definitive statements about their safety.

Postpartum rubella vaccination is generally deemed safe for breastfed infants. Although the vaccine virus might be present in human milk and potentially transmitted to the infant, such occurrences are infrequent and typically result in only mild symptoms [27,28,29]. These findings underscore the necessity of informing mothers about possible mild reactions and urging them to consult with healthcare providers if concerns surface. This supports the well-being of both the mother and child after vaccination.

Considering the YF vaccine, the review emphasizes the potential risks of viral transmission to infants after maternal vaccination [36,39]. YF viral shedding in human milk can occur up to 23 days following vaccination [39]. Moreover, three case reports have associated maternal postpartum YF vaccination with adverse neurological events in breastfed infants [35,36,37], but only one case was confirmed to be caused by the YF vaccine virus strain (YEL-AND). However, the exact transmission route was not established [36]. Despite a potentially low likelihood of these events, the severity of their possible consequences for infants demands caution. On the other hand, YF carries a high mortality rate, and prevention through immunization remains crucial [125,126,136]. Healthcare providers are advised to assist parents in balancing the preventive benefits of vaccination against the theoretical and uncertain risks of virus transmission. In situations where traveling to or residing in a yellow fever-endemic area is necessary and vaccination cannot be postponed, one might consider a temporary interruption of breastfeeding for up to 3 weeks post-vaccination.

Regarding the varicella vaccine, findings from a non-randomized study demonstrated neither viral shedding in human milk nor the occurrence of rash events in infants over 4 months [31]. However, two isolated case reports suggested a, albeit rare, possibility of viral shedding and transmission [32,33]. These findings align with broader vaccine trial data suggesting that varicella vaccine viral transmission is extremely rare in healthy vaccinated individuals. Since its extensive deployment in vaccination campaigns beginning in 1995, there have been only a few reports of vaccine virus transmission [121]. The small sample of postpartum women significantly limited the ability to capture rare AEs. Overall, these findings suggest that varicella vaccination in healthy breastfeeding women is beneficial. It aids in preventing wild-type VZV transmission to the infant—who is at an increased risk for severe illness—while presenting minimal risk of mild secondary vaccine transmission. Thus, while direct data on postpartum varicella vaccination is scarce, the benefits of vaccination seem to outweigh the potential risks.

The smallpox vaccine presents unique considerations stemming from both its historical context and its connection to recent developments in mpox vaccines. Traditional smallpox vaccines, which contain the live Vaccinia strain as seen in the ACAM2000 vaccine, appear to pose a significant risk of tertiary contact Vaccinia when used in households with newborn infants [34]. Infections occur through contact with the vaccination site; hence, the risk of infection is similar for breastfed and bottle-fed infants. As of now, no evidence of transmission through human milk has been found [122]. Thus, these findings suggest a cautionary approach to the use of the smallpox vaccine, especially in households with infants. However, updated mpox vaccines utilize a modified Vaccinia strain that does not replicate in human cells, thereby reducing associated risks. Yet even though severe AEs are theoretically less likely with these vaccines, no evidence regarding their use postpartum is available, so we cannot offer definitive recommendations [137].

In conclusion, while certain vaccines given during lactation, such as mRNA-based COVID-19 and inactivated influenza vaccines, appear safe for infants, others, including the YF vaccine, highlight the importance of comprehensive risk-benefit analysis and educated parental guidance.

#### 4.2.2. Infant Immunity Derived from Human Milk

##### Human Milk Immune Response

The immune response present in human milk exhibits a consistent pattern following maternal postpartum vaccination across various vaccines, displaying characteristic temporal and dynamic antibody excretion patterns. Generally, IgA responses appear early (between 2–4 weeks), while IgG responses develop at later stages or following booster doses. The administration route impacts the nature of the response. Transmucosal vaccines, for instance, tend to favor a mucosal IgA response, whereas parenteral vaccines trigger IgG-dominant immune responses. Inactivated vaccines, notably COVID-19 vaccines, demonstrate the most consistent response patterns. Conversely, the efficacy of bacterial vaccines often hinges on prior exposure. Response magnitude and duration differ depending on the type of vaccine, with some maintaining sustained antibody levels for multiple months post-vaccination. Despite being indirect, evidence on vaccine-induced human milk immunogenicity indicates a potential beneficial effect for breastfed infants. This potential benefit is by conferring passive immunity through antibodies that serve as a primary defensive mechanism at the mucosal level.

##### Infant Immune Response

Evidence of the infant immune response following maternal postpartum vaccination shows distinct patterns based on the vaccine type. Live-attenuated vaccines and the subsequent viral shedding and transmission have been recognized numerous times. With rubella vaccines, this risk of transmission was accompanied by the potential for seroconversion in the breastfed infant, but without severe clinical consequences for the infant [27,28,30]. Infants did not display systemic immune responses following maternal vaccination with inactivated vaccines [57,72,85]. However, there was evidence for mucosal immune responses as specific antibodies were detected in stool and saliva [67,70,85]. This concept aligns with the general idea that maternal antibodies provide passive immunity to the infant by serving as the first line of defense at mucosal surfaces [3,138]. Interestingly, bacterial vaccines lack any data on infant immune responses. In conclusion, the evidence base for maternal postpartum vaccination’s impact on infant immunogenicity reveals significant gaps in the systematic documentation of infant responses across all vaccine types, making definitive comparisons challenging.

#### 4.2.3. Effectiveness

Clinical outcome measures for assessing vaccine effectiveness in infants were limited across the studied vaccines. Only evidence on the associations between pertussis and cholera vaccines and their protective effects against diseases in infants was available [48,106].

### 4.3. Contextual Considerations: Vaccine Hesitancy

While vaccine hesitancy was not a primary outcome measure in our review, it represents a critical contextual factor that influences the real-world impact of the evidence we have synthesized. We include this discussion to help healthcare providers translate our findings into clinical practice, recognizing that evidence alone is insufficient without strategies to address implementation barriers. The following insights on vaccine hesitancy are drawn from external literature rather than from our primary analysis.

The extent to which vaccines are effective is not only related to vaccine factors such as their immunogenicity profiles but also depends on factors such as local vaccine availability, vaccine confidence, and acceptance within the population. The rise of social media platforms has increased the spread of misinformation about vaccination, which has, in turn, been shown to increase hesitancy towards vaccination [139,140]. Because healthcare providers play a crucial role in boosting vaccine confidence by accurately informing individuals about vaccination and addressing concerns to minimize hesitancy, this section will touch briefly on this topic.

Vaccine hesitancy, which is defined as a reluctance or refusal to vaccinate despite the availability of vaccines, has been identified by the World Health Organization as one of the top ten global health threats (WHO) [141]. This issue is especially prevalent among pregnant and lactating women, which leads to lower vaccine uptake within these populations [142,143].

Several systematic reviews have identified factors that contribute to vaccine hesitancy in pregnant and lactating individuals [4,142,144]. Primary concerns include safety for both mother and infant, swift vaccine development and approval processes that may imply insufficient testing, and underrepresentation in clinical trials. Moreover, using social media as a primary information source has been associated with lower vaccination rates [142]. A lack of healthcare provider recommendations and misconceptions about the necessity of vaccines were also noted as significant barriers to vaccination during pregnancy and the postpartum period [144].

Several strategies have shown promise in improving vaccination rates. The approach to communicating with vaccine-hesitant individuals depends on where the discussion is taking place; it could be public or private. In public discussions, the guidance document provided by the World Health Organization is a useful tool for countering anti-vax arguments [145]. Private discussions between healthcare providers and patients mainly depend on personal relations. Healthcare professionals play a crucial role in influencing vaccination decisions; recommendations from a trusted provider are a key factor in vaccine acceptance [144]. It is crucial to address safety concerns and provide accurate information about vaccines to improve uptake among pregnant and lactating individuals [144]. Furthermore, clear communication regarding the reactogenicity profile of a vaccine can help manage expectations and boost vaccine confidence [17].

Furthermore, as parental decisions are influenced by their own experiences with vaccines [146], addressing vaccine hesitancy in pregnant and lactating women could potentially improve vaccine uptake among children. This could make significant strides in enhancing public health overall. Strategies such as integrating vaccination into routine antenatal care and providing consistent, clear communication about vaccine benefits and safety have been identified as effective approaches to increase vaccination rates in this population [144].

### 4.4. Limitations

This review possesses notable limitations that require meticulous consideration when interpreting its results. The evidence base was primarily limited by the commonality of small sample sizes and dependence on observational designs, which could potentially introduce selection bias and limit generalizability. The variation in study designs and the absence of a unified methodological framework complicated direct comparisons among studies.

A fundamental limitation presented itself in the form of heterogeneity in outcome measurements and reporting across different studies. This was particularly noticeable in the inconsistent methods and timing utilized for antibody measurement, the variation in safety outcome definitions and reporting, non-standardized and often deficient follow-up periods for infant outcomes, and the diverse approaches to documenting breastfeeding practices.

The review exposed significant gaps in vital outcome domains, particularly the scarce availability of long-term follow-up data for both mothers and infants, standardized measurements of mucosal and T-cell immunity, and effectiveness data associating immunological responses with clinical protection. The absence of standardization in sample collection, assay selection, and data analysis emerged as a key obstacle across studies, especially impacting the interpretation of immunological outcomes.

Publication bias might have influenced the findings, primarily with respect to safety data, since adverse effects tend to be addressed less rigorously than beneficial outcomes and may be underreported in the literature. It was challenging to quantify this potential bias, given the diverse nature of the selected studies and the absence of standardized reporting structures for AEs in lactating populations.

### 4.5. Recommendations

Upon reviewing the data, the following recommendations can be made for vaccination during lactation (for a comprehensive overview with evidence quality assessments, see Table 2):

- Inactivated vaccines (COVID-19, cholera, influenza, pertussis, pneumococcus, poliovirus, and rotavirus) are generally safe and effective for lactating individuals and their infants. These vaccines can be administered without the need for breastfeeding interruption.
- YF vaccine: postponing vaccination is preferred; however, if vaccination is deemed necessary, breastfeeding interruption should be recommended for at least 3 weeks after vaccination due to the risk of virus transmission and potentially serious adverse consequences for the infant. Advice should be provided on maintaining milk supply and infant feeding.
- Rubella vaccine: Potential transmission of rubella virus to infants through breastmilk is suggested from observational studies, although usually without clinical consequences for the infant. Clear guidance on adverse reactions lactating participants might experience is important for expectation management.
- Rabies vaccine: Given the severe consequences of rabies infection, rabies vaccination is justified in lactating women when necessary. Healthcare providers should ensure timely vaccination and provide guidance on breastfeeding, balancing its benefits against potential risks.
- Smallpox vaccine: traditional smallpox vaccine, ACAM2000, in household members of young infants requires advice on precautionary measures to prevent tertiary contact vaccinia; advising lactating women to avoid close contact with recently vaccinated individuals against either smallpox or Mpox is appropriate.
- The varicella vaccine appears to be safe to use in lactating women without the need for breastfeeding interruption, though advice should be given that if a vesicular rash occurs following vaccination, they might consult with their healthcare provider about whether precautionary measures are needed.

### 4.6. Implications and Future Research

This comprehensive review addresses a crucial gap in the vaccination literature by providing the first structured consolidation of evidence on postpartum vaccination across multiple vaccine types. The prior fragmentation of evidence and circular referencing between guidelines obscured the true evidence base, creating uncertainty for healthcare providers. Our systematic analysis using consistent evaluation criteria clearly delineates evidence quality, enabling informed clinical decision-making despite knowledge gaps. Our findings bear several significant implications for clinical practice and public health policy:

- Healthcare providers can now access consolidated evidence to guide vaccination decisions for lactating individuals
- Offers a framework for discussing benefits and risks with patients
- Findings support the general safety of most vaccines during lactation, helping address vaccine hesitancy
- Highlights the need to include lactating individuals in vaccine trials, particularly during public health emergencies
- Demonstrates the importance of standardized safety monitoring

Future research priorities:

1. Well-designed clinical trials, specifically including lactating individuals
2. Standardized approaches to measuring vaccine responses in maternal serum, human milk, and infants
3. Long-term follow-up studies of infant outcomes
4. Investigation of vaccines against emerging infectious diseases (e.g., Mpox) in lactating populations
5. Studies examining the correlation between mucosal immune factors and clinical protection

## 5. Conclusions

This review offers the most thorough analysis to date concerning vaccine safety and efficacy in lactating individuals. By replacing fragmented guidance and circular referencing with direct evidence assessment, we provide clarification where recommendations have previously lacked transparency about their evidence basis. Despite certain limitations, the review acts as a valuable tool for healthcare providers when counseling lactating parents about vaccination decisions. Our results endorse the compatibility of most vaccines with lactation and underscore potential benefits for the mother and infant. While most vaccines seem safe during lactation, there persists a need for more rigorous, standardized research, especially during public health crises. Healthcare practitioners can use these findings to promote informed discussions about vaccination during lactation, balancing both individual and public health benefits against potential risks. As new vaccines surface and public health challenges shift, this evidence foundation will necessitate regular updates to ensure optimal protection for both lactating people and their infants.

## Figures and Tables

**Table 1:** Studies included for each vaccine and outcome measure.

Vaccine (No. Studies)	Safety			Immunogenicity			
	Adverse Reactions Mother	Vaccine Shedding	Adverse Reactions Infant	Maternal Serum	Maternal Human Milk	Infant Sample	Effectiveness for Infants
Influenza (2)	RCT [21]	RCT [21]	RCT [21]	RCT [21]	RCT [21] NRS [22]	No data	No data
Rotavirus (1)	RCT [23]	RCT [23]	No data	RCT [23]	RCT [23]	No data	No data
Rubella (7)	RCT [24] NRS [25,26] CR [27]	NRS [28,29] CR [27,30]	NRS [25,28] CR [30]	NRS [25,26,29] CR [27,30]	NRS [28,29] CR [27]	NRS [25,28] CR [27,30]	No data
Varicella (3)	NRS [31] CR [32,33]	NRS [31] CR [32,33]	NRS [31] CR [32,33]	NRS [31]	No data	NRS [31]	No data
Smallpox (1)	No data	CR [34]	CR [34]	No data	No data	No data	No data
Yellow fever (6)	CR [35,36,37]	NRS [38] CS [39] CR [35,36]	NRS [38] CS [39] CR [35,36,37,40]	NRS [38] CS [39]	No data	CR [35,36,37]	No data
COVID-19 (41)	NRS ^A^ CR [41]	NRS ^B^	NRS ^C^ CR [42]	NRS ^D^	NRS ^E^	NRS ^F^	No data
Cholera (6)	No data	No data	No data	RCT [43,44] NRS [45,46,47]	RCT [43,44] NRS [45,46,47]	No data	NRS [48]
Pertussis (3)	No data	No data	No data	NRS [49]	NRS [49,50]	No data	NRS [51]
Pneumococcal (1)	No data	No data	No data	NRS [51]	NRS [51]	No data	No data
Polio (1)	No data	No data	No data	NRS [52]	NRS [52]	No data	No data
Typhoid (2)	No data	No data	No data	RCT [44] NRS [45]	RCT [44] NRS [45]	No data	No data
Rabies (1)	No data	No data	No data	CR [53]	No data	No data	CR [53]

RCT = randomized-controlled study; NRS = non-randomized study; CR = case report; CS = case series. Green indicates evidence of at least one observational or experimental study; light grey indicates evidence is based on case reports only; dark grey indicates no studies retrieved. Safety refers to different concerns regarding the administration of vaccines to postpartum breastfeeding individuals. Vaccine shedding is defined as the excretion of vaccine components in human milk or other maternal secretions, such as nasal secretions. Immunogenicity is defined as the capacity of a vaccine to stimulate an immune response in the recipient. References for COVID-19 vaccine studies corresponding with letters in superscript in table: A [54,55,56,57,58,59,60,61,62,63,64,65,66,67,68,69,70,71,72], B [57,65,72,73,74], C [54,55,61,63,65,66,67,69,75], D [55,56,57,58,59,61,64,67,68,69,70,71,72,76,77,78,79,80,81,82,83,84,85,86], E [55,56,58,59,61,62,63,64,65,67,68,69,70,71,72,74,75,76,77,78,79,80,81,82,83,84,85,86,87,88,89,90,91,92], F [57,67,70,72,75,85].

**Table 2:** Summary of evidence and recommendations for the administration of vaccines to breastfeeding mothers.

Vaccine	Recommendation		Summary of Findings (GRADE)	
	Breastfeeding|Considerations		Lactating Mothers	Infants
**Cholera**	**✓**	No special considerations.	⊕ Effective immune response with oral vaccine [High], parenteral vaccine [Moderate]. ⊖ Safety data for postpartum use are unavailable.	⊕ Increased HM SIgA levels [Moderate, oral vaccine; Very Low, parenteral], Risk of severe cholera in infants reduced [Low], oral vaccine. ⊖ Safety data are unavailable.
**COVID-19**	**✓**	Prefer mRNA vaccines due to a more favorable/stable immune response.	⊕ Effective immune response with mRNA-vaccines [Low], variable with vectored vaccines [Very Low]. ⊖ Mild AE profile, consistent with the general population [Very Low].	⊕ Increased HM immune response (nAbs and T-cells) with mRNA vaccines [Low], variable with vectored vaccines [Very Low]. Antibodies detected in the stool of breastfed infants with mRNA vaccines [Very Low]. ⊖ Mild and transient AEs, no SAEs [Very Low]. Minimal to no vaccine-mRNA excretion [Very Low], no clinical symptoms in infants noted.
**Influenza**	**✓**	Prefer IIV due to a more favorable immunogenicity and safety profile.	⊕ Effective immune response with IIV > LAIV [High]. ⊖ Mild AE profile, consistent with the general population [High].	⊕ Increased HM nAbs [Moderate] and T-cell activity [Very Low]. ⊖ Mild and transient AEs (LAIV > IIV) [High]. Shedding was detected in maternal nasal secretions but not in HM; no transmission to infants was observed [High]. No clinical symptoms were noted.
**Pertussis**	**✓**	No special considerations.	⊕ Effective immune response [Very Low]. ⊖ Safety data for postpartum use are unavailable	⊕ Increased HM IgA levels [Very Low]. Reduced risk of pertussis infection in infants [Very Low]. ⊖ Safety data are unavailable.
**Pneumo-coccal**	**✓**	No special considerations.	⊕ Variable immune response [Very Low]. ⊖ Safety data for postpartum use are unavailable.	⊕ Increased HM SIgA and nAbs levels [Very Low]. ⊖ Safety data are unavailable.
**Polio**	**✓**	Prefer IPV due to a more favorable/stable immune response.	⊕ Effective immune response with IPV [Very Low]. Ineffective with OPV in infection-primed individuals [Very Low]. ⊖ Safety data for postpartum use are unavailable	⊕ Increased HM SIgA with IPV but decreased with OPV [Very Low]. ⊖ Safety data are unavailable.
**Rabies**	**✓** ✎	Given the high mortality of rabies, timely vaccination is justified when warranted.	⊕ Efficacy data for postpartum use are unavailable. ⊖ Infection (single case) despite PEP treatment, possibly due to inadequate timing and administration [Very Low].	⊕ Infant (single case) remained free of infection despite continued breastfeeding after maternal viral exposure and maternal PEP treatment [Very Low]. ⊖ Safety data not highlighted.
**Rotavirus**	**✓**	No special considerations and lack of clinical relevance: vaccine indication for infants, not for adult use.	⊕ Effective immune response [Moderate]. ⊖ Mild gastrointestinal symptoms [Moderate].	⊕ Increased HM SIgA levels observed [Moderate]. ⊖ No shedding detected; no transmission to infant [Moderate]. No symptoms were reported.
**Rubella**	**✓** ✎	Consider that while HM shedding is possible, no clinical consequences have been observed; manage expectations for potential side effects. ^a^	⊕ Effective immune response [Moderate]. ⊖ Mild, transient joint pain is a common AE [Moderate].	⊕ Increased HM IgA [Low]. Seroconversion observed in breastfed but not bottle-fed infants [Very Low]. ⊖ Viral shedding in HM is possible, but no clinical symptoms in infants despite shedding [Low].
**Smallpox**	✘ ✎	Given the risk of contact vaccinia, advice on precautionary measures to prevent infant exposure; also, for prevention of smallpox/Mpox, consider newer generation vaccines over ACAM2000. ^b^	⊕ Efficacy data for postpartum use are unavailable. ⊖ Safety data for postpartum use are unavailable.	⊕ HM, or infant immune response data, are unavailable. ⊖ A single case of transmission through contact with vesicular rash; viral shedding in rash vesicles. [Very Low]
**Typhoid**	**✓**	No special considerations.	⊕ Effective immune response with oral vaccine in primed- individuals [Low]. ⊖ Safety data for postpartum use are unavailable.	⊕ Increased HM SIgA levels observed [Very Low]. ⊖ Safety data are unavailable.
**Varicella**	**✓** ✎	Safe to use without breastfeeding interruption; advise caution and medical guidance if vesicular rash occurs post-vaccination.	⊕ Effective immune response [Low]. ⊖ Vaccine-related rash, though occurrence is rare [Very Low].	⊕ No serum antibodies detected in uninfected infants [Very Low] ⊖ Viral shedding through maternal rash vesicles; isolated neonatal varicella cases with variable severity [Very Low].
**Yellow fever**	✘ ✎	If possible, postpone. Otherwise, consider breastfeeding interruption for ≥ 3 weeks and provide guidance on maintaining milk supply and infant feeding.	⊕ Variable immune response 2 weeks post-vaccination [Very Low]. ⊖ Safety data for postpartum use are unavailable.	⊕ HM or infant immune response data are unavailable. ⊖ Shedding in HM, viral detection up to 23 days post-vaccination [Very Low]. Several reports on severe vaccine-related AEs [Very Low].

Summary of evidence and recommendations for the administration of vaccines to breastfeeding mothers. This table provides insights into whether particular vaccines are advisable during breastfeeding by synthesizing existing data on immunogenicity and safety for both lactating mothers and their infants. Recommendations are based on weighing the benefits and risks of vaccination against natural infection and comparison with the established literature. Recommendation Symbols: [**✓**] Green check: Vaccine recommended if indicated; [**✓**✎] Green check with pencil: Recommended with side notes for consideration; [✘✎] Red cross with pencil: Generally, not recommended unless high-risk infection scenarios; consult healthcare provider with considerations; [**✓**] Grey check: Indicates dubious clinical relevance for the recommendation on rotavirus vaccines. Findings Symbols: [⊕] Immunogenicity and potential benefits; [⊖] Safety concerns and adverse events. Notes: “Shedding” refers to the appearance of vaccine components in maternal samples; “Transmission” captures the detection of vaccine components in infant samples; Levels of evidence are based on GRADE principles, and assessments are expressed as the level of certainty and placed in brackets. Comprehensive quality assessments of the included studies can be found in Supplementary Tables S1 and S2. Annotations: ^a^ natural infection with wild-type rubella virus causes joint-related symptoms more frequently in adult women than in children and male adults. Rubella vaccine-related joint symptoms are also more frequent in adult women but are less frequent, severe, and of shorter duration than those from natural infection, making the vaccination preferable to facing the severe and long-lasting effects of wild-type virus infection. ^b^ Newer generation Smallpox and Mpox vaccines show promising safety profiles for their use in vulnerable populations as they contain a non-replicating vaccine strain [93], in contrast to the second-generation vaccines (such as ACAM2000) that contain a replicating vaccinia strain. Abbreviations: mRNA (messenger ribonucleic acid), HM (human milk), p.o. (oral), i.m. (intramuscular), s.c. (subcutaneous), SIgA (secretory immunoglobulin A), nAbs (neutralizing antibodies), (S)AE (severe adverse events), PEP (post-exposure prophylaxis), IIV (inactivated influenza vaccine (intramuscular injection)), LAIV (live-attenuated influenza vaccine (nasal spray)), IPV (inactivated polio vaccine (subcutaneous injection)), OPV (oral polio vaccine (live-attenuated virus)).

**Table 3:** GRADE evidence summary—Cholera vaccines.

Outcome	Study Design	Starting Level	Down/Upgrading Domains	Final Certainty
Safety in Lactating Individuals	No studies	N/A	N/A	No evidence
Safety in Infants	No studies	N/A	N/A	No evidence
Immunogenicity (Oral Vaccines)	RCTs	High	None	High
Immunogenicity (Parenteral Vaccines)	Observational	Low	Risk of bias (+1)	Moderate
Infant Immunity (Oral Vaccines)	RCTs	High	Indirectness (−1)	Moderate
Infant Immunity (Parenteral Vaccines)	Observational	Low	Indirectness (−1), Inconsistency (−1)	Very Low
Effectiveness in Infants	Observational	Low	Imprecision (−1), Indirectness (+1)	Low

Parenteral cholera vaccines upgraded from low to moderate due to a consistent dose-response relationship despite small samples. Infant immunity (oral) was downgraded for indirectness as outcomes did not measure immunity in the infant. Parenteral vaccines were further downgraded for inconsistency across different antibody measurements. Effectiveness was downgraded for imprecision (marginal statistical significance) despite robust adjustment for confounding variables. Abbreviations: N/A, not applicable.

**Table 4:** GRADE evidence summary—Typhoid vaccines.

Outcome	Study Design	Starting Level	Down/Upgrading Domains	Final Certainty
Safety in Lactating Individuals	No studies	N/A	N/A	No evidence
Safety in Infants	No studies	N/A	N/A	No evidence
Immunogenicity in Lactating Individuals	RCT + Observational	High	Risk of bias (−1), Imprecision (−1)	Low
Infant Immunity Through Human Milk	Observational	Low	Risk of bias (−1), Indirectness (−1), Imprecision (−1)	Very Low
Effectiveness	No studies	N/A	N/A	No evidence

Evidence on the Immunogenicity of Typhoid vaccines was downgraded due to small samples limiting precision and significant methodological limitations. Infant immunity evidence was further downgraded due to the use of indirect markers rather than clinical outcomes and high variability in immune response reporting.

**Table 5:** GRADE evidence summary—Influenza vaccines.

Outcome	Study Design	Starting Level	Down/Upgrading Domains	Final Certainty
Safety in Lactating Individuals	RCT	High	None	High
Safety in Infants	RCT	High	None	High
Immunogenicity in Lactating Individuals	RCT	High	None	High
Infant Immunity Through Human Milk	RCT	High	Indirectness (−1)	Moderate
T-cell Response in Human Milk	Cross-sectional	Low	Risk of bias (−1), Imprecision (−1)	Very Low
Effectiveness	No studies	N/A	N/A	No evidence

Safety and immunogenicity evidence maintained high certainty based on robust RCT design with appropriate blinding, randomization, and comprehensive follow-up. Infant immunity through milk downgraded one level for indirectness as outcomes measured antibody levels rather than disease prevention. T-cell response evidence was downgraded to very low due to cross-sectional design, small sample size (n = 16), and lack of adjustment for confounding factors.

**Table 6:** GRADE evidence summary—Pertussis vaccines.

Outcome	Study Design	Starting Level	Down/Upgrading Domains	Final Certainty
Safety in Lactating Individuals	No studies	N/A	N/A	No evidence
Safety in Infants	No studies	N/A	N/A	No evidence
Immunogenicity in Lactating Individuals	Observational	Low	Risk of bias (−1)	Very Low
Infant Immunity Through Human Milk	Observational	Low	Risk of bias (−1), Indirectness (−1)	Very Low
Effectiveness in Infants	Case-control	Low	Risk of bias (−1), Imprecision (−1)	Very Low

GRADE assessment of evidence quality for pertussis vaccination during lactation. All evidence came from observational studies (starting with low certainty). Immunogenicity evidence was downgraded due to the risk of bias from unaddressed confounders and selection methods. Infant immunity evidence was further downgraded for indirectness of outcome measures. Effectiveness evidence was severely limited by bias in the single case-control study, wide confidence intervals, and inadequate control for confounding factors when examining maternal-only vaccination.

**Table 7:** GRADE evidence summary—Pneumococcal vaccines.

Outcome	Study Design	Starting Level	Down/Upgrading Domains	Final Certainty
Safety in Lactating Individuals	No studies	N/A	N/A	No evidence
Safety in Infants	No studies	N/A	N/A	No evidence
Immunogenicity in Lactating Individuals	Observational	Low	Risk of bias (−1), Imprecision (−1)	Very Low
Infant Immunity Through Human Milk	Observational	Low	Risk of bias (−1), Imprecision (−1), Indirectness (−1)	Very Low
Effectiveness	No studies	N/A	N/A	No evidence

All evidence stems from a single non-randomized study with an extremely small sample size (n = 3). Evidence downgraded to very low due to serious risk of bias (no control group), severe imprecision, and, for infant immunity, additional concerns regarding indirect measures rather than clinical outcomes.

**Table 8:** GRADE evidence summary—Polio vaccines.

Outcome	Study Design	Starting Level	Down/Upgrading Domains	Final Certainty
Safety in Lactating Individuals	No studies	N/A	N/A	No evidence
Safety in Infants	No studies	N/A	N/A	No evidence
Immunogenicity in Lactating Individuals	No studies	N/A	Risk of bias (−1), Imprecision (−1), Indirectness (−1)	Very Low
Infant Immunity Through Human Milk	Observational	Low	Risk of bias (−1), Imprecision (−1), Indirectness (−1)	Very Low
Effectiveness	No studies	N/A	N/A	No evidence

Evidence from a single observational study (n = 40) was downgraded due to non-randomized design, small sample size, lack of controls for confounding, indirect outcome measures, and heterogeneous responses between population groups. Heterogeneous responses between geographical cohorts and vaccine types further complicate the interpretation and generalizability of findings.

**Table 9:** GRADE evidence summary—Rabies vaccine.

Outcome	Study Design	Starting Level	Down/Upgrading Domains	Final Certainty
Safety in Lactating Individuals	No studies	N/A	N/A	No evidence
Safety in Infants	No studies	N/A	N/A	No evidence
Immunogenicity in Lactating Individuals	No studies	N/A	N/A	No evidence
Infant Immunity Through Human Milk	No studies	N/A	N/A	No evidence
Effectiveness	Case report	Low	Risk of bias (−1), Imprecision (−1), Indirectness (−1)	Very Low

Effectiveness evidence based solely on a single case report, downgraded to very low certainty due to lack of comparative data, absence of systematic investigation, and inability to establish causality or generalizability.

**Table 10:** GRADE evidence summary—Rotavirus vaccines.

Outcome	Study Design	Starting Level	Down/Upgrading Domains	Final Certainty
Safety in Lactating Individuals	RCT	High	Risk of bias (−1), Imprecision (−1)	Moderate
Safety in Infants	RCT	High	Risk of bias (−1), Indirectness (−1)	Moderate
Immunogenicity in Lactating Individuals	RCT	High	Risk of bias (−1), Imprecision (−1)	Moderate
Infant Immunity Through Human Milk	RCT	High	Risk of bias (−1), Indirectness (−1)	Moderate
Effectiveness	No studies	N/A	N/A	No evidence

Evidence derived from single open RCT (n = 32). All outcomes were downgraded from high to moderate due to limitations in blinding and a small sample size. Infant safety and immunity were further downgraded for indirectness as infant adverse events were not directly evaluated, and clinical protection was not assessed.

**Table 11:** GRADE evidence summary—Rubella vaccines.

Outcome	Study Design	Starting Level	Down/Upgrading Domains	Final Certainty
Safety in Lactating Individuals	RCT + Observational	High	Risk of bias (−1), Inconsistency (−1)	Moderate
Safety in Infants	Observational	Low	Inconsistency (−1), Large effect (+1)	Low
Immunogenicity in Lactating Individuals	RCT + Observational	High	Risk of bias (−1), Inconsistency (−1)	Moderate
Infant Immunity Through Human Milk	Observational	Low	Risk of bias (−1), Inconsistency (−1)	Very Low
Effectiveness	No studies	N/A	N/A	No evidence

Safety and immunogenicity evidence was downgraded due to methodological concerns, including selection bias and inconsistent reporting across studies. Infant safety was maintained at low certainty despite observational design due to a large volume of consistent data showing a lack of clinical symptoms despite viral shedding. Infant immunity was downgraded to very low due to contradictory findings and complex factors affecting passive immunity transfer.

**Table 12:** GRADE evidence summary—Varicella vaccines.

Outcome	Study Design	Starting Level	Down/Upgrading Domains	Final Certainty
Safety in Lactating Individuals	Observational	Low	Imprecision (−1)	Very Low
Safety in Infants	Observational + Case reports	Low	Imprecision (−1), Inconsistency (−1)	Very Low
Immunogenicity in Lactating Individuals	Observational	Low	None (consistent findings)	Low
Infant Immunity Through Human Milk	Observational	Low	Risk of bias (−1), Imprecision (−1)	Very Low
Effectiveness	No studies	N/A	N/A	No evidence

The evidence stems from one observational study (n = 12) and two case reports. Safety was downgraded due to a small sample limiting the detection of rare events. Maternal immunogenicity is maintained at low certainty due to consistent seroconversion findings. Infant immunity is severely limited by missing data (only 50% of the intended samples were analyzed) and the inability to establish clinical significance.

**Table 13:** GRADE evidence summary—Smallpox vaccines.

Outcome	Study Design	Starting Level	Down/Upgrading Domains	Final Certainty
Safety in Lactating Individuals	No studies	N/A	N/A	No evidence
Safety in Infants	Case report	Low	Risk of bias (−1), Imprecision (−1)	Very Low
Immunogenicity in Lactating Individuals	No studies	N/A	N/A	No evidence
Infant Immunity Through Human Milk	No studies	N/A	N/A	No evidence
Effectiveness	No studies	N/A	N/A	No evidence

Infant safety evidence based solely on a single case report of tertiary transmission was downgraded to very low certainty due to the inability to determine transmission rates or generalize findings to broader populations.

**Table 14:** GRADE evidence summary—Yellow Fever vaccines.

Outcome	Study Design	Starting Level	Down/Upgrading Domains	Final Certainty
Safety in Lactating Individuals	Observational + Case reports	Low	Risk of bias (−1), Imprecision (−1)	Very Low
Safety in Infants	Observational + Case series	Low	Risk of bias (−1), Inconsistency (−1)	Very Low
Immunogenicity in Lactating Individuals	Observational	Low	Risk of bias (−1), Imprecision (−1)	Very Low
Infant Immunity Through Human Milk	Case reports	Low	Risk of bias (−1), Imprecision (−1), Indirectness (−1)	Very Low
Effectiveness	No studies	N/A	N/A	No evidence

All evidence was downgraded to very low certainty due to significant methodological limitations. Safety data is particularly limited by selection bias in case series (only including symptomatic infants), inconsistent reporting methods, and potential underreporting. Immunogenicity evidence is hampered by a lack of baseline measurements and small samples with inconsistent follow-up.

**Table 15:** GRADE evidence summary—COVID-19 vaccines.

Outcome	Study Design	Starting Level	Down/Upgrading Domains	Final Certainty
Safety in Lactating Individuals	Observational	Low	Risk of bias (−1)	Very Low
Safety in Infants	Observational	Low	Risk of bias (−1)	Very Low
Immunogenicity in Lactating Individuals	Observational	Low	None (consistent findings across multiple studies)	Low
Infant Immunity Through Human Milk	Observational	Low	Risk of bias (−1), Indirectness (−1)	Very Low
Effectiveness	No studies	N/A	N/A	No evidence

Evidence based on multiple observational studies. Safety evidence was downgraded due to reliance on self-reported data and potential recall bias. Maternal immunogenicity was maintained at low certainty due to consistent findings across multiple studies with different populations and vaccine types. Infant immunity was downgraded for indirectness as the clinical significance of detected antibodies remains undetermined.

**Table 16:** Viral shedding after postpartum vaccination with a live-attenuated vaccine.

Vaccine	Study Design [ref]	Population (Group)	Mothers (n/N)	Infants (n/N)	Viral Detection (+/Total)			Vaccine Strain Confirmed	Infant Serology	Infant Symptoms
					Mother, HM	Mother, Other	Infant, Sample			
**Yellow fever**	NRS [38]	Vaccinated LM	10/11	0	0/28	0/30	NT	-	NT	0/11
	CS [39]	Symptomatic infants	8	8	11/42	2/8	NT	NT	0/8	YF-like illness
	CR [35]	Symptomatic infant	1	1	NT	NT	0/1	-	Pos.	YEL-AND-like illness
	CR [36]	Symptomatic infant	1	1	NT	NT	1/2	Infant CSF	Pos.	YEL-AND
	CR [37]	Symptomatic infant	1	1	NT	NT	NT	NT	Pos.	YEL-AND like illness
**Rubella**	NRS [25]	LM	949	63	NT	NT	NT	-	Neg.	None
	NRS [29]	LM (vaccine 1)	4	0	3/4	4/4	NT	NT	NT	NR
		LM (vaccine 2)	4	0	4/4	4/4	NT	NT	NT	NR
		LM (vaccine 3)	5	0	2/5	2/5	NT	NT	NT	NR
	NRS [28]	LM (breastfeeding)	16	16	11/16	9/16	9/16	NT	4/16	0/16
		LM (non-breastfeeding)	10	10	NT	5/10	0/10	NT	0/10	0/10
	CR [27]	Symptomatic mother	1	1	1/3	0/1	1/2	NT	Pos.	None
	CR [30]	Symptomatic infant	1	1	0/1	0/1	0/3	-	Pos.	Rubella-like symptoms
**Varicella**	NRS [31]	LM	12	12	0/217	0/1	0/6	-	0/12	0/12
	CR [33]	Symptomatic infant	1	1	NT	NT	1/1	Infant rash	NT	Mild varicella disease
	CR [32]	Symptomatic infant	1	1	NT	1/1	3/3	Infant CSF, infant rash, maternal rash	Pos.	Extensive varicella disease
**Influenza**	RCT [21]	LM (live vaccine)	120/124	120/124	0/359	71/359	1/359	Maternal nasal swabs	NT	None
		LM (inactive vaccine)	31/124	31/124	0/93	0/93	0/93	None	NT	None
**Rotavirus**	NRS [23]	LM (vaccine)	21	21	NT	1/55	0/39	NT	NR	NR
		LM (placebo)	21	11	NT	0/30	0/21	-	NR	NR
**Smallpox**	CR [34]	Symptomatic infant	1	1	NT	NT	2/2	Infant rash	2/2	Vaccinia

Columns explained: Population, characteristics of main study population, symptomatic mother refers to a case report on maternal adverse events, symptomatic infant to a case report on adverse events in an infant whose lactating mother was vaccinated; Viral detection, detection of virus (targeted by vaccine) in samples collected from mother or infant; Vaccine strain confirmed, whether the detected virus in samples was further identified as vaccine versus wild-type virus strain; Infant serology, seropositive or seronegative indicating the presence or absence, respectively, of specific antibodies in serum; Infant symptoms, symptoms likely related to the virus in question. Abbreviations: ref, reference; n/N, number of mothers/infants included in the analysis of total sample size; HM, human milk; NRS, non-randomized study; CS, case series; CR, case report; RCT, randomized controlled trial; LM, lactating mother; +/total, + indicates positive samples of the total number of analyzed samples; NT, not tested; -, not applicable; CSF, cerebrospinal fluid; Pos. seropositive, Neg., seronegative; YEL-AND, yellow fever vaccine-associated neurotropic disease; YF, yellow fever. Red colored values indicate a positive result, or in other words, indicate the presence of signs of viral shedding.

## Supplementary Materials

The following supporting information can be downloaded at: https://www.mdpi.com/article/10.3390/vaccines13040350/s1, Table S1: Risk of bias assessment on study level, Table S2: Risk of bias assessment on outcome level, Table S3: Study Characteristics, Table S4: Adverse events in infants following vaccination of lactating mothers.

## Abbreviations

AE	Adverse event
CRS	Congenital rubella syndrome
GMT	Geometric mean titers
LAIV	Live-attenuated influenza vaccine
LLM	Large Language Model
MMR	Measles, mumps, rubella
PEP	Post-exposure prophylaxis
PrEP	Pre-exposure prophylaxis
PT	Pertussis toxoid
RCT	Randomized-Controlled Trial
VZV	Varicella zoster virus
WC	Whole cell
YF	Yellow fever
DTaP	Diptheria, Tetanus, acellular Pertussis (vaccine)
ELISA	Enzyme-linked immunosorbent assay
GRADE	Grading of Recommendations Assessment, Development and Evaluation
HDCV	Human diploid cell rabies vaccine
HRIG	Human rabies immunoglobulin
IgA	Immunoglobulin A
IgG	Immunoglobulin G
IgM	Immunoglobulin M
SIgA	Secretory Immunoglobulin A
IPV	Inactivated poliovirus vaccine
JAMA	Journal of the American Medical Association
NHLBI	National Heart, Lung and Blood Institute
NK	Natural killer (cells)
OPV	Oral polio vaccine
PCR	Polymerase chain reaction
RBD	Receptor-binding domain
RNA	Ribonucleic acid
mRNA	Messenger ribonucleic acid

## Appendix A. Database Searches

**Table A1:** Ovid/Medline Results (7 July 2022).

Search	Ovid/Medline Query—7 July 2022	Results
#5	exp Lactation/or exp Breast Feeding/or (lactation or breast-feeding or lactating or ((breast or lactic or mammary-gland or milk) adj3 (secretion* or excretion))).ti,ab,kf.	111,425
#4	exp Postpartum Period/or (puerper* or postpartum or post-partum or postnatal or post-natal or lactating or breast-feeding).ti,ab,kf.	263,010
#3	exp Vaccination/or exp Vaccines/or (vaccin* or immunis* or immuniz*).ti,ab,kf.	516,754
#2	1 and 2 and 3	1552
#1	4 not (exp Animals/not exp humans/)	1084

**Table A2:** Embase.com Results (7 July 2022).

Search	Embase Query—7 July 2022	Results
#6	#5 NOT ‘conference abstract’/it	1136
#5	#4 NOT ([animals]/lim NOT [humans]/lim)	1310
#4	#1 AND #2 AND #3	1682
#3	‘vaccination’/exp OR vaccin*:ti,ab,kw OR immunis*:ti,ab,kw OR immuniz*:ti,ab,kw	589,202
#2	‘puerperium’/exp OR puerper*:ti,ab,kw OR ‘postpartum’/exp OR postpartum:ti,ab,kw OR ‘post partum’:ti,ab,kw OR postnatal:ti,ab,kw OR ‘post natal’:ti,ab,kw OR lactating:ti,ab,kw OR ‘breast feeding’:ti,ab,kw	331,060
#1	‘lactation’/exp OR ‘breast feeding’/exp OR lactation:ti,ab,kw OR ‘breast feeding’:ti,ab,kw OR lactating:ti,ab,kw OR (((breast OR lactic OR ‘mammary gland’ OR milk) NEAR/3 (secretion* OR excretion)):ti,ab,kw)	139,582

**Table A3:** Clarivate Analytics/Web of Science Core Collection Results (7 July 2022).

Search	Web of Science Query—7 July 2022	Results
#4	#1 AND #2 AND #3	1832
#3	TS = (vaccin* OR immunis* OR immuniz*)	489,429
#2	TS = (puerper* OR postpartum OR post-partum OR postnatal OR post-natal OR lactating OR breast-feeding)	380,965
#1	TS = (lactation OR breast-feeding OR lactating OR ((breast OR lactic OR mammary-gland OR milk) NEAR/3 (secretion* OR excretion)))	229,211

## Appendix B. Python Script and Optimized System Prompt

import csv

import openai

import os

# Set OpenAI API key

openai.api_key = “YOUR_API_KEY_HERE”

# Input and output file paths

input_csv_file = “/path/to/input/file.csv”

output_csv_file = “/path/to/output/file.csv”

# System prompt for ChatGPT

system_prompt = “““

You are a professor in Medicine, part of the Breastfeeding and Lactation Scientific Interest Group. You are an expert in lactation research, and your task is to evaluate the titles and abstracts of scientific articles about lactation to determine their eligibility for inclusion in a literature review. Assess the relevance of articles based on their title and abstract and provide a concise explanation for your decision.

You are conducting a review of the literature to examine the effects of vaccination on lactating individuals or their offspring. The scope of this review is broad, research that could relate to the topic of interest should be included.

You will base your judgement on the following criteria (if none of these criteria are the specific focus of an article, but one of them seems to be of some significance, weigh this in your decision):

(i) Population: Lactating individuals and/or their offspring. Exclude articles focusing on specific diseases or conditions (such as cancer or Crohn’s disease patients) or targeting non-infectious agents (e.g., cancer vaccines). Studies encompassing both human and non-human subjects should be included.
(ii) Intervention:

One of the following:

- Vaccines administered to lactating individuals in the postpartum period.
- Offspring receiving breastmilk expressed by lactating individuals vaccinated in the postpartum period.

Exclude articles studying vaccines targeting non-infectious agents (for example cancer vaccines) and articles studying vaccination of newborns.

(iii) Outcome: The effect of vaccines on one or more of the following outcome categories:
  1. The lactating individual (examples: systemic immune response; mucosal immune response; adverse events or complications like vaccine-virus infection; side-effects; negative long-term effects; hazardous vaccine-components; etc.);
  2. The breastmilk (examples: breastmilk composition; excretion of vaccine components in breastmilk; excretion of vaccine-virus in breastmilk; milk supply; milk yield quality; excretion of hazardous vaccine ingredients in breastmilk, including mercury or thimerosal; etc.);
  3. The breastfed offspring (examples: transfer of vaccine-induced immunological factors; prevalence or incidence of vaccine-preventable infectious diseases; complications or hospital admissions; severity of the disease course in the offspring; side-effects in the offspring after administration of vaccines in lactating individuals; offspring infection with vaccine-virus; negative effects for the offspring of vaccination of their parent or indirect exposure to hazardous vaccine components such as mercury or thimerosal through breastfeeding; etc.). Include studies that focus on the indirect effects of vaccines on offspring, such as immunization through the transfer of immunological factors in breastmilk or potential harm due to exposure to hazardous vaccine components like mercury or thimerosal.

Exclude studies if they do not focus on any of the given outcome categories or if their focus is of little significance or potential relevance to the research question.

(iv) Publication type or study design: do not include literature reviews, scoping reviews, editorials, legal cases or interviews. These should ALWAYS receive a hard reject.

First, give a one sentence explanation for your judgement on every criterium, ending with a 1-10/10 rating describing the relevance to the subject of this literature review.

Then specify the final decision in the following format:

‘FINAL DECISION: 1-10/10’

where 1 is a hard reject and 10 is a definitive include.

Never include or exclude articles if you are not sure of your decision.

“““

def rate_article(title, abstract):

response = openai.ChatCompletion.create(

model = “gpt-4”,

messages = [

{“role”: “system”, “content”: system_prompt},

{“role”: “user”, “content”: f”Title: {title}\nAbstract: {abstract}”}

],

temperature = 0,

max_tokens = 2000

)

return response[‘choices’][0][‘message’][‘content’]

# Main process

data = []

with open(input_csv_file, mode = ‘r’, newline = ‘‘, encoding = ‘utf-8’) as file:

csv_reader = csv.DictReader(file)

for row in csv_reader:

title = row[‘title’]

abstract = row[‘abstract’]

try:

output = rate_article(title, abstract)

print(f”Title: {title}\nAbstract: {abstract}\nOutput: {output}\n”)

import re

numbers = re.findall(r’\d+/\d+’, output)

row[‘Population’] = numbers [0]

row[‘Intervention’] = numbers [1]

row[‘Outcome’] = numbers [2]

row[‘Publication type’] = numbers [3]

row[‘FINAL DECISION’] = numbers [4]

row[‘output’] = output

except Exception as e:

print(f”Error processing: {title}”)

print(e)

data.append(row)

# Write output

with open(output_csv_file, mode = ‘w’, newline = ‘‘, encoding = ‘utf-8’) as file:

fieldnames = data [0].keys()

csv_writer = csv.DictWriter(file, fieldnames=fieldnames)

csv_writer.writeheader()

csv_writer.writerows(data)

print(“Article rating complete. Results written to output file.”)

## Appendix C. Example of Output CSV for Citation Screening and Final Decisions

**Table A4:** This is an example of the output CSV file generated by the large-language model (LLM) for citation screening in the current comprehensive review. The columns reflect the LLM’s detailed criteria assessment and final decision ratings, ranging from 1 (hard reject) to 10 (definitive include). This appendix illustrates the format and content used to evaluate articles on the effects of vaccination on lactating individuals and their offspring.

Key	Population	Intervention	Outcome	Publication Type	FINAL DECISION	Output
Example 1	10/10	10/10	10/10	10/10	10/10	(i) Population: The study focuses on lactating sows and offspring, which falls within the scope of the review. 10/10 (ii) Intervention: The study investigates a novel VP2-based PPV1 subunit vaccine administered to bred pigs, including lactating sows. 10/10 (iii) Outcome: The study examines the safety of the vaccine in lactating sows and offspring, including survival rate and average daily weight gain in offspring. 10/10 (iv) Publication type or study design: The study is an experimental investigation, which is appropriate for inclusion. 10/10 FINAL DECISION: 10/10
Example 2	3/10	4/10	3/10	10/10	3/10	(i) Population: The article focuses on the general population in the Amazon Basin, including infants, but does not specifically mention lactating individuals or their offspring. 3/10 (ii) Intervention: The article discusses Thimerosal-containing vaccines (TCVs) as a source of mercury exposure, but does not specifically focus on vaccines administered to lactating individuals in the postpartum period or their breastfed offspring. 4/10 (iii) Outcome: The article addresses health effects associated with mercury exposure, including from TCVs, but does not specifically focus on the given outcome categories related to lactating individuals, breastmilk, or breastfed offspring. 3/10 (iv) Publication type or study design: The article appears to be a research article based on a search and selection of papers addressing mercury exposure and human health. 10/10 FINAL DECISION: 3/10
