# Supplementary material for: Safety and Efficacy of Vaccination During Lactation: A Comprehensive Review of Vaccines for Maternal and Infant Health Utilizing a Large Language Model Citation Screening System
Source: Vaccines (Basel). 2025 Mar 25;13(4):350. doi: 10.3390/vaccines13040350 (PMC12031549; doi:10.3390/vaccines13040350)
Supplement: Supplementary file 1 [file vaccines-13-00350-s001.zip › vaccines-3491364-supplementary.pdf]

Supplementary Materials

Table of Contents

Table S1. Risk of bias assessment on study level 2
S1.1. NIH Quality Assessment Tool of Controlled Intervention Studies. 2
S1.2. NIH Quality Assessment Tool for Observational Cohort and Cross-Sectional Studies. 2
S1.3. NIH Quality Assessment Tool of Case-Control Studies. 3
S1.4. NIH Quality Assessment Tool for Before-After (Pre-Post) Studies With No Control Group. 3
S1.5. NIH tool for Case Series Studies 3
S1.6. JBI Critical Appraisal Tool for Case Reports 3

Table S2. Risk of bias assessment on outcome level 5
S2.1. Risk of bias in randomized-studies (RoB 2 tool) 5
S2.2. Risk of bias in non-randomized studies (ROBINS-I tool) 5

Table S3. Study Characteristics 8

Table S4. Adverse events in infants following vaccination of lactating mothers 19

References 24

Key to reference 24

Table S1. Risk of bias assessment on study level

Caption Table S1.1. to S1.6. The National Heart, Lung, and Blood Institute (NHLBI) developed a set of quality assessment tools for specific study designs; controlled intervention studies (1.1), observational cohort and cross-sectional studies (1.2), case-control studies (1.3), before-after studies (1.4), case series (1.5).[1] Quality assessment of case reports was done using the Joanna Briggs Institute (JBI) Tool for Case Reports (1.6).[2] Each row in the table corresponds to a scientific research paper from the literature review. The unique key number in the Key column identifies each paper, and the full bibliographic reference for each key number is located at the end of this Supplementary Materials file under 'Key to reference'. Abbreviations: NR, not reported; CD, cannot determine; NA, not applicable.

S1.1. NIH Quality Assessment Tool of Controlled Intervention Studies.

Key	1	2	3	4	5	6	7	8	9	10	11	12	13	14	Rater (SJM)
2	Yes	NR	NR	Yes	Yes	Yes	Yes	NR	Yes	NR	Yes	NR	Yes	NR	Fair
3	No	NR	NR	NR	NR	NR	NR	NR	NR	Yes	Yes	NR	NR	NR	Poor
15	Yes	NR	NR	Yes	NR	Yes	Yes	Yes	Yes	Yes	Yes	NR	NA	CD	Fair
7	Yes	CD	Yes	Yes	Yes	Yes	Yes	Yes	Yes	Yes	Yes	Yes	Yes	Yes	Good
71	Yes	Yes	Yes	Yes	Yes	Yes	Yes?	Yes	Yes	Yes	Yes	Yes	Yes	Yes	Good

S1.2. NIH Quality Assessment Tool for Observational Cohort and Cross-Sectional Studies.

Key	1	2	3	4	5	6	7	8	9	10	11	12	13	14	Rater (SJM)
8	Yes	Yes	Yes?	Yes	Yes	No	No	NA	Yes	NA	Yes	NR	NA	No	Fair
9	Yes	Yes	NR	Yes	No	Yes	Yes	Yes	Yes	No	Yes	NR	NA	Yes	Fair
10	Yes	Yes	NR	Yes	No	Yes	Yes	Yes	Yes	Yes	Yes	NR	NR	Yes	Fair
13	Yes	Yes	NR	Yes	No	Yes	Yes	Yes	Yes	Yes	Yes	NR	NR	No	Fair-Good
19	Yes	Yes	NR	Yes	No	Yes	Yes	NA	Yes	Yes	Yes	NR	Yes	Yes?	Fair-Good
20	Yes	Yes	NR	Yes	No	Yes	Yes	NA	Yes	Yes	Yes	NR	Yes	Yes?	Fair-Good
21	Yes	Yes	NR	Yes	No	No	No	Yes	Yes	No	Yes	NR	NA	No	Poor-Fair
23	Yes	Yes	No	Yes	Yes	Yes	Yes	NA	Yes	Yes	Yes	NR	CD	No	Fair-Good
24	Yes	Yes	NR	Yes	No	Yes	Yes	NA	Yes	Yes	Yes	NR	Yes	No	Fair
27	Yes	Yes	Yes	Yes	No	Yes	Yes	NA	Yes	No	CD	No	Yes	Yes	Fair
47	Yes	Yes	NR	Yes	No	Yes	Yes	NA	Yes	Yes	Yes	NR	Yes	No	Fair
36	Yes	Yes	Yes	Yes	No	Yes	Yes	NA	Yes	No	Yes	NR	NA	No	Fair
34	Yes	Yes	Yes	Yes	No	Yes	Yes	Yes	CD	NA	Yes	NR	Yes	No	Fair
45	Yes	Yes	Yes	Yes	No	Yes	Yes	NA	Yes	No	Yes	NA	NR	No	Fair
31	Yes	Yes	Yes	Yes	No	Yes	Yes	Yes	Yes	NA	Yes	No	Yes	No	Fair-Good
33	Yes	Yes	Yes	Yes	No	Yes	Yes	NA	Yes	No	Yes	NR	NA	No	Fair
35	Yes	Yes	Yes	Yes	No	Yes	Yes	Yes	Yes	No	Yes	NR	NA	No	Fair-Good
37	Yes	Yes	Yes	Yes	No	Yes	Yes	NA	Yes	No	Yes	NR	Yes	No	Fair
38	Yes	Yes	Yes	Yes	No	Yes	Yes	No	No	NA	Yes	No	Yes	No	Fair
39	Yes	Yes	Yes	Yes	No	Yes	Yes	NA	Yes	NR	Yes	NR	NR	No	Fair
42	Yes	Yes	Yes	Yes	No	Yes	Yes	NA	Yes	NA	Yes	NA	Yes	No	Fair
43	Yes	Yes	Yes	Yes	No	Yes	Yes	No	Yes	NA	Yes	No	Yes	No	Fair
44	Yes	Yes	Yes	Yes	No	NA	No	NA	Yes	No	Yes	NA	NA	No	Poor-Fair
46	Yes	Yes	Yes	Yes	No	Yes	Yes	Yes	Yes	NA	Yes	No	Yes	No	Fair

**49**	Yes	Yes	Yes	Yes	No	No	No	NA	Yes	NR	Yes	Yes	CD	No	Fair
**50**	Yes	Yes	Yes	Yes	No	Yes	Yes	NA	Yes	Yes	Yes	NA	CD	No	Fair
**51**	Yes	Yes	Yes	Yes	No	Yes	Yes	NA	Yes	NA	Yes	NA	Yes	No	Fair
**52**	Yes	Yes	Yes	Yes	No	Yes	Yes	Yes	Yes	Yes	Yes	NR	CD	Yes	Fair
**53**	Yes	Yes	Yes	Yes	No	Yes	Yes	NA	Yes	No	Yes	NR	Yes	No	Fair
**54**	Yes	Yes	Yes	Yes	Yes	Yes	Yes	No	Yes	Yes	Yes	Yes	NR	Yes	Good
**55**	Yes	Yes	Yes	Yes	No	Yes	Yes	NA	Yes	NA	Yes	No	Yes	No	Fair
**56**	Yes	Yes	Yes	Yes	No	Yes	No	NA	Yes	NA	CD	NA	Yes	No	Fair-Good
**57**	Yes	Yes	Yes	Yes	No	Yes	Yes	NA	Yes	Yes	Yes	NR	CD	No	Fair
**59**	Yes	Yes	Yes	Yes	No	Yes	Yes	No	Yes	Yes	Yes	NR	CD	No	Fair
**60**	Yes	Yes	Yes	CD	No	Yes	Yes	NA	Yes	CD	CD	NR	NA	No	Fair
**61**	Yes	Yes	Yes	Yes	No	Yes	CD	NA	Yes	Yes	Yes	NR	NA	No	Fair
**63**	Yes	Yes	Yes	Yes	No	Yes	Yes	Yes	Yes	NA	Yes	NA	Yes	No	Fair
**64**	Yes	Yes	Yes	Yes	No	Yes	Yes	NA	Yes	Yes	Yes	NR	NR	No	Fair
**65**	Yes	Yes	Yes	Yes	No	Yes	CD	NA	Yes	No	Yes	NR	NR	No	Fair
**66**	Yes	Yes	Yes	Yes	No	Yes	Yes	Yes	Yes	NA	Yes	NA	Yes	No	Fair
**67**	Yes	Yes	Yes	Yes	No	Yes	CD	Yes	Yes	Yes	Yes	NR	CD	No	Fair
**69**	Yes	Yes	Yes	Yes	No	Yes	Yes	NA	Yes	NA	Yes	NA	NR	No	Fair
**70**	Yes	Yes	Yes	Yes	No	Yes	Yes	No	Yes	Yes	Yes	NR	Yes	No	Fair

S1.3. NIH Quality Assessment Tool of Case-Control Studies.

**Key**	**1**	**2**	**3**	**4**	**5**	**6**	**7**	**8**	**9**	**10**	**11**	**12**	**Rater (SJM)**
**1**	Yes	Yes	No	Yes	Yes	Yes	Yes	Yes	Yes	Yes	CD	Yes	Good
**11**	Yes	Yes	Yes	Yes	Yes	Yes	Yes	Yes	Yes	Yes	NR	Yes	Good

S1.4. NIH Quality Assessment Tool for Before-After (Pre-Post) Studies With No Control Group.

**Key**	**1**	**2**	**3**	**4**	**5**	**6**	**7**	**8**	**9**	**10**	**11**	**12**	**Rater (SJM)**
**30**	Yes	Yes?	No?	NR	No?	Yes	Yes	NR	NR	Yes	No?	NA	Fair
**32**	Yes	Yes?	No?	Yes	No?	Yes	Yes	NR	Yes	Yes	Yes	NA	Fair
**12**	Yes	Yes	No	Yes	No	Yes	Yes	NR	NR	No	No	NA	Poor
**17**	Yes	No	Yes	No	Yes	Yes	Yes	NR	NR	Yes	No	NA	Fair
**4**	Yes	Yes	Yes	NR	No	Yes	Yes	No	NA	NR	Yes	NA	Poor
**5**	Yes	No	CD	No	No	Yes	Yes	No	Yes	CD	CD	NA	Poor
**62**	Yes	Yes	Yes?	No?	No?	Yes	Yes	NR	NR	Yes	No?	NA	Fair
**6**	Yes	Yes	Yes	CD	No	Yes	Yes	CD	NA	Yes	No	NA	Poor
**68**	Yes	Yes	Yes?	No?	No?	Yes	Yes	NR	NR	Yes	Yes	NA	Fair

S1.5. NIH tool for Case Series Studies

**Key**	**1**	**2**	**3**	**4**	**5**	**6**	**7**	**8**	**9**	**Rater (SJM)**
25	Yes	Yes	NR	Yes	Yes	Yes	Yes	No	Yes	Fair
48	Yes	Yes	CD	Yes	Yes	Yes	Yes	NA	Yes	Fair
58	Yes	Yes	No	Yes	Yes	Yes	Yes	NA	Yes	Fair

S1.6. JBI Critical Appraisal Tool for Case Reports

Key	1	2	3	4	5	6	7	8	Rater (SJM)
16	Yes	Yes	Yes	Yes	Yes	Yes	Yes	Yes	Good
18	Yes	Yes	Yes	Yes	Yes	Yes	Unclear	Yes	Good
14	Yes	Yes	Yes	Yes	Yes	Yes	Yes	Yes	Good
22	Yes	Yes	Yes	Yes	Yes	Yes	Yes	Yes	Good
26	Yes	Yes	Yes	Yes	Yes	Yes	No	Yes	Good
28	Yes	Yes	Yes	Yes	Yes	Yes	Unclear	Yes	Good
29	Yes	Yes	Yes	Yes	Yes	Yes	Unclear	Yes	Good
40	Yes	Yes	Yes	No	Unclear	Yes	Yes	Yes	Fair
41	Yes	No	Yes	Yes	Yes	Yes	Yes	Yes	Good
72	Yes	Yes	Yes	Yes	Yes	Yes	No	Yes	Good
73	Yes	Yes	Yes	Yes	Yes	Yes	Yes	Yes	Good

Table S2. Risk of bias assessment on outcome level

Caption Table S2. Risk of bias was assessed in each study for each outcome of interest. Randomized studies were assessed using the Revised Risk of Bias tool (RoB 2 tool)[3], examining each outcome of interest on five bias domains (Table 2.1). Non-randomized studies were assessed using the Risk of Bias in Non-randomized Studies of Interventions tool (ROBINS-I)[4] which assessed findings on each outcome of interest on seven bias domains (Table 2.2). Each study was assessed on the risk of bias for each outcome (of interest to this review); five (for randomized studies) or seven (for non-randomized studies) bias domains and an overall risk of bias were graded with 'low', 'moderate', 'serious', or 'critical' which are represented by the green, yellow, orange, or red symbol, respectively. Each row in the table corresponds to a scientific research paper from the literature review. The unique key number in the '**Key'** column identifies each paper, and the full bibliographic reference for each key number is located at the end of this Supplementary Materials file under 'Key to reference.' Abbreviations. NA, not applicable; [ROB 2 tool] 1D1-1D5, outcome 1 and bias domain 1 to 5; O1, overall risk of bias for outcome 1; 2D1-2D5, outcome 2 and bias domain 1 to 5; O2, overall risk of bias for outcome 2; 3D1-3D5, outcome 3 and bias domain 1 to 5; O3, overall risk of bias for outcome 3; 4D1-4D5, outcome 4 and bias domain 1 to 5; O4, overall risk of bias for outcome 4; 5D1-5D5, outcome 5 and bias domain 1 to 5; O5, overall risk of bias for outcome 5; [ROBINS-I tool] 1D1-1D7, outcome 1 and bias domain 1 to 7; O1, overall risk of bias for outcome 1; 2D1-2D7, outcome 2 and bias domain 1 to 7; O2, overall risk of bias for outcome 2; 3D1-3D7, outcome 3 and bias domain 1 to 7; O3, overall risk of bias for outcome 3; 4D1-4D7, outcome 4 and bias domain 1 to 7; O4, overall risk of bias for outcome 4; 5D1-5D7, outcome 5 and bias domain 1 to 7; O5, overall risk of bias for outcome 5.

S2.1. Risk of bias in randomized-studies (RoB 2 tool)

Vaccine	Key	1D1	1D2	1D3	1D4	1D5	O1	2D1	2D2	2D3	2D4	2D5	O2	3D1	3D2	3D3	3D4	3D5	O3	4D1	4D2	4D3	4D4	4D5	O4	5D1	5D2	5D3	5D4	5D5	O5
Rubella	71	🟢	🟢	🟢	🟢	🟢	🟢	NA	NA	NA	NA	NA	NA	NA	NA	NA	NA	NA	NA	NA	NA	NA	NA	NA	NA	NA	NA	NA	NA	NA	NA
Rotavirus	15	🟢	🟡	🟢	🟢	🟢	🟢	🟢	🟡	🟢	🟢	🟢	🟢	🟢	🟡	🟢	🟡	🟢	🟡	🟢	🟡	🟢	🟢	🟢	🟢	NA	NA	NA	NA	NA	NA
Cholera	2	NA	NA	NA	NA	NA	NA	NA	NA	NA	NA	NA	NA	🟢	🟢	🟡	🟢	🟢	🟢	🟢	🟢	🟢	🟢	🟡	🟢	NA	NA	NA	NA	NA	NA
	3	NA	NA	NA	NA	NA	NA	NA	NA	NA	NA	NA	NA	🟡	🟡	🟠	🟢	🟡	🟡	🟡	🟡	🟠	🟢	🟠	🟠	NA	NA	NA	NA	NA	NA
Influenza	7	🟢	🟢	🟢	🟢	🟢	🟢	🟢	🟢	🟢	🟢	🟡	🟢	🟢	🟢	🟢	🟢	🟡	🟢	🟢	🟢	🟢	🟢	🟢	🟢	NA	NA	NA	NA	NA	NA

S2.2. Risk of bias in non-randomized studies (ROBINS-I tool)

Vaccine	Key	1D1	1D2	1D3	1D4	1D5	1D6	1D7	O1	2D1	2D2	2D3	2D4	2D5	2D6	2D7	O2	3D1	3D2	3D3	3D4	3D5	3D6	3D7	O3	4D1	4D2	4D3	4D4	4D5	4D6	4D7	O4	5D1	5D2	5D3	5D4	5D5	5D6	5D7	O5
Cholera	1	NA	NA	NA	NA	NA	NA	NA	NA	NA	NA	NA	NA	NA	NA	NA	NA	NA	NA	NA	NA	NA	NA	NA	NA	NA	NA	NA	NA	NA	NA	NA	NA	🟡	🟡	🟢	🟢	🟡	🟡	🟢	🟡
	4	NA	NA	NA	NA	NA	NA	NA	NA	NA	NA	NA	NA	NA	NA	NA	NA	🟡	🟡	🟢	🟢	🟡	🟢	🟢	🟡	🟡	🟡	🟢	🟢	🟡	🟢	🟢	🟡	NA	NA	NA	NA	NA	NA	NA	NA
	5	NA	NA	NA	NA	NA	NA	NA	NA	NA	NA	NA	NA	NA	NA	NA	NA	🟠	🟡	🟢	🟢	🟡	🟢	🟢	🟠	🟠	🟡	🟢	🟢	🟡	🟢	🟢	🟠	NA	NA	NA	NA	NA	NA	NA	NA
	6	NA	NA	NA	NA	NA	NA	NA	NA	NA	NA	NA	NA	NA	NA	NA	NA	🟡	🟡	🟢	🟢	🟡	🟢	🟢	🟡	🟡	🟡	🟢	🟢	🟡	🟢	🟢	🟡	NA	NA	NA	NA	NA	NA	NA	NA
Influenza	8	NA	NA	NA	NA	NA	NA	NA	NA	NA	NA	NA	NA	NA	NA	NA	NA	🟠	🟡	🟢	🟡	🟡	🟢	🟡	🟠	NA	NA	NA	NA	NA	NA	NA	NA	NA	NA	NA	NA	NA	NA	NA	NA
Pertussis	9	🟡	🟡	🟡	🟢	🟢	🟡	🟡	🟡	NA	NA	NA	NA	NA	NA	NA	NA	NA	NA	NA	NA	NA	NA	NA	NA	NA	NA	NA	NA	NA	NA	NA	NA	NA	NA	NA	NA	NA	NA	NA	NA
	10	🟡	🟡	🟡	🟢	🟡	🟡	🟢	🟡	🟡	🟡	🟡	🟢	🟠	🟡	🟡	🟠	NA	NA	NA	NA	NA	NA	NA	NA	NA	NA	NA	NA	NA	NA	NA	NA	NA	NA	NA	NA	NA	NA	NA	NA
	11	NA	NA	NA	NA	NA	NA	NA	NA	NA	NA	NA	NA	NA	NA	NA	NA	NA	NA	NA	NA	NA	NA	NA	NA	NA	NA	NA	NA	NA	NA	NA	NA	🟢	🟡	🟡	🟢	🟠	🟢	🟢	🟡

Pneu	12	NA	NA	NA	NA	NA	NA	NA	NA	NA	NA	NA	NA	NA	NA	NA	NA	🟡	🟢	🟡	🟢	🟢	🟡	🟠	🟡	🟡	🟢	🟡	🟢	🟢	🟡	🟠	🟡	NA	NA	NA	NA	NA	NA	NA	NA
Polio	13	NA	NA	NA	NA	NA	NA	NA	NA	NA	NA	NA	NA	NA	NA	NA	NA	🟠	🟡	🟡	🟡	🟡	🟢	🟠	🟠	🟠	🟠	🟡	🟡	🟡	🟡	🟠	🟠	NA	NA	NA	NA	NA	NA	NA	NA
Rubella	17	🟡	🟢	🟢	🟢	🟡	🟡	🟢	🟡	🟡	🟢	🟢	🟢	🟡	🟡	🟢	🟡	🟡	🟢	🟢	🟢	🟡	🟢	🟢	🟡	🟡	🟡	🟢	🟢	🟠	🟢	🟢	🟠	NA	NA	NA	NA	NA	NA	NA	NA
	19	🟡	🟢	🟡	🟢	🟢	🟡	🟡	🟡	🟡	🟢	🟡	🟢	🟢	🟡	🟡	🟡	🟡	🟢	🟢	🟢	🟢	🟡	🟡	🟡	🟡	🟢	🟢	🟢	🟢	🟡	🟡	🟡	NA	NA	NA	NA	NA	NA	NA	NA
	20	NA	NA	NA	NA	NA	NA	NA	NA	🟡	🟢	🟢	🟢	🟢	🟢	🟡	🟡	NA	NA	NA	NA	NA	NA	NA	NA	🟡	🟢	🟢	🟢	🟢	🟢	🟢	🟢	NA	NA	NA	NA	NA	NA	NA	NA
	21	🟡	🟢	🟢	🟢	🟡	🟡	🟢	🟡	NA	NA	NA	NA	NA	NA	NA	NA	🟡	🟡	🟢	🟡	🟡	🟢	🟢	🟡	NA	NA	NA	NA	NA	NA	NA	NA	NA	NA	NA	NA	NA	NA	NA	NA
Varicella	23	🟢	🟢	🟢	🟢	🟡	🟢	🟢	🟢	🟢	🟢	🟢	🟢	🟡	🟢	🟢	🟢	🟢	🟢	🟢	🟢	🟡	🟢	🟢	🟢	🟢	🟢	🟢	🟢	🟡	🟢	🟢	🟢	NA	NA	NA	NA	NA	NA	NA	NA
YF	24	NA	NA	NA	NA	NA	NA	NA	NA	🟡	🟠	🟢	🟡	🟠	🟡	🟠	🟠	🟠	🟠	🟢	🟡	🟢	🟠	🟠	🟢	NA	NA	NA	NA	NA	NA	NA	NA	NA	NA	NA	NA	NA	NA	NA	NA
	25	NA	NA	NA	NA	NA	NA	NA	NA	🟠	🟠	🟢	🟡	🟡	🟠	🟠	🟠	🟡	🟠	🟢	🟢	🟡	🟡	🟡	🟠	NA	NA	NA	NA	NA	NA	NA	NA	NA	NA	NA	NA	NA	NA	NA	NA
	27	🟡	🟡	🟢	🟢	🟠	🟠	🟡	🟠	🟡	🟡	🟢	🟢	🟠	🟠	🟡	🟠	NA	NA	NA	NA	NA	NA	NA	NA	NA	NA	NA	NA	NA	NA	NA	NA	NA	NA	NA	NA	NA	NA	NA	NA
COVID-19	30	NA	NA	NA	NA	NA	NA	NA	NA	NA	NA	NA	NA	NA	NA	NA	NA	🟡	🟢	🟢	🟢	🟡	🟢	🟡	🟡	🟡	🟢	🟢	🟢	🟡	🟢	🟢	🟡	NA	NA	NA	NA	NA	NA	NA	NA
	31	NA	NA	NA	NA	NA	NA	NA	NA	NA	NA	NA	NA	NA	NA	NA	NA	🟡	🟡	🟢	🟢	🟢	🟢	🟡	🟡	🟡	🟢	🟢	🟢	🟡	🟢	🟢	🟡	NA	NA	NA	NA	NA	NA	NA	NA
	32	NA	NA	NA	NA	NA	NA	NA	NA	NA	NA	NA	NA	NA	NA	NA	NA	NA	NA	NA	NA	NA	NA	NA	NA	🟡	🟢	🟢	🟢	🟢	🟢	🟢	🟢	NA	NA	NA	NA	NA	NA	NA	NA
	33	NA	NA	NA	NA	NA	NA	NA	NA	NA	NA	NA	NA	NA	NA	NA	NA	🟡	🟢	🟢	🟢	🟢	🟢	🟢	🟢	🟡	🟢	🟢	🟢	🟢	🟢	🟢	🟢	NA	NA	NA	NA	NA	NA	NA	NA
	34	🟡	🟡	🟢	🟢	🟢	🟡	🟢	🟡	🟡	🟢	🟢	🟢	🟢	🟡	🟢	🟡	NA	NA	NA	NA	NA	NA	NA	NA	NA	NA	NA	NA	NA	NA	NA	NA	NA	NA	NA	NA	NA	NA	NA	NA
	35	NA	NA	NA	NA	NA	NA	NA	NA	NA	NA	NA	NA	NA	NA	NA	NA	NA	NA	NA	NA	NA	NA	NA	NA	🟡	🟡	🟢	🟢	🟡	🟢	🟡	🟡	NA	NA	NA	NA	NA	NA	NA	NA
	36	NA	NA	NA	NA	NA	NA	NA	NA	🟡	🟢	🟢	🟢	🟢	🟡	🟢	🟡	NA	NA	NA	NA	NA	NA	NA	NA	🟡	🟢	🟢	🟢	🟢	🟢	🟢	🟢	NA	NA	NA	NA	NA	NA	NA	NA
	37	🟡	🟡	🟢	🟢	🟢	🟡	🟢	🟡	🟡	🟠	🟢	🟢	🟡	🟡	🟡	🟠	🟡	🟡	🟢	🟢	🟡	🟢	🟡	🟡	🟡	🟢	🟢	🟢	🟡	🟢	🟡	🟡	NA	NA	NA	NA	NA	NA	NA	NA
	38	🟡	🟡	🟢	🟢	🟢	🟡	🟢	🟡	NA	NA	NA	NA	NA	NA	NA	NA	🟡	🟡	🟢	🟢	🟡	🟢	🟡	🟡	🟡	🟢	🟢	🟢	🟡	🟢	🟢	🟡	NA	NA	NA	NA	NA	NA	NA	NA
	39	NA	NA	NA	NA	NA	NA	NA	NA	NA	NA	NA	NA	NA	NA	NA	NA	🟡	🟢	🟢	🟢	🟡	🟢	🟢	🟡	🟡	🟢	🟢	🟢	🟡	🟢	🟢	🟡	NA	NA	NA	NA	NA	NA	NA	NA
	42	NA	NA	NA	NA	NA	NA	NA	NA	NA	NA	NA	NA	NA	NA	NA	NA	🟡	🟢	🟢	🟢	🟡	🟢	🟢	🟡	🟡	🟢	🟢	🟢	🟢	🟢	🟢	🟢	NA	NA	NA	NA	NA	NA	NA	NA
	43	🟡	🟡	🟢	🟢	🟡	🟡	🟢	🟡	🟡	🟢	🟢	🟢	🟡	🟢	🟢	🟡	🟡	🟢	🟢	🟢	🟡	🟢	🟢	🟡	🟡	🟢	🟢	🟢	🟡	🟢	🟢	🟡	NA	NA	NA	NA	NA	NA	NA	NA
	44	NA	NA	NA	NA	NA	NA	NA	NA	🟡	🟠	🟢	🟢	🟢	🟢	🟡	🟡	NA	NA	NA	NA	NA	NA	NA	NA	NA	NA	NA	NA	NA	NA	NA	NA	NA	NA	NA	NA	NA	NA	NA	NA
	45	NA	NA	NA	NA	NA	NA	NA	NA	NA	NA	NA	NA	NA	NA	NA	NA	🟡	🟢	🟢	🟢	🟡	🟢	🟢	🟡	🟡	🟡	🟢	🟢	🟡	🟢	🟡	🟡	NA	NA	NA	NA	NA	NA	NA	NA
	46	🟡	🟡	🟢	🟢	🟢	🟡	🟢	🟡	NA	NA	NA	NA	NA	NA	NA	NA	🟡	🟢	🟢	🟢	🟡	🟢	🟢	🟡	🟡	🟢	🟢	🟢	🟢	🟢	🟢	🟢	NA	NA	NA	NA	NA	NA	NA	NA
	47	🟡	🟡	🟢	🟢	🟡	🟡	🟢	🟡	NA	NA	NA	NA	NA	NA	NA	NA	🟡	🟡	🟢	🟢	🟡	🟢	🟢	🟡	🟡	🟢	🟢	🟢	🟢	🟢	🟢	🟢	NA	NA	NA	NA	NA	NA	NA	NA
	49	🟡	🟡	🟢	🟢	🟢	🟡	🟢	🟡	🟡	🟡	🟢	🟢	🟢	🟢	🟢	🟡	🟡	🟡	🟢	🟢	🟡	🟢	🟢	🟡	🟡	🟡	🟢	🟢	🟡	🟢	🟡	🟡	NA	NA	NA	NA	NA	NA	NA	NA
	50	🟡	🟡	🟢	🟢	🟢	🟡	🟢	🟡	NA	NA	NA	NA	NA	NA	NA	NA	NA	NA	NA	NA	NA	NA	NA	NA	🟡	🟢	🟢	🟢	🟡	🟢	🟢	🟡	NA	NA	NA	NA	NA	NA	NA	NA
	51	NA	NA	NA	NA	NA	NA	NA	NA	NA	NA	NA	NA	NA	NA	NA	NA	🟡	🟡	🟢	🟢	🟢	🟢	🟢	🟡	🟡	🟢	🟢	🟢	🟢	🟢	🟢	🟢	NA	NA	NA	NA	NA	NA	NA	NA
	52	NA	NA	NA	NA	NA	NA	NA	NA	NA	NA	NA	NA	NA	NA	NA	NA	🟡	🟡	🟢	🟢	🟡	🟢	🟢	🟡	🟡	🟡	🟢	🟢	🟡	🟢	🟢	🟡	NA	NA	NA	NA	NA	NA	NA	NA
	53	🟡	🟡	🟢	🟢	🟢	🟡	🟢	🟡	🟠	🟡	🟢	🟢	🟢	🟢	🟡	🟠	NA	NA	NA	NA	NA	NA	NA	NA	NA	NA	NA	NA	NA	NA	NA	NA	NA	NA	NA	NA	NA	NA	NA	NA
	54	🟡	🟡	🟢	🟢	🟢	🟡	🟢	🟡	NA	NA	NA	NA	NA	NA	NA	NA	🟡	🟡	🟢	🟢	🟡	🟢	🟡	🟡	🟡	🟢	🟢	🟢	🟢	🟢	🟢	🟢	NA	NA	NA	NA	NA	NA	NA	NA
	55	🟡	🟡	🟢	🟢	🟢	🟡	🟢	🟡	🟡	🟡	🟢	🟢	🟢	🟢	🟢	🟡	NA	NA	NA	NA	NA	NA	NA	NA	🟡	🟢	🟢	🟢	🟢	🟢	🟢	🟢	NA	NA	NA	NA	NA	NA	NA	NA
	56	🟡	🟡	🟢	🟢	🟢	🟡	🟢	🟡	🟡	🟡	🟢	🟢	🟢	🟢	🟢	🟡	NA	NA	NA	NA	NA	NA	NA	NA	NA	NA	NA	NA	NA	NA	NA	NA	NA	NA	NA	NA	NA	NA	NA	NA
	57	NA	NA	NA	NA	NA	NA	NA	NA	NA	NA	NA	NA	NA	NA	NA	NA	NA	NA	NA	NA	NA	NA	NA	NA	🟡	🟢	🟢	🟢	🟢	🟢	🟢	🟢	NA	NA	NA	NA	NA	NA	NA	NA
	58	NA	NA	NA	NA	NA	NA	NA	NA	NA	NA	NA	NA	NA	NA	NA	NA	NA	NA	NA	NA	NA	NA	NA	NA	🟡	🟡	🟢	🟢	🟡	🟢	🟡	🟡	NA	NA	NA	NA	NA	NA	NA	NA

	59	🟡	🟡	🟢	🟢	🟢	🟡	🟢	🟡	🟡	🟢	🟢	🟢	🟡	🟢	🟢	🟡	🟡	🟢	🟢	🟢	🟡	🟢	🟢	🟡	🟡	🟢	🟢	🟢	🟡	🟢	🟢	🟡	NA	NA	NA	NA	NA	NA	NA	NA
	60	🟡	🟡	🟢	🟢	🟢	🟡	🟢	🟡	NA	NA	NA	NA	NA	NA	NA	NA	🟡	🟢	🟢	🟢	🟡	🟢	🟢	🟡	🟡	🟢	🟢	🟢	🟢	🟢	🟢	🟢	NA	NA	NA	NA	NA	NA	NA	NA
	61	NA	NA	NA	NA	NA	NA	NA	NA	NA	NA	NA	NA	NA	NA	NA	NA	🟡	🟢	🟢	🟢	🟡	🟢	🟢	🟡	🟡	🟢	🟢	🟢	🟡	🟢	🟢	🟡	NA	NA	NA	NA	NA	NA	NA	NA
	62	NA	NA	NA	NA	NA	NA	NA	NA	NA	NA	NA	NA	NA	NA	NA	NA	🟡	🟢	🟢	🟢	🟡	🟢	🟢	🟡	🟡	🟢	🟢	🟢	🟢	🟢	🟢	🟢	NA	NA	NA	NA	NA	NA	NA	NA
	63	🟡	🟡	🟢	🟢	🟢	🟡	🟢	🟡	🟡	🟡	🟢	🟢	🟡	🟢	🟢	🟡	🟡	🟢	🟢	🟢	🟡	🟢	🟢	🟡	🟡	🟢	🟢	🟢	🟢	🟢	🟢	🟢	NA	NA	NA	NA	NA	NA	NA	NA
	64	🟡	🟡	🟢	🟢	🟢	🟡	🟢	🟡	NA	NA	NA	NA	NA	NA	NA	NA	🟡	🟡	🟢	🟢	🟡	🟢	🟢	🟡	🟡	🟢	🟢	🟢	🟡	🟢	🟢	🟡	NA	NA	NA	NA	NA	NA	NA	NA
	65	NA	NA	NA	NA	NA	NA	NA	NA	NA	NA	NA	NA	NA	NA	NA	NA	NA	NA	NA	NA	NA	NA	NA	NA	🟡	🟢	🟢	🟢	🟡	🟢	🟡	🟡	NA	NA	NA	NA	NA	NA	NA	NA
	66	🟡	🟡	🟢	🟢	🟢	🟡	🟢	🟡	NA	NA	NA	NA	NA	NA	NA	NA	🟡	🟢	🟢	🟢	🟡	🟢	🟢	🟡	🟡	🟢	🟢	🟢	🟡	🟢	🟢	🟡	NA	NA	NA	NA	NA	NA	NA	NA
	67	NA	NA	NA	NA	NA	NA	NA	NA	NA	NA	NA	NA	NA	NA	NA	NA	🟡	🟢	🟢	🟢	🟡	🟢	🟢	🟡	🟡	🟢	🟢	🟢	🟢	🟢	🟢	🟢	NA	NA	NA	NA	NA	NA	NA	NA
	68	NA	NA	NA	NA	NA	NA	NA	NA	NA	NA	NA	NA	NA	NA	NA	NA	NA	NA	NA	NA	NA	NA	NA	NA	🟡	🟢	🟢	🟢	🟢	🟢	🟢	🟢	NA	NA	NA	NA	NA	NA	NA	NA
	69	🟡	🟡	🟢	🟢	🟢	🟡	🟢	🟡	🟡	🟢	🟢	🟢	🟢	🟢	🟢	🟢	🟡	🟢	🟢	🟢	🟢	🟢	🟢	🟡	🟡	🟢	🟢	🟢	🟢	🟢	🟢	🟢	NA	NA	NA	NA	NA	NA	NA	NA
	70	NA	NA	NA	NA	NA	NA	NA	NA	🟡	🟠	🟢	🟢	🟢	🟢	🟡	🟡	NA	NA	NA	NA	NA	NA	NA	NA	🟡	🟡	🟢	🟢	🟡	🟢	🟢	🟡	NA	NA	NA	NA	NA	NA	NA	NA

Table S3. Study Characteristics

Caption Table S3. Each row in the table corresponds to a scientific research paper from the literature review. The unique key number in the Key column identifies each paper, and the full bibliographic reference for each key number is located at the end of this Supplementary Materials file under 'Key to reference.'

Key	Vaccine	Country, Year	Study Design	No. LM	Population type	No. infants	Age infant at vaccination LM	Comparison	Vaccine type	Follow-up
1	Cholera	Bangladesh 1985-1986	Case-control Study (including data from Randomized-Controlled Trial)	270	• Case mothers, Vaccine (n=28)<br>• Case mothers, Placebo (n=26)<br>• Control mothers, Vaccine (n=145)<br>• Control mothers, Placebo (n=71)	580	~25 months (mean)	1. Human milk NOT part of diet (non-breastfed)<br>2. Maternal receipt of placebo, inactivated E.coli K12 strain (three oral doses)	• Inactivated vaccine, combined B-subunit and killed whole-cell, enteral route (per oral) (n=87)<br>• Inactivated vaccine, killed whole-cell, enteral route (per oral) (n=86)<br>• Placebo (E. coli K12 strain), enteral (per oral) (n=97)	8 to 18 months
2	Cholera	Bangladesh 1985-1986	Randomized controlled trial	53	Vaccinated (n=34)<br>Placebo (n=19)	0	NR	• Different cholera vaccines<br>- BS-WC<br>- WC<br>• Control cohort (placebo)	• Inactivated vaccine, combined B-subunit and killed whole-cell, enteral route (per oral) (n=18)<br>• Inactivated vaccine, killed whole-cell, enteral route (per oral) (n=16)<br>• Placebo (E. coli K12 strain), enteral (per oral) (n=19)	14 weeks
3	Cholera<br>Typhoid	Pakistan 1989	Randomized controlled trial	49	Infection-primed:<br>• Vaccinated (n=39)<br>• Unvaccinated (n=10)	0	2 to 4 weeks	• Cholera vaccine (inactivated, subcutaneous)<br>• Typhoid vaccine (live, oral)<br>• Simultaneous administration of both vaccines	• Inactivated cholera vaccine, killed whole-cell, parenteral route (subcutaneous) (n=10)<br>• Live typhoid vaccine, Salmonella Typhi 21a, enteral (per os) (n=10)<br>• Combination of live typhoid vaccine (per os) and inactivated cholera vaccine (WC) (subcutaneous) (n=10)	24 weeks after the first vaccine dose.
4	Cholera<br>Typhoid	Pakistan 1988	Cohort Study (Prospective)	6	Vaccinated	0	≥2 weeks	Single cohort study (no comparison cohort)	Combination of live typhoid vaccine, Salmonella Typhi 21a, enteral (per os) and inactivated whole-cell (WC) cholera vaccine, parenteral (subcutaneous)	8 weeks after first vaccine dose.
5	Cholera	Bangladesh 1980	Cohort Study (Prospective)	9	Infection-primed:<br>• Vaccinated (n=6)<br>• *Active* Placebo (n=3)	0	NR	Control cohort (placebo)	• Inactivated cholera vaccine, parenteral (n=6): cholera toxoid or combined B-subunit and killed whole-cell vibrio cholerae (BS-WC)<br>• Placebo (inactivated typhoid toxoid vaccine), parenteral (subcutaneous) (n=3)	Varying follow-up duration between participants; up to 8 months observed for one participant

6	Cholera	Pakistan, Sweden 1977-1979	Cohort Study (Prospective)	20	Vaccinated (n=20)<br>• Immune-naïve (Sweden) (n=10)<br>• Infection-primed (Pakistan) (n=10)	0	1 to 10 months	Immunization status	Inactivated vaccine, bivalent killed whole-cell, parenteral route (subcutaneous)	2 weeks after vaccination.
7	Influenza	United States 2011-2013	Randomized-Controlled trial	248	Vaccinated	248	median of 70 days	Other vaccine type (different type and route of administration)	• Influenza vaccine, live-attenuated (LAIV) (intranasal) + Placebo (intramuscular) (n=124)<br>• Influenza vaccine, inactivated (IIV) (intramuscular) + Placebo (intranasal) (n=124)	180 days
8	Influenza	United States 2020	Cross-sectional study	16	Vaccinated (n=8)<br>Unvaccinated (n=8)	0	median of 5 months (3 to 10 months)	No vaccination	Influenza vaccine, flu season 2019-2020 (no information available regarding type of vaccine received)	Not applicable
9	Pertussis	Belgium 2012-2015	Cross-Sectional Analysis within Cohort Framework	34	Vaccination schedule/timing:<br>• during pregnancy (n=19)<br>• postpartum (n=34)<br>• <5 years prior (n=9)<br>• >5 years prior (n=12)	0	median of 58 days	• Timing of vaccination<br>• No vaccination	Pertussis-containing vaccine (Tdap) ('Boostrix'), combined tetanus, diphteria, acellular pertussis vaccine (i.m.)	8-9 weeks postpartum
10	Pertussis	Belgium 2015-2019	Cohort Study (Prospective)	16	Vaccinated during pregnancy (n=213)<br>• Term delivery (n=87)<br>• Preterm delivery (n=63)<br>Vaccinated postpartum* or unvaccinated (n=27)<br>• Term delivery (n=15)<br>• Preterm delivery (n=12)<br><br>*61% vaccinated after delivery	0	within 72 hours	• Timing of vaccination<br>• No vaccination	Pertussis-containing vaccine (Tdap) ('Boostrix'), combined tetanus, diphteria, acellular pertussis vaccine (i.m.)	12 weeks postpartum
11	Pertussis	Australia 2010-2011	Case-control study	352	• Case mothers, vaccination status:<br>- Vaccinated in pregnancy (n=12)<br>- Vaccinated postpartum (n=79)<br>- Unvaccinated (n=54)<br>• Control mothers, vaccination status:<br>- Vaccinated pre-/in pregnancy (n=37)<br>- Vaccinated postpartum (n=108)<br>- Unvaccinated (n=62)	369	less than 12 months (median age at onset illness 2.8 months)	• Timing of vaccination<br>• No vaccination	Pertussis-containing vaccine (dTpa), combined (intramuscular)	21 to 60 months postpartum

12	Pneumococcal	United Kingdom 2000-2001	Intervention Study (Before-After)	3	Vaccinated	0	40, 43 and 96 days	Single cohort study (no comparison cohort)	Pneumococcal capsular polysaccharide, 23-valent PS ('Pneumovax') (i.m.)	2 weeks after vaccination.
13	Polio	Pakistan, Sweden 1977-1979	Cohort Study (Prospective)	40	Vaccinated:<br>• Naturally-exposed (n=31)<br>• Vaccine-primed through childhood vaccines (n=9)	0	1 to 10 months	• Immunization status<br>• Different vaccine types<br>• Different vaccination schedules	• Inactivated, trivalent poliovirus vaccine, parenteral route (s.c.) (n=19)<br>• Live-attenuated, trivalent poliovirus vaccine, enteral route (p.o.) (n=11)<br>• Live-attenuated trivalent poliovirus vaccine, enteral route (p.o.) in combination with a killed whole-cell cholera vaccine, parenteral route (s.c.) (n=10)	4 weeks
14	Rabies	Oman 1997	Case Report	1	Vaccinated as part of post-exposure prophylaxis treatment	1	7 months	No comparator (single case)	Inactivated, human diploid cell vaccine, parenteral (i.m.), and as part of post-exposure prophylaxis also Human Rabies Immune Globulin (HRIG) (i.m.)	19 days (April 20 to May 9, 1997)
15	Rotavirus	United States 1992-1994	Randomized controlled trial	32	Vaccinated (n=21)<br>Placebo (n=11)	30	median of 14 days	• Different serotypes<br>• Placebo: derived from uninfected tissue, oral	Live-attenuated, human-rhesus reassortant rotavirus vaccine (RRV), enteral (p.o.), serotypes:<br>• Monovalent (n=11)<br>• Tetravalent (n=10)	Duration of breastfeeding, up to 6 months.
16	Rubella	United States 1975	Case Report	1	Vaccinated (case)	1	1 day	No comparator (single case)	Live-attenuated vaccine, HPV-77 DE5 strain, parenteral route (s.c.)	15 months
17	Rubella	Sweden 1972	Cohort Study (Prospective)	949	Vaccinated	63	within 4 days	Different strains	Live-attenuated vaccines, parenteral route (s.c.), different strains:<br>• RA 27/3 strain (n=280)<br>• HPV-77 DE-5 strain (n=337)<br>• Cendehill strain (n=332)	≤ 2 months
18	Rubella	United States 1980	Case Report	1	Vaccinated mother of case	1	1 day	No comparator (single case)	Live-attenuated vaccine, HPV-77 DE5 strain, parenteral route (s.c.)	12 months

19	Rubella	United States	Cohort Study (Prospective)	13	Vaccinated	0	2 to 4 days	• Different strains<br>• Different routes (intranasal)	Live-attenuated vaccines, different strains/routes:<br>• RA 27/3 strain, parenteral route (s.c.) (n=4)<br>• RA 27/3 strain, intranasal (i.n.) (n=4)<br>• HPV-77 DE5 strain, parenteral route (s.c.) (n=5)	≤ 5 months
20	Rubella	United States	Cohort Study (Prospective)	26	Vaccinated:<br>- Lactating, Breastfeeding (n=16)<br>- Lactating, Non-breastfeeding (bottle-feeding) (n=10)	26	2 to 4 days	• Different strains<br>• Different routes (intranasal)	Live-attenuated vaccine, different strains/routes:<br>• RA 27/3 strain, parenteral route (s.c.) (n=9)<br>• RA 27/3 strain, intranasal (i.n.) (n=8)<br>• HPV-77 DE5 strain, parenteral route (s.c.) (n=5)	≤ 5 months
21	Rubella	Japan 2003-2006	Before-After Study (uncontrolled, longitudinal design)	254	Cross-sectional analysis/ Known rubella status (n=2741)<br>Follow-up study/ Vaccinated (n=254)	0	within 4 days (n=234)<br>after 1 month (n=20)	No vaccination	Live-attenuated vaccine, TO-336 strain, parenteral route (s.c.)	≤ 1 month
71	Rubella	Canada 1989-1992	Randomised double-blind placebo-controlled study	546		0	0-12 weeks	Control cohort (saline placebo)	Live-attenuated vaccine, RA27/3 strain, parenteral route (s.c.)	12 months
22	Smallpox	United States 2003	Case Report	1	Infected	1	5 months	No comparator (single case)	Live-attenuated vaccine, vaccinia virus strain, percutaneous route	Until resolution of infant and maternal lesions (approximately one month after onset)
23	Varicella	United States 1999-2001	Cohort Study (Prospective)	12	Vaccinated	12	6 weeks (according to vaccination policy at the time of the study)	Repeated measures<br>Pre- and post-intervention	Live-attenuated vaccine, Oka strain ('VARIVAX'), parenteral route (s.c.)	6 weeks after the second vaccine dose (~10 weeks after the first vaccine dose)
72	Varicella	United States	Case Report	1	Vaccinated	1	3 days		Live-attenuated vaccine, Oka strain ('VARIVAX'), parenteral route (s.c.)	

73	Varicella	0	Case Report	1	Vaccinated	1	1 day		Live-attenuated vaccine, Oka strain ('VARIVAX'), parenteral route (s.c.)	
24	Yellow fever	Brazil 2017	Follow-up Study (Observational)	11	Inadvertently vaccinated (instead of MMR vaccine they received YF vaccine)	11	4 to 8 weeks	Single cohort study (no comparison cohort)	Live-attenuated vaccine, 17DD strain, parenteral route (s.c.)	15-28 days
25	Yellow fever	Sudan 2019	Case Series (Prospective)	8	Vaccinated	8	6 weeks to 8 months	No comparison (case series)	Live-attenuated vaccine, 17DD strain, parenteral route (s.c.)	up to 24 days
26	Yellow fever	Canada, Venezuela 2011	Case Report	1	Vaccinated	1	10 days	Single case	Live-attenuated vaccine, 17DD strain, parenteral route (s.c.)	5 months
27	Yellow fever	Japan 10/2018 to 08/2019	Follow-up Study (Intervention)	1	One case who breastfed her infant once after vaccination	1	NR	Single case description within single cohort	Live-attenuated vaccine, 17D-204 substrain (YF-VAX®), parenteral route (s.c.)	30 days
28	Yellow fever	Brazil 2009	Case Report	1	Vaccinated	1	15 days	Single case	Live-attenuated vaccine, 17DD strain, parenteral route (s.c.)	6 months
29	Yellow fever	Brazil 2009	Case Report	1	Vaccinated	1	13 days	Single case	Live-attenuated vaccine, 17DD strain, parenteral route (s.c.)	14 months
30	COVID-19	United States 2021	Cohort Study (Prospective)	20	Vaccinated	0	median of 26 weeks (range 2 to 56 weeks) at receipt of 3rd dose (approx. 14-15 weeks after first dose)	Single cohort study (no comparison cohort)	• mRNA-based vaccine, mRNA-1273 ('Moderna') (n=3)<br>• mRNA-based vaccine, BNT162b2 ('Pfizer') (n=17)	Approximately 1 week post-3rd mRNA vaccine dose for some participants.

31	COVID-19	United States 2020-2021	Cohort Study (Prospective)	31	Vaccinated	0	NR	Pregnancy/lactation status	• mRNA-based vaccine, mRNA-1273 ('Moderna') (n=66)<br>• mRNA-based vaccine, BNT162b2 ('Pfizer') (n=65)	2 to 5.5 weeks after 2nd dose
32	COVID-19	United States 2020-2021	Cohort Study (Prospective)	7	Vaccinated	0	NR	Single cohort study (no comparison cohort)	• mRNA-based vaccine, mRNA-1273 ('Moderna') (n=3)<br>• mRNA-based vaccine, BNT162b2 ('Pfizer') (n=3)	2 weeks after 2nd dose
33	COVID-19	United States 2020-2022	Cohort Study (Prospective)	7	Vaccinated (n=7)	0	below 6 months	Different anti-COVID-19 vaccines:<br>- mRNA-1273<br>- BNT162b2<br>- Ad26.COV2.S	• mRNA-based vaccine, BNT162b2 ('Pfizer') (n=9)<br>• Heterologues schedule: single dose of vector-based vaccine, Ad26.COV2.S ('Janssen') boosted with single dose of mRNA-based vaccine, mRNA-1273 ('Moderna') (n=1)	4 weeks after 3rd dose
34	COVID-19	United States 2020-2021	Cohort Study (Prospective)	180	Vaccinated	180	mean of 7.5 months	Different anti-COVID-19 vaccines:<br>- mRNA-1273<br>- BNT162b2	• mRNA-based vaccine, mRNA-1273 ('Moderna') (n=52)<br>• mRNA-based vaccine, BNT162b2 ('Pfizer') (n=128)	7 days after each vaccine dose.
35	COVID-19	Mexico 2021	Cohort Study (Prospective)	37	Vaccinated (n=34)<br>Unvaccinated (n=3)	0	0 to 36 months	• Different vaccines<br>• Vaccination versus no vaccination (unvaccinated)	• mRNA-based vaccine, BNT162b2 ('Pfizer') (n=11)<br>• Vector-based vaccine, Ad26.COV2.S ('Janssen') (n=17)<br>• Vector-based vaccine, Ad5-nCoV-S ('CanSino') (n=7)	3 to 6 weeks after receipt of vaccine (unclear how many doses)
36	COVID-19	Spain 2021	Cross-Sectional and observational study (Pilot)	25	Vaccinated (n=10)<br>Infected (n=5)<br>Unvaccinated (prepandemic controls) (n=10)	4	median of 8.25 months (IQR 6.5 to 11)	* Infection cohort<br>* Control cohort (unvaccinated, non-infected)	• mRNA-based vaccine, mRNA-1273 ('Moderna') (n=5)<br>• mRNA-based vaccine, BNT162b2 ('Pfizer') (n=5)	2 weeks after second vaccine dose.
37	COVID-19	Portugal 2020-2021	Cohort Study (Prospective)	14	Vaccinated (n=14)	14	12 to 24 months	Lactation status:<br>- Lactating<br>- Non-lactating	mRNA-based vaccine, BNT162b2 ('Pfizer') (n=24)	1 to 3 weeks after 2nd dose
38	COVID-19	Israel 2020-2021	Cohort Study (Prospective)	16	Vaccinated (n=16)	0	NR	• Infected pregnant cohort<br>• Infected non-pregnant, non-lactating cohort<br>• Vaccinated	• mRNA-based vaccine, mRNA-1273 ('Moderna') (n=134)<br>• mRNA-based vaccine, BNT162b2 ('Pfizer') (n=166)	2 to 8 weeks after 2nd dose

								pregnant cohort<br>• Vaccinated non-pregnant, non-lactating		
39	COVID-19	Italy<br>2020-2021	Cross-sectional study	12	Vaccinated (n=12)	0	median of 233.5 days	* Infection cohort<br>* Vaccination during pregnancy cohort<br>* Vaccination during lactation cohort	mRNA-based vaccine, BNT162b2 ('Pfizer')	Previously infected: 2 months post-infection; Vaccine received during pregnancy: 2 months post-vaccination; Vaccine received postpartum: 10 days post-vaccination.
40	COVID-19	Portugal<br>2021	Case Report	1	Vaccinated	1	16 months	Single case(s)	Adenovirus-vectored vaccine, ChAdOx1 ('AstraZeneca')	Not applicable.
41	COVID-19	United States<br>2022	Case Report	1	Vaccinated	0	8 months	Single case(s)	mRNA-based vaccine, BNT162b2 ('Pfizer')	Not applicable.
42	COVID-19	Spain<br>2021	Cohort Study (Prospective)	33	Vaccinated	0	mean of 17.5 months (SD 10.1)	Single cohort study (no comparison cohort)	mRNA-based vaccine, BNT162b2 ('Pfizer')	4 weeks after the second vaccine dose
43	COVID-19	United States<br>2020-2021	Cohort Study (Prospective)	48	Vaccinated	8	median of 4.7 months (range 0.1 to 17.2)	• Pre- and post-intervention<br>• Dose-dependent response<br>• Different mRNA-based vaccines	• mRNA-based vaccine, BNT162b2 ('Pfizer') (n=27)<br>• mRNA-based vaccine, mRNA-1273 ('Moderna') (n=21)	Follow-up sample collection: 4-10 weeks after 2nd vaccine dose.

44	COVID-19	United States 2020-2021	Cohort Study (Prospective)	7	Vaccinated	0	1 month to 3 years	Single cohort study (no comparison cohort)	• mRNA-based vaccine, mRNA-1273 ('Moderna') (n=2)<br>• mRNA-based vaccine, BNT162b2 ('Pfizer') (n=5)	Pre-vaccination, up to 2 days after vaccine dose 2.
45	COVID-19	Portugal 2020-2021	Cohort Study (Prospective)	23	Vaccinated (n=23)<br>mRNA-1273 (n=2)<br>BNT162b2 (n=21)	0	range 3 to 23 months	Lactation status:<br>- Lactating<br>- Non-lactating	• mRNA-based vaccine, mRNA-1273 ('Moderna') (n=4)<br>• mRNA-based vaccine, BNT162b2 ('Pfizer') (n=41)	~2.5 weeks after 2nd dose.
46	COVID-19	United States 2020-2021	Cohort Study (Prospective)	31	Vaccinated (n=31)	0	median of 7.3 months	Pregnancy status	• mRNA-based vaccine, BNT162b2 ('Pfizer') (n=65)<br>• mRNA-based vaccine, mRNA-1273 ('Moderna') (n=66)	
47	COVID-19	United States 2021	Cohort Study (Prospective)	8	Vaccinated (n=8)	0	third (booster) dose approximately 3 to 4 months	Pregnancy status:<br>- Pregnant<br>- Lactating	mRNA-based vaccine, BNT162b2 ('Pfizer')	
48	COVID-19	Turkey 2021	Case Series	2	Lactating, vaccinated (n=2)	0	5 and 15 months	Single case(s)	Whole inactivated virus vaccine,CoronaVac ('Sinovac')	Follow-up in the 4th week: blood tests including thyroid function tests
49	COVID-19	Poland 2020-2021	Cohort Study (Prospective)	60	Lactating, vaccinated (n=32)<br>Lactating, unvaccinated (n=28)	32	mean of 33.3 months	Control cohort (unvaccinated)	mRNA-based vaccine, BNT162b2 ('Pfizer')	6 to 7 weeks after first vaccine dose (43 ± 4 days)
50	COVID-19	Netherlands 2020	Cohort Study (Prospective)	46	Lactating, vaccinated (n=28)<br>Lactating, infected (n=18)	0	6 to 12 months	Infection cohort	mRNA-based vaccine, BNT162b2 ('Pfizer')	70 days
51	COVID-19	Netherlands 2020	Cohort Study (Prospective)	26	Lactating, vaccinated	0	median of 7 months (IQR 5 to 9)	Single cohort study (no comparison cohort)	mRNA-based vaccine, BNT162b2 ('Pfizer')	The 20 participants who received both vaccine doses had a median follow-up starting from receipt of the first

										vaccine dose ranging from 35 to 45 days.
52	COVID-19	Netherlands 2021	Cohort Study (Prospective)	134	Lactating, vaccinated	0	median of 39.3 and 40.4 weeks	Different anti-COVID-19 vaccines:<br>- mRNA-1273<br>- BNT162b2<br>- Ad26.COV2.S<br>- AZD1222	• mRNA-based vaccine, BNT162b2 ('Pfizer') (n=10790)<br>• mRNA-based vaccine, mRNA-1273 ('Moderna') (n=6592)<br>• Adenovirus-vectored vaccine, Ad26.COV2.S ('Janssen') (n=49)	70 days
53	COVID-19	United States 2021	Cohort Study (Prospective)	6815	Lactating, vaccinated (n=6815)	6815	NR	Pregnancy status:<br>- Pregnant<br>- Lactating<br>- Non-pregnant, non-lactating age-matched controls	• mRNA-based vaccine, BNT162b2 ('Pfizer') (n=28)<br>• mRNA-based vaccine, mRNA-1273 ('Moderna') (n=43)<br>• Adenovirus-vectored vaccine, Ad26.COV2.S ('Janssen') (n=25)	1 day after each dose
54	COVID-19	United States 2020-2021	Cohort Study (Prospective)	13	Lactating, vaccinated (n=13)	0	3 weeks	Pregnancy status:<br>- Lactating<br>- Non-pregnant, non-lactating age-matched controls	• mRNA-based vaccine, BNT162b2 ('Pfizer') (n=8)<br>• mRNA-based vaccine, mRNA-1273 ('Moderna') (n=19)	18 weeks after 2nd dose
55	COVID-19	Singapore 2021	Cohort Study (Prospective)	23	Lactating, vaccinated (n=14)<br>Lactating, unvaccinated (n=9)	14	mean of 9 months (SD 3.7)	* Infection cohort<br>* Control cohort (unvaccinated, non-infected)	mRNA-based vaccine, BNT162b2 ('Pfizer')	4-6 weeks
56	COVID-19	Singapore 2021	Cohort Study (Prospective)	88	Lactating, vaccinated	67	mean of 10 months (SD 5.2)	Single cohort study (no comparison cohort)	mRNA-based vaccine, BNT162b2 ('Pfizer')	28 days after second vaccin dose
57	COVID-19	Singapore 2021	Cohort Study (Prospective)	46	Lactating, vaccinated	0	mean of 13.5 months	Single cohort study (no comparison cohort)	mRNA-based vaccine, BNT162b2 ('Pfizer')	4-6 weeks
58	COVID-19	Netherlands 2021	Case Series	2	Lactating, vaccinated	0	4 and 7 months	Homologues vaccination schedule	Heterologues prime-booster schedule: first dose of the adenovirus-vectored vaccine ChAdOx1 ('AstraZeneca'), boosted with a single dose of an mRNA-based vaccine BNT162b2 ('Pfizer')	99-100 days after first dose of AZD1222 vaccine

									(n=1) and mRNA-1273 ('Moderna') (n=1)	
59	COVID-19	United States	Cohort Study (Prospective)	30	Lactating, vaccinated	25	mean of 239 days (range 7 to 651)	Single cohort study (no comparison cohort)	• mRNA-based vaccine, BNT162b2 ('Pfizer') (n=20)<br>• mRNA-based vaccine, mRNA-1273 ('Moderna') (n=10)	3 weeks after 2nd dose
60	COVID-19	United States 2020-2021	Cohort Study (Prospective)	27	Lactating, vaccinated (n=27)	0	NR	Single cohort study (no comparison cohort)	• mRNA-based vaccine, BNT162b2 ('Pfizer') (n=26)<br>• mRNA-based vaccine, mRNA-1273 ('Moderna') (n=3)<br>• Vector-based vaccine, Ad26.COV2.S ('Janssen') (n=1)	6 months
61	COVID-19	Israel 2020-2021	Cohort Study (Prospective)	20	Lactating, vaccinated	0	mean of 154 days (range 68 to 382)	Control cohort (unvaccinated)	mRNA-based vaccine, BNT162b2 ('Pfizer')	14 days following second vaccine dose
62	COVID-19	Israel 2020-2021	Cohort Study (Prospective)	61	Lactating, vaccinated	21	NR	Single cohort study (no comparison cohort)	mRNA-based vaccine, BNT162b2 ('Pfizer')	unclear
63	COVID-19	Spain 2021-2022	Cohort Study (Prospective)	86	Lactating, vaccinated	86	mean of 11 to 14 months	Different anti-COVID-19 vaccines:<br>- mRNA-1273<br>- BNT162b2<br>- ChAdOx1	• mRNA-based vaccine, BNT162b2 ('Pfizer') (n=34)<br>• mRNA-based vaccine, mRNA-1273 ('Moderna') (n=20)<br>• Adenovirus-vectored vaccine, ChAdOx1 ('AstraZeneca') (n=32)	Up to 30 days after the second dose
64	COVID-19	United States 2020-2021	Cohort Study (Prospective)	34	Lactating, vaccinated (n=34)	40	mean of 5.2 months (SD 6.2)	Single cohort study (no comparison cohort)	• mRNA-based vaccine, BNT162b2 ('Pfizer') (n=20)<br>• mRNA-based vaccine, mRNA-1273 ('Moderna') (n=13)	6 months
65	COVID-19	Romania 2021	Cohort Study (Prospective)	26	Lactating, vaccinated	0	2 to 35 months	Single cohort study (no comparison cohort)	• mRNA-based vaccine, mRNA-1273 ('Moderna') (n=3)<br>• mRNA-based vaccine, BNT162b2 ('Pfizer') (n=23)	60 days after the second vaccine dose

66	COVID-19	United States 2020-2021	Cohort Study (Prospective)	21	Lactating, vaccinated	0	mean of 6.8 months (SD 4.8)	Single cohort study (no comparison cohort)	• mRNA-based vaccine, mRNA-1273 ('Moderna') (n=7)<br>• mRNA-based vaccine, BNT162b2 ('Pfizer') (n=14)	7-10 days after receipt of the second vaccine dose.
67	COVID-19	United States 2020-2021	Cohort Study (Prospective)	75	Lactating, vaccinated (n=30)<br>Lactating, infected (n=45)	0	mean of 7.5 months (range 0.5 to 23.4)	Infection cohort	• mRNA-based vaccine, mRNA-1273 ('Moderna') (n=18)<br>• mRNA-based vaccine, BNT162b2 ('Pfizer') (n=12)	18 days after second vaccine dose; 28-42 days after PCR-confirmed COVID-19 diagnosis.

Table S4. Adverse events in infants following vaccination of lactating mothers

Caption Table S4. Each row in the table corresponds to a scientific research paper from the literature review. The unique key number in the Key column identifies each paper, and the full bibliographic reference for each key number is located at the end of this Supplementary Materials file under 'Key to reference'. Abbreviations: AE, adverse event; NS, nasal secretions; HM, human milk; s.c., subcutaneous, i.m., intramuscular; NR, not reported; YEL-AND, yellow fever vaccine-associated neurotropic disease; YF, yellow fever; mRNA, messenger ribonucleic acid; PCR, polymerase chain reaction; NT, not tested.

Key	Population	Intervention/ comparison	No. doses	Infant Safety Monitoring Method	Vaccine shedding summary (see table 2)	Adverse events in infants
7	Lactating	Live influenza (nasal) + placebo (i.m.)<br><br>Inactivated influenza (i.m.) + placebo (nasal)	One	**Active Monitoring**<br>• Solicited AEs, 10 days | Unsolicited AEs, 28 days | Recorded by parents using memory aids<br>• ILI symptoms | 28 days | If present, clinical visit<br>• Viral shedding / transmission |0, 2, 9 days | Analysis viral presence (maternal NS, HM, infant NS)	Viral shedding: >48%<br>Transmission: 1%<br>Clinical signs: none	• Irritability/Fussiness: 59.7% (p = 0.02)<br>• Other symptoms not individually reported but no group differences
	Lactating	Inactivated influenza (i.m.) + placebo (nasal)	One	^<br>• Control group for vaccine shedding	Viral shedding: 0<br>Transmission: 0<br>Clinical signs: none	• Irritability/Fussiness: 44.8%
15	Lactating	Live rotavirus (oral)	One	**Active Monitoring**<br>• Viral shedding / transmission | 1x/wk for 2 weeks | Analysis for viral presence	Viral shedding: 1/55 (2%)<br>Transmission: 0/39<br>Clinical signs: NR	Not studied.
	Lactating	Placebo (oral)	One	^<br>• Control group for vaccine shedding	Viral shedding: 0/30<br>Transmission: 0/21<br>Clinical signs: NR	Not studied.
16	Case report of maternal AE	Live rubella, HPV-77 DE5 (s.c.)	One	**Case report**<br>• Clinical observations of infant due to maternal AE (adverse reactions and viral detection in human milk)	Viral shedding: yes (NC)<br>Transmission: yes<br>Clinical signs: none	Case report: no symptoms.
17	Lactating	Live rubella, RA27/3, HPV-77 DE5, Cendehill (s.c.)	One	**Passive monitoring**<br>• Unsolicited AE | 8 months| routine child examination during postpartum visit and serology testing for rubella	Viral shedding: NT<br>Transmission: NT<br>Clinical signs: 0/63	SAE: 0/63
18	Case report on infant AE	Live rubella, HPV-77 DE5 (s.c.)	One	**Case report**<br>• Clinical observations of infant with rubella-like symptoms after maternal postpartum vaccination	Viral shedding: negative<br>Transmission: negative<br>Clinical signs: yes	Case report: rash, vomiting, leukopenia (within 4 weeks,

						transient); at one year normal growth and develoment
19	Lactating	Live rubella, RA 27/3 (s.c.)	One	**Active monitoring**<br>Viral shedding/transmission | range 8 - 20 weeks | viral detection in maternal HM and NS	Viral shedding: 100%<br>Transmission: NT<br>Clinical signs: NR	Not studied.
	Lactating	Live rubella, RA 27/3 (nasal)	One	^	Viral shedding: 100%<br>Transmission: NT<br>Clinical signs: NR	Not studied.
	Lactating	Live rubella, HPV-77 DE5 (s.c.)	One	^	Viral shedding: 20%<br>Transmission: NT<br>Clinical signs: NR	Not studied.
20	Lactating, breastfeeding	Live rubella (s.c. or nasal)	One	**Active monitoring**<br>Viral shedding/transmission | range 8 - 20 weeks | viral detection in infant NS, maternal NS and HM, clinical signs (symptoms not specified, rubella serology)	Viral shedding: HM 11/16 (69%), NS 9/16 (56%)<br>Transmission: 9/16 (56%)<br>Clinical signs: symptoms 0/16, rubella serology 4/16	Rubella-like symptoms (unspecified): 0/16
	Lactating, non-breastfeeding	Live rubella (s.c. or nasal)	One	^	Viral shedding: HM NT, NS 5/10 (50%)<br>Transmission: 0/10<br>Clinical signs: 0/10	Rubella-like symptoms (unspecified): 0/10
22	Case report on infant AE	Live vaccinia, (percutaneous)	One	Report on vaccinia transmission to infant.	Viral shedding: yes<br>Transmission: yes<br>Clinical signs: yes	Not studied.
23	Lactating	Live varicella (s.c.)	Two	**Active + Passive monitoring**<br>• Rash | 6 weeks after each dose | Contact at specific timepoints, spontaneous report by parents<br>• Viral detection after each dose |infant serum at 4 wk | HM at 3 wk | Rash lesion (if present), examination and PCR-analysis	Viral shedding: 0/217<br>Transmission: 0/6 (6 missing)<br>Clinical signs: 0/12	Not studied.
72	Case report on infant AE	Live varicella (s.c.)	NR	Report on varicella vaccine transmission to infant.	Viral shedding: NT<br>Transmission: infant rash (C)<br>Clinical signs: yes	Case: Varicella disease, mild course, vesicular rash, no hospitalization.
73	Case report on infant AE	Live varicella (s.c.)	NR	Report on varicella vaccine transmission to infant.	Viral shedding: maternal rash (C)<br>Transmission: infant rash	Case: Extensive vesicular rash and fever requiring hospitalization.

					(C)<br>Clinical signs: serology	
24	Lactating	Live YF (sc)	One	**Active monitoring**<br>• Symptoms (YF-like, not specified)| 28 days | Clinical pediatric examination<br>• Shedding/transmission |8,10,15 days| HM PCR-analysis	Viral shedding: 0/10<br>Transmission: NT<br>Symptoms: 0/11	YF-like symptoms: 0/11
25	Case series on infant AE	Live YF (sc)	One	Report on clinical assessments of infants with YF-like illness after maternal vaccination.	Viral shedding: 6/8<br>Transmission: NT<br>Symptoms: yes	Cases: Yellow fever like illness in infants (fever, diarrhea, jaundice, vomiting, and/or skin rashes)
26	Case report on infant AE	Live YF (sc)	One	Report on YEL-AND-like illness in infant after maternal vaccination.	Viral shedding: NT<br>Transmission: no<br>Clinical signs: meningoencephalitis and YF-serology	Case: YEL-AND like illness (meningoencephalitis and YF-serology; vaccine strain not confirmed)
27	Healthy subjects aged 6 months or above	Live YF (sc)	One	Report on clinical observation of a single infant after continued breastfeeding following maternal vaccination	Viral shedding: NT<br>Transmission: NT<br>Clinical signs: none	Case: no symptoms.
28	Case report on infant AE	Live YF (sc)	One	Report on YEL-AND in infant after maternal vaccination.	Viral shedding: NT<br>Transmission: yes (C)<br>Clinical signs: meningo-encephalitis and YF-serology	Case: YEL-AND (Yellow fever vaccine associated neurotropic disease)
29	Case report on infant AE	Live YF (sc)	One	Report on YEL-AND-like illness in infant after maternal vaccination.	Viral shedding: NT<br>Transmission: NT<br>Clinical signs: meningo-encephalitis and YF-serology	Case: YEL-AND-like illness (meningoencephalitis and YF-serology; vaccine strain not confirmed)
34	Lactating, vaccinated	mRNA-based vaccine	First, Second	**Active Monitoring**<br>Prespecified (4 local, 14 systemic incl 2 lactation-related) | 7 days after each dose | Questionnaire and intervieuw	Vaccine excretion:	
36	Lactating, vaccinated	mRNA-based vaccine	Two	**Active Monitoring**<br>HM-altered microbiota infant | timeframe NR | stool analysis and colonic-fermentation simulation	NT	No alterations in microbiota of ... infants.

37	Lactating, vaccinated	mRNA-based vaccine	First, Second	**Passive monitoring**<br>Infant behaviour | timeframe NR | Spontaneous parent reporting	NT	
40	Case report on infant AE	Viral vectored vaccine	First	Report on rare AE in infant after maternal vaccination.	NT	Case: cutaneous reaction
43	Lactating, vaccinated	mRNA-based vaccine	First, Second	**Active Monitoring**<br>Vaccine excretion | from 24h after dose 1 to 2wk after dose 2 | HM analysis on change in PEG concentration	Vaccine excretion: 0/13<br>Clinical signs: NR	Not studied.
44	Lactating, vaccinated	mRNA-based vaccine	Two	**Active Monitoring**<br>Vaccine excretion | at least 24h after each dose | Analysis of HM on mRNA detection	Vaccine excretion: no<br>Clinical signs: not studied	Not studied.
49	Lactating, vaccinated	mRNA-based vaccine	First, Second	**Active monitoring**<br>Prespecified (12 AEs) | timeframe NR, after each dose| Spontaneous parent reporting, questionnaire	NT	AE in infant (N=32)<br>- After 1st dose: 1 (3.1%) (in one infant behaviour change and increased tearfulness)<br>- After 2nd dose: 1 (3.1%) (in one infant 'other' AE than pre-specified AEs)
53	Lactating, vaccinated	mRNA-based vaccine or viral vectored vaccine	First, Second	**Passive monitoring**<br>Unsolicited AEs | 1 day | Spontaneous parent-reported survey data	NT	N=6815<br>• Concerns about infant:<br>208 (3%) after 1st vaccine dose<br>267 (4.4%) after 2nd dose<br>• Interruption breastfeeding:<br>1st dose: 155/6815<br>2nd dose: 130/6056
55	Lactating, vaccinated	Subunit (mRNA)	Two	**Active monitoring**<br>• Vaccine excretion | time |HM analysis on vaccine mRNA detection<br>• Solicited AEs, SAEs | 28 days after each dose | Parent reporting, questionnaire	Excretion of mRNA: <10%<br>Transmission: not studied.<br>Symptoms: 0/12	SAEs: 0 / 14<br>Continued breastfed: 12/14<br>AEs among breastfed infants: 0/12
56	Lactating, vaccinated	Subunit (mRNA)	Two	**Passive monitoring**<br>Infants continued breastfeed <72h after maternal vaccination | 28 days after 2nd dose | Spontaneous parent reporting	NT	AEs among continued breastfed infants: 0/ 67

59	Lactating, vaccinated	mRNA-based vaccine	First, Second	**Active monitoring**<br>Solicited AEs | time after each dose | Parent reporting, questionnaire	NT	Questionnaire completed by 25 after each dose (N=30).<br>No. AE, dose 1 | dose 2<br>Fever: 2 (7%) | 0<br>Runny nose: 1 (3%) | 2 (7%)<br>No side effects: 22 (73%) | 22 (73%)
63	Lactating, vaccinated	mRNA-based vaccine	First, Second	**Active monitoring**<br>Solicited AEs, SAEs | 28 days after 2nd dose | Questionnaire, parent reporting	NT	SAE: 0/54<br>Skin reaction: 1/54 | 0/51<br>Fever: 1/54 | 5/51<br>Irritability and insomnia: 2/54 | 2/51
	Lactating, vaccinated	Viral-vectored vaccine	First, Second	**Active monitoring**<br>Solicited AEs, SAEs | 28 days after 2nd dose | Questionnaire, parent reporting	NT	SAEs: 0/ 32<br>AEs dose 1 | dose 2<br>Skin reaction: 2/32 | 0/29<br>Fever: 0/32 | 2/29<br>Irritability and insomnia: 5/32 | 4/29
69	Lactating, vaccinated	mRNA-based vaccine	Two	**Active monitoring**<br>• Vaccine excretion | 1 wk |Maternal samples (HM and serum), and infant serum analysis on mRNA detection	Vaccine excretion: HM 5/309 (2%), serum 20/74 (27%)<br>Transmission: 0/5<br>Clinical signs: NR	Not studied.
70	Lactating, vaccinated	mRNA-based vaccine	Two	**Active monitoring**<br>• Vaccine excretion | timeframe|HM analysis on mRNA detection	Vaccine excretion: 0/30<br>Transmission: not studied.<br>Clinical signs: NR	Not studied.
	Lactating, infected	PCR-confirmed SARS-CoV-2	NA	^	Viral excretion: 0/47<br>Transmission: NT<br>Clinical signs: NR	Not studied.

References

1. NHLBI. Study Quality Assessment Tools. National Heart, Lung, and Blood Institute (NHLBI). 2013. Accessed October 14, 2024. https://www.nhlbi.nih.gov/health-topics/study-quality-assessment-tools

2. Moola S, Munn Z, Tufanaru C, et al. Chapter 7: Systematic reviews of etiology and risk. In: *JBI Manual for Evidence Synthesis*. ; 2020. https://synthesismanual.jbi.global

3. Sterne JAC, Savović J, Page MJ, et al. RoB 2: a revised tool for assessing risk of bias in randomised trials. *BMJ*. 2019;366:l4898. doi:10.1136/bmj.l4898

4. Sterne JA, Hernán MA, Reeves BC, et al. ROBINS-I: a tool for assessing risk of bias in non-randomised studies of interventions. *BMJ*. 2016;355:i4919. doi:10.1136/bmj.i4919

Key to reference

Studies included in the literature review for the narrative data synthesis are identified in the following supplementary tables by a unique key number. Below is the bibliographic list of included studies with the corresponding key number.

Key	Reference
Key 1	Clemens, J. D., D. A. Sack, J. R. Harris, M. R. Khan, J. Chakraborty, S. Chowdhury, M. R. Rao, et al. 'Breast Feeding and the Risk of Severe Cholera in Rural Bangladeshi Children'. *American Journal of Epidemiology* 131, no. 3 (1 March 1990): 400–411. https://doi.org/10.1093/oxfordjournals.aje.a115515.
Key 2	Clemens, J. D., D. A. Sack, J. Chakraborty, M. R. Rao, F. Ahmed, F. Van Loon, M. R. Khan, et al. 'Field Trial of Oral Cholera Vaccines in Bangladesh: Evaluation of Anti-Bacterial and Anti-Toxic Breast-Milk Immunity in Response to Ingestion of the Vaccines'. *Vaccine* 8, no. 5 (January 1990): 469–72. https://doi.org/10.1016/0264-410X(90)90248-K.
Key 3	Hahn-Zoric, M., B. Carlsson, F. Jalil, L. Mellander, R. Germanier, and L. A. Hanson. 'The Influence on the Secretory IgA Antibody Levels in Lactating Women of Oral Typhoid and Parenteral Cholera Vaccines given Alone or in Combination'. *Scandinavian Journal of Infectious Diseases* 21, no. 4 (1989): 421–26. https://doi.org/10.3109/00365548909167447.
Key 4	Mascart-Lemone, F., B. Carlsson, F. Jalil, M. Hahn-Zoric, J. Duchateau, and L. A. Hanson. 'Polymeric and Monomeric IgA Response in Serum and Milk after Parenteral Cholera and Oral Typhoid Vaccination'. *Scandinavian Journal of Immunology* 28, no. 4 (October 1988): 443–48. https://doi.org/10.1111/j.1365-3083.1988.tb01474.x.

Key 5	Merson, M. H., R. E. Black, D. A. Sack, A. M. Svennerholm, and J. Holmgren. ‘Maternal Cholera Immunisation and Scecretory IgA in Breast Milk’. *Lancet (London, England)* 1, no. 8174 (26 April 1980): 931–32. https://doi.org/10.1016/s0140-6736(80)90860-0.
Key 6	Svennerholm, A.-M., L. Å. Hanson, J. Holmgren, B. S. Lindblad, B. Nilsson, and F. Quereshi. ‘Different Secretory Immunoglobulin A Antibody Responses to Cholera Vaccination in Swedish and Pakistani Women’. *Infection and Immunity* 30, no. 2 (November 1980): 427–30. https://doi.org/10.1128/iai.30.2.427-430.1980.
Key 7	Brady, Rebecca C., Lisa A. Jackson, Sharon E. Frey, Andi L. Shane, Emmanuel B. Walter, Geeta K. Swamy, Elizabeth P. Schlaudecker, et al. ‘Randomized Trial Comparing the Safety and Antibody Responses to Live Attenuated versus Inactivated Influenza Vaccine When Administered to Breastfeeding Women’. *Vaccine* 36, no. 31 (25 July 2018): 4663–71. https://doi.org/10.1016/j.vaccine.2018.06.036.
Key 8	Demers-Mathieu, Veronique, Ciera DaPra, and Elena Medo. ‘Influenza Vaccine Associated with the Gene Expression of T Cell Surface Markers in Human Milk’. *Breastfeeding Medicine: The Official Journal of the Academy of Breastfeeding Medicine* 17, no. 3 (March 2022): 218–25. https://doi.org/10.1089/bfm.2021.0186.
Key 9	De Schutter, Sara, Kirsten Maertens, Lesley Baerts, Ingrid De Meester, Pierre Van Damme, and Elke Leuridan. ‘Quantification of Vaccine-Induced Antipertussis Toxin Secretory IgA Antibodies in Breast Milk: Comparison of Different Vaccination Strategies in Women’. *The Pediatric Infectious Disease Journal* 34, no. 6 (June 2015): e149-152. https://doi.org/10.1097/INF.0000000000000675.
Key 10	Orije, Marjolein R P, Ynke Larivière, Sereina A Herzog, Ludo M Mahieu, Pierre Van Damme, Elke Leuridan, and Kirsten Maertens. ‘Breast Milk Antibody Levels in Tdap-Vaccinated Women After Preterm Delivery’. *Clinical Infectious Diseases* 73, no. 6 (15 September 2021): e1305–13. https://doi.org/10.1093/cid/ciab260.
Key 11	Rowe, Stacey L., Ee Laine Tay, Lucinda J. Franklin, Nicola Stephens, Robert S. Ware, Marlena C. Kaczmarek, Rosemary A. Lester, and Stephen B. Lambert. ‘Effectiveness of Parental Cocooning as a Vaccination Strategy to Prevent Pertussis Infection in Infants: A Case-Control Study’. *Vaccine* 36, no. 15 (5 April 2018): 2012–19. https://doi.org/10.1016/j.vaccine.2018.02.094.
Key 12	Finn, Adam, Qibo Zhang, Lynn Seymour, Claudine Fasching, Emily Pettitt, and Edward N. Janoff. ‘Induction of Functional Secretory IgA Responses in Breast Milk, by Pneumococcal Capsular Polysaccharides’. *The Journal of Infectious Diseases* 186, no. 10 (15 November 2002): 1422–29. https://doi.org/10.1086/344356.
Key 13	Svennerholm, A. M., L. A. Hanson, J. Holmgren, F. Jalil, B. S. Lindblad, S. R. Khan, A. Nilsson, and B. Svennerholm. ‘Antibody Responses to Live and Killed Poliovirus Vaccines in the Milk of Pakistani and Swedish Women’. *The Journal of Infectious Diseases* 143, no. 5 (May 1981): 707–11. https://doi.org/10.1093/infdis/143.5.707.
Key 14	Scrimgeour, E. M., and F. R. Mehta. ‘Rabies in Oman: Failed Postexposure Vaccination in a Lactating Woman Bitten by a Fox’. *International Journal of Infectious Diseases: IJID: Official Publication of the International Society for Infectious Diseases* 5, no. 3 (2001): 160–62. https://doi.org/10.1016/s1201-9712(01)90092-x.

Key 15	Pickering, Larry K., Ardythe L. Morrow, Ismael Herrera, Miguel O'Ryan, Mary K. Estes, Stephen E. Guilliams, Laurie Jackson, Shelly Carter-Campbell, and David O. Matson. 'Effect of Maternal Rotavirus Immunization on Milk and Serum Antibody Titers'. *The Journal of Infectious Diseases* 172, no. 3 (1 September 1995): 723–28. https://doi.org/10.1093/infdis/172.3.723.
Key 16	Buimovici-Klein, E., R. L. Hite, T. Byrne, and L. Z. Cooper. 'Isolation of Rubella Virus in Milk after Postpartum Immunization'. *The Journal of Pediatrics* 91, no. 6 (December 1977): 939–41. https://doi.org/10.1016/s0022-3476(77)80894-9.
Key 17	Grillner, L., C. E. Hedström, H. Bergström, L. Forssman, A. Rignér, and E. Lycke. 'Vaccination against Rubella of Newly Delivered Women'. *Scandinavian Journal of Infectious Diseases* 5, no. 4 (1973): 237–41. https://doi.org/10.3109/inf.1973.5.issue-4.01.
Key 18	Landes, R. D., J. W. Bass, E. W. Millunchick, and W. J. Oetgen. 'Neonatal Rubella Following Postpartum Maternal Immunization'. *The Journal of Pediatrics* 97, no. 3 (September 1980): 465–67. https://doi.org/10.1016/s0022-3476(80)80207-1.
Key 19	Losonsky, G. A., J. M. Fishaut, J. Strussenberg, and P. L. Ogra. 'Effect of Immunization against Rubella on Lactation Products. I. Development and Characterization of Specific Immunologic Reactivity in Breast Milk'. *The Journal of Infectious Diseases* 145, no. 5 (May 1982): 654–60. https://doi.org/10.1093/infdis/145.2.654.
Key 20	Losonsky, Genevieve A., J. Mark Fishaut, Joanne Strussenberg, and Pearay L. Ogra. 'Effect of Immunization Against Rubella on Lactation Products. II. Maternal-Neonatal Interactions'. *The Journal of Infectious Diseases* 145, no. 5 (1 May 1982): 661–66. https://doi.org/10.1093/infdis/145.2.661.
Key 21	Okuda, Mika, Michiko Yamanaka, Tsuneo Takahashi, Hiroshi Ishikawa, Masaya Endoh, and Fumiki Hirahara. 'Positive Rates for Rubella Antibody in Pregnant Women and Benefit of Post-Partum Vaccination in a Japanese Perinatal Center'. *The Journal of Obstetrics and Gynaecology Research* 34, no. 2 (April 2008): 168–73. https://doi.org/10.1111/j.1447-0756.2007.00689.x.
Key 22	Garde, Vinaya, David Harper, and Mary P. Fairchok. 'Tertiary Contact Vaccinia in a Breastfeeding Infant'. *JAMA* 291, no. 6 (11 February 2004): 725–27. https://doi.org/10.1001/jama.291.6.725.
Key 23	Bohlke, Kari, Karin Galil, Lisa A. Jackson, D. Scott Schmid, Pat Starkovich, Vladimir N. Loparev, and Jane F. Seward. 'Postpartum Varicella Vaccination: Is the Vaccine Virus Excreted in Breast Milk?' *Obstetrics and Gynecology* 102, no. 5 Pt 1 (November 2003): 970–77. https://doi.org/10.1016/s0029-7844(03)00860-3.
Key 24	Fernandes, Eder Gatti, Juliana Silva Nogueira, Victor Bertollo Gomes Porto, and Helena Keico Sato. 'The Search for Yellow Fever Virus Vaccine in Breast Milk of Inadvertently Vaccinated Women in Brazil'. *Revista Do Instituto De Medicina Tropical De Sao Paulo* 62 (2020): e33. https://doi.org/10.1590/s1678-9946202062033.
Key 25	Hassan, Tarteel, Razan A. Bashir, Dina N. Abdelrahman, Hassan Madni, Abdel Rahim M El Hussein, Isam M. Elkidir, and Khalid A. Enan. 'Transmission of Yellow Fever Vaccine Virus from Breast Feeding Mothers to Their Infants: Reporting of Yellow Fever Virus (YFV) RNA Detection in Milk Specimens'. *F1000Research* 11 (2022): 76. https://doi.org/10.12688/f1000research.74576.3.

Key 26	Kuhn, Susan, Loreto Twele-Montecinos, Judy MacDonald, Patricia Webster, and Barbara Law. 'Case Report: Probable Transmission of Vaccine Strain of Yellow Fever Virus to an Infant via Breast Milk'. *CMAJ: Canadian Medical Association Journal = Journal de l'Association Medicale Canadienne* 183, no. 4 (8 March 2011): E243-245. https://doi.org/10.1503/cmaj.100619.
Key 27	Miyazato, Yusuke, Mari Terada, Mugen Ujiie, Sho Saito, Akinari Moriya, Masao Ando, and Norio Ohmagari. 'A Nationwide Prospective Cohort Study on Safety of the 17D-204 Yellow Fever Vaccine during a Vaccine Shortage in Japan'. *Journal of Travel Medicine* 30, no. 2 (1 March 2023): taac070. https://doi.org/10.1093/jtm/taac070.
Key 28	Couto, Salomão, Schermann, Mohrdieck, Suzuki A, Carvalho, De Assis, Araújo, and Flannery. 'Transmission of Yellow Fever Vaccine Virus through Breast-Feeding - Brazil, 2009'. *Morbidity and Mortality Weekly Report* 59, no. 5 (12 February 2010): 130–32.
Key 29	Traiber, Cristiane, Priscila Coelho Amaral, Valéria Raymundo Fonteles Ritter, and Annelise Winge. 'Infant Meningoencephalitis Probably Caused by Yellow Fever Vaccine Virus Transmitted via Breastmilk'. *Jornal de Pediatria* 87, no. 3 (1 April 2011): 269–72. https://doi.org/10.2223/JPED.2067.
Key 30	Armistead, Blair, Yonghou Jiang, Marc Carlson, Emily S. Ford, Saumya Jani, John Houck, Xia Wu, et al. 'Spike-Specific T Cells Are Enriched in Breastmilk Following SARS-CoV-2 mRNA Vaccination'. *Mucosal Immunology* 16, no. 1 (1 February 2023): 39–49. https://doi.org/10.1016/j.mucimm.2023.01.003.
Key 31	Atyeo, Caroline, Elizabeth A. DeRiso, Christine Davis, Evan A. Bordt, Rose M. De Guzman, Lydia L. Shook, Lael M. Yonker, et al. 'COVID-19 mRNA Vaccines Drive Differential Antibody Fc-Functional Profiles in Pregnant, Lactating, and Nonpregnant Women'. *Science Translational Medicine* 13, no. 617 (19 October 2021): eabi8631. https://doi.org/10.3389/fimmu.2021.777103.
Key 32	Baird, Jill K., Shawn M. Jensen, Walter J. Urba, Bernard A. Fox, and Jason R. Baird. 'SARS-CoV-2 Antibodies Detected in Mother's Milk Post-Vaccination'. *Journal of Human Lactation* 37, no. 3 (1 August 2021): 492–98. https://doi.org/10.1177/08903344211030168.
Key 33	Bender, Jeffrey M., Yesun Lee, Wesley A. Cheng, Carolyn J. Marentes Ruiz, and Pia S. Pannaraj. 'Coronavirus Disease 2019 Vaccine Booster Effects Are Seen in Human Milk Antibody Response'. *Frontiers in Nutrition* 9 (2022). https://doi.org/10.3389/fnut.2022.898849.
Key 34	Bertrand, Kerri, Gordon Honerkamp-Smith, and Christina D. Chambers. 'Maternal and Child Outcomes Reported by Breastfeeding Women Following Messenger RNA COVID-19 Vaccination'. *Breastfeeding Medicine* 16, no. 9 (September 2021): 697–701. https://doi.org/10.1089/bfm.2021.0169.
Key 35	Cabanillas-Bernal, Olivia, Karla Cervantes-Luevano, Gonzalo Isai Flores-Acosta, Johanna Bernáldez-Sarabia, and Alexei F. Licea-Navarro. 'COVID-19 Neutralizing Antibodies in Breast Milk of Mothers Vaccinated with Three Different Vaccines in Mexico'. *Vaccines* 10, no. 4 (April 2022): 629. https://doi.org/10.3390/vaccines10040629.

Key 36	Calvo-Lerma, Joaquim, Pierre Bueno-Llamoga, Christine Bäuerl, Erika Cortés-Macias, Marta Selma-Royo, Francisco Pérez-Cano, Carles Lerin, Cecilia Martínez-Costa, and Maria Carmen Collado. 'Persistence of Anti SARS-CoV-2 Antibodies in Breast Milk from Infected and Vaccinated Women after In Vitro-Simulated Gastrointestinal Digestion'. *Nutrients* 14, no. 10 (January 2022): 2117. https://doi.org/10.3390/nu14102117.
Key 37	Charepe, Nadia, Juliana Gonçalves, A. Margarida Juliano, David G. Lopes, Helena Canhão, Helena Soares, and e. Fátima Serrano. 'COVID-19 mRNA Vaccine and Antibody Response in Lactating Women: A Prospective Cohort Study'. *BMC Pregnancy and Childbirth* 21, no. 1 (17 September 2021): 632. https://doi.org/10.1186/s12884-021-04051-6.
Key 38	Collier, Ai-ris Y., Katherine McMahan, Jingyou Yu, Lisa H. Tostanoski, Ricardo Aguayo, Jessica Ansel, Abishek Chandrashekar, et al. 'Immunogenicity of COVID-19 mRNA Vaccines in Pregnant and Lactating Women'. *JAMA* 325, no. 23 (15 June 2021): 2370–80. https://doi.org/10.1001/jama.2021.7563.
Key 39	Conti, Maria Giulia, Sara Terreri, Gianluca Terrin, Fabio Natale, Carlo Pietrasanta, Guglielmo Salvatori, Roberto Brunelli, et al. 'Severe Acute Respiratory Syndrome Coronavirus 2 Infection Versus Vaccination in Pregnancy: Implications for Maternal and Infant Immunity'. *Clinical Infectious Diseases* 75, no. Supplement_1 (15 August 2022): S37–45. https://doi.org/10.1093/cid/ciac359.
Key 40	Diogo, Patrícia, Gil Correia, João B. Martins, Rui Soares, Paulo J. Palma, João Miguel Santos, and Teresa Gonçalves. 'Delayed Cutaneous Adverse Reaction of the AstraZeneca COVID-19 Vaccine in a Breastfed Female Infant: A Coincidence or a Rare Effect?' *Vaccines* 10, no. 4 (April 2022): 602. https://doi.org/10.3390/vaccines10040602.
Key 41	Doughty, Hayden, and Dorothea Barton. 'Severe Nonanaphylactic Allergic Reaction to the Pfizer-BioNTech COVID-19 Vaccine'. *JAAD Case Reports* 19 (1 January 2022): 84–86. https://doi.org/10.1016/j.jdcr.2021.11.015.
Key 42	Esteve-Palau, Erika, Araceli Gonzalez-Cuevas, M. Eugenia Guerrero, Clara Garcia-Terol, M. Carmen Alvarez, David Casadevall, and Vicens Diaz-Brito. 'Quantification of Specific Antibodies Against SARS-CoV-2 in Breast Milk of Lactating Women Vaccinated With an mRNA Vaccine'. *JAMA Network Open* 4, no. 8 (11 August 2021): e2120575. https://doi.org/10.1001/jamanetworkopen.2021.20575.
Key 43	Golan, Yarden, Mary Prahl, Arianna G. Cassidy, Caryl Gay, Alan H. B. Wu, Unurzul Jigmeddagva, Christine Y. Lin, et al. 'COVID-19 mRNA Vaccination in Lactation: Assessment of Adverse Events and Vaccine Related Antibodies in Mother-Infant Dyads'. *Frontiers in Immunology* 12 (2021). https://www.frontiersin.org/articles/10.3389/fimmu.2021.777103.
Key 44	Golan, Yarden, Mary Prahl, Arianna Cassidy, Christine Y. Lin, Nadav Ahituv, Valerie J. Flaherman, and Stephanie L. Gaw. 'Evaluation of Messenger RNA From COVID-19 BTN162b2 and mRNA-1273 Vaccines in Human Milk'. *JAMA Pediatrics* 175, no. 10 (1 October 2021): 1069–71. https://doi.org/10.1001/jamapediatrics.2021.1929.
Key 45	Gonçalves, Juliana, A. Margarida Juliano, Nádia Charepe, Marta Alenquer, Diogo Athayde, Filipe Ferreira, Margarida Archer, Maria João Amorim, Fátima Serrano, and Helena Soares. 'Secretory IgA and T Cells Targeting SARS-CoV-2 Spike Protein Are Transferred to the Breastmilk upon mRNA Vaccination'. *Cell Reports Medicine* 2, no. 12 (21 December 2021). https://doi.org/10.1016/j.xcrm.2021.100468.

Key 46	Gray, Kathryn J., Evan A. Bordt, Caroline Atyeo, Elizabeth Deriso, Babatunde Akinwunmi, Nicola Young, Aranxta Medina Baez, et al. 'Coronavirus Disease 2019 Vaccine Response in Pregnant and Lactating Women: A Cohort Study'. *American Journal of Obstetrics & Gynecology* 225, no. 3 (1 September 2021): 303.e1-303.e17. https://doi.org/10.1016/j.ajog.2021.03.023.
Key 47	Henle, Andrea M. 'Increase in SARS-CoV-2 RBD-Specific IgA and IgG Antibodies in Human Milk From Lactating Women Following the COVID-19 Booster Vaccination'. *Journal of Human Lactation* 39, no. 1 (1 February 2023): 51–58. https://doi.org/10.1177/08903344221134631.
Key 48	İremli, Burçin Gönül, Süleyman Nahit Şendur, and Uğur Ünlütürk. 'Three Cases of Subacute Thyroiditis Following SARS-CoV-2 Vaccine: Postvaccination ASIA Syndrome'. *The Journal of Clinical Endocrinology & Metabolism* 106, no. 9 (1 September 2021): 2600–2605. https://doi.org/10.1210/clinem/dgab373.
Key 49	Jakuszko, Katarzyna, Katarzyna Kościelska-Kasprzak, Marcelina Żabińska, Dorota Bartoszek, Paweł Poznański, Dagna Rukasz, Renata Kłak, Barbara Królak-Olejnik, and Magdalena Krajewska. 'Immune Response to Vaccination against COVID-19 in Breastfeeding Health Workers'. *Vaccines* 9, no. 6 (June 2021): 663. https://doi.org/10.3390/vaccines9060663.
Key 50	Juncker, HG, SJ Mulleners, M. J. van Gils, T. P. L. Bijl, P. D. Pajkrt, A. Korosi, J. B. V. Goudoever, and B. J. V. Keulen. 'Comparison of Sars-Cov-2-Specific Antibodies in Human Milk after Mrna-Based Covid-19 Vaccination and Infection'. *Vaccines* 9, no. 12 (January 2021). https://doi.org/10.3390/vaccines9121475.
Key 51	Juncker, H. G., S. J. Mulleners, M. J. van Gils, C. J. M. de Groot, D. Pajkrt, A. Korosi, J. B. van Goudoever, and B. J. van Keulen. 'The Levels of SARS-CoV-2 Specific Antibodies in Human Milk Following Vaccination'. *Journal of Human Lactation* 37, no. 3 (1 August 2021): 477–84. https://doi.org/10.1177/08903344211027112.
Key 52	Juncker, H. G., S. J. Mulleners, E. J. M. Ruhé, E. R. M. Coenen, Sjors Bakker, M. van Doesburg, J. E. Harinck, et al. 'Comparing the Human Milk Antibody Response after Vaccination with Four COVID-19 Vaccines: A Prospective, Longitudinal Cohort Study in the Netherlands'. *EClinicalMedicine* 47 (May 2022): 101393. https://doi.org/10.1016/j.eclinm.2022.101393.
Key 53	Kachikis, Alisa, Janet A. Englund, Michael Singleton, Isabela Covelli, Alison L. Drake, and Linda O. Eckert. 'Short-Term Reactions Among Pregnant and Lactating Individuals in the First Wave of the COVID-19 Vaccine Rollout'. *JAMA Network Open* 4, no. 8 (17 August 2021): e2121310. https://doi.org/10.1001/jamanetworkopen.2021.21310.
Key 54	Lee, Youri, Gabrielle Grubbs, Sabrina C. Ramelli, Andrea R. Levine, Allison Bathula, Kapil Saharia, Madeleine Purcell, et al. 'SARS-CoV-2 mRNA Vaccine Induced Higher Antibody Affinity and IgG Titers against Variants of Concern in Post-Partum vs Non-Post-Partum Women'. *eBioMedicine* 77 (1 March 2022). https://doi.org/10.1016/j.ebiom.2022.103940.
Key 55	Low, Jia Ming, Yue Gu, Melissa Shu Feng Ng, Zubair Amin, Le Ye Lee, Yvonne Peng Mei Ng, Bhuvaneshwari D/O Shunmuganathan, et al. 'Codominant IgG and IgA Expression with Minimal Vaccine mRNA in Milk of BNT162b2 Vaccinees'. *Npj Vaccines* 6, no. 1 (19 August 2021): 1–8. https://doi.org/10.1038/s41541-021-00370-z.

Key 56	Low, Jia Ming, Le Ye Lee, Yvonne Peng Mei Ng, Youjia Zhong, and Zubair Amin. ‘Breastfeeding Mother and Child Clinical Outcomes After COVID-19 Vaccination’. *Journal of Human Lactation: Official Journal of International Lactation Consultant Association* 38, no. 1 (February 2022): 37–42. https://doi.org/10.1177/08903344211056522.
Key 57	Low, Jia Ming, Yue Gu, Melissa Shu Feng Ng, Liang Wei Wang, Zubair Amin, Youjia Zhong, and Paul A. MacAry. ‘Human Milk Antibodies after BNT162b2 Vaccination Exhibit Reduced Binding against SARS-CoV-2 Variants of Concern’. *Vaccines* 10, no. 2 (February 2022): 225. https://doi.org/10.3390/vaccines10020225.
Key 58	Mulleners, Sien J., Hannah G. Juncker, Marit J. van Gils, Johannes B. van Goudoever, and Britt J. van Keulen. ‘Human Milk Antibody Response After Combining Two Different COVID-19 Vaccines: Mix-and-Match’. *Journal of Human Lactation* 38, no. 3 (1 August 2022): 401–6. https://doi.org/10.1177/08903344221103260.
Key 59	Narayanaswamy, Vignesh, Brian T. Pentecost, Corina N. Schoen, Dominique Alfandari, Sallie S. Schneider, Ryan Baker, and Kathleen F. Arcaro. ‘Neutralizing Antibodies and Cytokines in Breast Milk After Coronavirus Disease 2019 (COVID-19) mRNA Vaccination’. *Obstetrics & Gynecology* 139, no. 2 (February 2022): 181. https://doi.org/10.1097/AOG.0000000000004661.
Key 60	Perez, Stephanie E., Luis Diego Luna Centeno, Wesley A. Cheng, Carolyn Jennifer Marentes Ruiz, Yesun Lee, Zion Congrave-Wilson, Rebecca L. Powell, Lisa Stellwagen, and Pia S. Pannaraj. ‘Human Milk SARS-CoV-2 Antibodies up to 6 Months After Vaccination’. *Pediatrics* 149, no. 2 (4 January 2022): e2021054260. https://doi.org/10.1542/peds.2021-054260.
Key 61	Rosenberg-Friedman, Michal, Aya Kigel, Yael Bahar, Michal Werbner, Joel Alter, Yariv Yogev, Yael Dror, et al. ‘BNT162b2 mRNA Vaccine Elicited Antibody Response in Blood and Milk of Breastfeeding Women’. *Nature Communications* 12, no. 1 (28 October 2021): 6222. https://doi.org/10.1038/s41467-021-26507-1.
Key 62	Schwartz, Anat, Omer Nir, Shlomi Toussia-Cohen, Leah Leibovich, Tzipora Strauss, Keren Asraf, Ram Doolman, et al. ‘Presence of SARS-CoV-2 Antibodies in Lactating Women and Their Infants Following BNT162b2 Messenger RNA Vaccine’. *American Journal of Obstetrics & Gynecology* 225, no. 5 (1 November 2021): 577–79. https://doi.org/10.1016/j.ajog.2021.07.016.
Key 63	Selma-Royo, Marta, Christine Bäuerl, Desirée Mena-Tudela, Laia Aguilar-Camprubí, Francisco J. Pérez-Cano, Anna Parra-Llorca, Carles Lerin, Cecilia Martínez-Costa, and Maria Carmen Collado. ‘Anti-SARS-CoV-2 IgA and IgG in Human Milk after Vaccination Is Dependent on Vaccine Type and Previous SARS-CoV-2 Exposure: A Longitudinal Study’. *Genome Medicine* 14, no. 1 (21 April 2022): 42. https://doi.org/10.1186/s13073-022-01043-9.
Key 64	Stafford, Lauren Stewart, Vivian Valcarce Luaces, Joseph Neu, Nicole Cacho, Leslie Parker, David Burchfield, Nan Li, and Joseph Larkin. ‘Effect of SARS-CoV-2 Vaccine on the Breastmilk Antibody Response among Lactating Healthcare Workers’. *The Journal of Immunology* 206, no. 1_Supplement (1 May 2021): 30.15. https://doi.org/10.4049/jimmunol.206.Supp.30.15.

Key 65	Trofin, Felicia, Eduard Vasile Nastase, Luminita Smaranda Iancu, Daniela Constantinescu, Corina Maria Cianga, Catalina Lunca, Ramona Gabriela Ursu, Petru Cianga, and Olivia Simona Dorneanu. 'Anti-RBD IgA and IgG Response and Transmission in Breast Milk of Anti-SARS-CoV-2 Vaccinated Mothers'. *Pathogens* 11, no. 3 (March 2022): 286. https://doi.org/10.3390/pathogens11030286.
Key 66	Valcarce, Vivian, Lauren Stewart Stafford, Josef Neu, Nicole Cacho, Leslie Parker, Martina Mueller, David J. Burchfield, Nan Li, and Joseph Larkin. 'Detection of SARS-CoV-2-Specific IgA in the Human Milk of COVID-19 Vaccinated Lactating Health Care Workers'. *Breastfeeding Medicine: The Official Journal of the Academy of Breastfeeding Medicine* 16, no. 12 (December 2021): 1004–9. https://doi.org/10.1089/bfm.2021.0122.
Key 67	Wang, Jiong, Bridget E. Young, Dongmei Li, Antti Seppo, Qian Zhou, Alexander Wiltse, Anna Nowak-Wegrzyn, et al. 'Broad Cross-Reactive IgA and IgG against Human Coronaviruses in Milk Induced by COVID-19 Vaccination and Infection'. *Vaccines* 10, no. 6 (June 2022): 980. https://doi.org/10.3390/vaccines10060980.
Key 68	Yang, Xiaoqi, Alisa Fox, Claire DeCarlo, Caroline Norris, Samantha Griffin, Sophie Wedekind, James M. Flanagan, Natalie Shenker, and Rebecca L. Powell. 'Comparative Profiles of SARS-CoV-2 Spike-Specific Human Milk Antibodies Elicited by mRNA- and Adenovirus-Based COVID-19 Vaccines'. *Breastfeeding Medicine* 17, no. 8 (August 2022): 638–46. https://doi.org/10.1089/bfm.2022.0019.
Key 69	Yeo, Kee Thai, Wan Ni Chia, Chee Wah Tan, Chengsi Ong, Joo Guan Yeo, Jinyan Zhang, Su Li Poh, et al. 'Neutralizing Activity and SARS-CoV-2 Vaccine mRNA Persistence in Serum and Breastmilk After BNT162b2 Vaccination in Lactating Women'. *Frontiers in Immunology* 12 (2022). https://doi.org/10.3389/fimmu.2021.783975.
Key 70	Young, Bridget E., Antti E. Seppo, Nichole Diaz, Casey Rosen-Carole, Anna Nowak-Wegrzyn, Joseline M. Cruz Vasquez, Rita Ferri-Huerta, et al. 'Association of Human Milk Antibody Induction, Persistence, and Neutralizing Capacity With SARS-CoV-2 Infection vs mRNA Vaccination'. *JAMA Pediatrics* 176, no. 2 (1 February 2022): 159–68. https://doi.org/10.1001/jamapediatrics.2021.4897.
Key 71	Tingle, A. J., L. A. Mitchell, M. Grace, P. Middleton, R. Mathias, L. MacWilliam, and A. Chalmers. 'Randomised Double-Blind Placebo-Controlled Study on Adverse Effects of Rubella Immunisation in Seronegative Women'. *Lancet (London, England)* 349, no. 9061 (3 May 1997): 1277–81. https://doi.org/10.1016/S0140-6736(96)12031-6.
Key 72	Kluthe, Margaret, Angel Herrera, Haydee Blanca, Jessica Leung, Stephanie R. Bialek, and D. Scott Schmid. 'Neonatal Vaccine-Strain Varicella-Zoster Virus Infection 22 Days After Maternal Postpartum Vaccination'. *The Pediatric Infectious Disease Journal* 31, no. 9 (September 2012): 977. https://doi.org/10.1097/INF.0b013e31825d2a1b.
Key 73	Saringkarisate, Kornkanok, Kyra A. Len, Marian E. Melish, Bryscen K. Prothero, and Natascha Ching. 'Vaccine-Strain Varicella Virus Transmitted to a Term Infant Following Maternal Postpartum Vaccination'. *Journal of the Pediatric Infectious Diseases Society* 11, no. 10 (25 October 2022): 452–53. https://doi.org/10.1093/jpids/piac050.
